# Transcriptional Profiling of the Bladder in Urogenital Schistosomiasis Reveals Pathways of Inflammatory Fibrosis and Urothelial Compromise

**DOI:** 10.1371/journal.pntd.0001912

**Published:** 2012-11-29

**Authors:** Debalina Ray, Tyrrell A. Nelson, Chi-Ling Fu, Shailja Patel, Diana N. Gong, Justin I. Odegaard, Michael H. Hsieh

**Affiliations:** 1 Department of Urology, Stanford University School of Medicine, Stanford, California, United States of America; 2 Department of Pathology, University of California San Francisco, San Francisco, California, United States of America; 3 Department of Pathology, Stanford University School of Medicine, Stanford, California, United States of America; Uniformed Services University, United States of America

## Abstract

Urogenital schistosomiasis, chronic infection by *Schistosoma haematobium*, affects 112 million people worldwide. *S. haematobium* worm oviposition in the bladder wall leads to granulomatous inflammation, fibrosis, and egg expulsion into the urine. Despite the global impact of urogenital schistosomiasis, basic understanding of the associated pathologic mechanisms has been incomplete due to the lack of suitable animal models. We leveraged our recently developed mouse model of urogenital schistosomiasis to perform the first-ever profiling of the early molecular events that occur in the bladder in response to the introduction of *S. haematobium* eggs. Microarray analysis of bladders revealed rapid, differential transcription of large numbers of genes, peaking three weeks post-egg administration. Many differentially transcribed genes were related to the canonical Type 2 anti-schistosomal immune response, as reflected by the development of egg-based bladder granulomata. Numerous collagen and metalloproteinase genes were differentially transcribed over time, revealing complex remodeling and fibrosis of the bladder that was confirmed by Masson's Trichrome staining. Multiple genes implicated in carcinogenesis pathways, including vascular endothelial growth factor-, oncogene-, and mammary tumor-related genes, were differentially transcribed in egg-injected bladders. Surprisingly, junctional adhesion molecule, claudin and uroplakin genes, key components for maintaining the urothelial barrier, were globally suppressed after bladder exposure to eggs. This occurred in the setting of urothelial hyperplasia and egg shedding in urine. Thus, *S. haematobium* egg expulsion is associated with intricate modulation of the urothelial barrier on the cellular and molecular level. Taken together, our findings have important implications for understanding host-parasite interactions and carcinogenesis in urogenital schistosomiasis, and may provide clues for novel therapeutic strategies.

## Author Summary

Parasitic *Schistosoma haematobium* worms cause urogenital schistosomiasis in 112 million people worldwide. These worms lay eggs in the bladder wall, resulting in inflammation, fibrosis (internal scarring), bladder cancer, and passage of eggs into the urine. Indeed, the International Agency for Research on Cancer within the World Health Organization has classified *S. haematobium* as a “Class I” agent (“Carcinogenic to humans”). Moreover, *S. haematobium*-induced fibrosis and resulting obstructive kidney failure leads to 150,000 deaths annually. As a result, *S. haematobium* infection is one of the most important causes of worm-related death globally. In spite of this, research on this parasite is sparse due to a lack of suitable animal models. We have used our recently developed mouse model of urogenital schistosomiasis to understand the global bladder gene response to this infection. Large numbers of genes featured differential transcription after experimental infection, including specific immune response-, fibrosis-, cancer-, and bladder function-related genes. The relevance of these gene-based findings was verified through microscopic examination of egg-exposed bladders. Our data will improve our comprehension of urogenital schistosomiasis, and may help identify new targets for diagnosis and treatment of this disease, and possibly bladder cancer and bladder-based inflammatory disorders as well.

## Introduction

Schistosomiasis, chronic infection with parasitic *Schistosoma* worms, affects at least 200 million people worldwide, and may rival malaria in terms of socioeconomic impact in endemic regions [1]. The two main forms of schistosomiasis are hepatoenteric and urogenital, caused primarily by *Schistosoma mansoni* and *Schistosoma haematobium*, respectively. In both forms of schistosomiasis, the tropism of adult worms for particular vascular beds (mesenteric or pelvic) determines where oviposition occurs. Deposited eggs trigger florid tissue inflammation that is believed to cause much of the morbidity of infection. For example, *S. haematobium* oviposition in the bladder and genital tract results in hematuria, urinary tract fibrosis (sometimes leading to obstructive renal failure), increased susceptibility to HIV infection, and enhanced risk of bladder cancer. Van der Werf et al. estimated that in a 2 week period in 2000, 70 and 32 million individuals in sub-Saharan Africa experienced hematuria and dysuria associated with *S. haematobium* infection, respectively [2]. Significant *S. haematobium*-triggered bladder wall pathology and severe hydronephrosis were predicted to afflict 18 and 10 million people, respectively. Urogenital schistosomiasis appears to predispose individuals to earlier onset and more aggressive bladder cancers [3], [4]. Moreover, an estimated 150,000 deaths are attributable annually to *S. haematobium*-induced obstructive renal failure alone. Consequently, urogenital schistosomiasis is one of the most important causes of helminth-related mortality worldwide.

Despite the importance of this infection, the mechanisms by which urogenital schistosomiasis leads to these sequelae are poorly defined, particularly in the early stages of infection. This deficiency in knowledge is due largely to the lack of suitable animal models for *S. haematobium* infection [5]. Natural transdermal infection of mice or other rodents (e.g., hamsters) with *S. haematobium* cercariae typically results in hepatoenteric rather than urogenital disease [6]–[9]. Non-human primates recapitulate human disease but are expensive and controversial to use as animal models [10]. Both hamster and primate models suffer from having few species-specific tools.

To address the dearth of good animal models for urogenital schistosomiasis we recently developed a novel mouse model of *S. haematobium* egg-induced immunopathology [11]. In this model, a single direct injection of *S. haematobium* eggs into the bladder walls of mice recapitulates multiple aspects of human disease, including a regional and systemic Type 2 immune response, development of bladder granulomata, hematuria, bladder fibrosis, egg shedding, and urothelial hyperplasia. We sought to leverage this model to determine the early molecular events in the bladder occurring after introduction of *S. haematobium* eggs, as well as the temporal evolution of these processes. Microarray analysis demonstrated time-dependent, differential transcription of large numbers of genes. Many differentially transcribed genes were related to the canonical Type 2 anti-schistosomal immune response, collagen and metalloproteinase activity, urothelial barrier functions, and carcinogenesis pathways. Taken together, our findings have important implications for understanding host-parasite interactions and carcinogenesis in urogenital schistosomiasis, and may provide clues for novel therapeutic strategies for this disease and perhaps bladder cancer and bladder inflammatory disorders in general.

## Materials and Methods

### Ethics statement

All animal work was conducted according to relevant U.S. and international guidelines. Specifically, all experimental procedures were carried out in accordance with the Administrative Panel on Laboratory Animal Care (APLAC) protocol and the institutional guidelines set by the Veterinary Service Center at Stanford University (Animal Welfare Assurance A3213-01 and USDA License 93-4R-00). Stanford APLAC and institutional guidelines are in compliance with the U.S. Public Health Service Policy on Humane Care and Use of Laboratory Animals. The Stanford APLAC approved the animal protocol associated with the work described in this publication.

### *S. haematobium* egg isolation

*S. haematobium*-infected LVG hamsters were obtained from the National Institute of Allergy and Infectious Diseases Schistosomiasis Resource Center of the National Institutes of Health. Eggs were isolated from hamsters as previously described [11]. In brief, hamsters were sacrificed at 18 weeks post-infection, at which time livers and intestines were minced, homogenized in a Waring blender, resuspended in 1.2% NaCl containing antibiotic-antimycotic solution (100 units Penicillin, 100 µg/mL Streptomycin and 0.25 µg/mL Amphotericin B, Sigma-Aldrich), passed through a series of stainless steel sieves with sequentially decreasing pore sizes (450 µm, 180 µm, and 100 µm), and finally retained on a 45 µm sieve.

### *S. haematobium* egg injection

Egg injections of the mouse bladder wall were performed as previously described [11], [12]. Specifically, 7 to 8 week-old female BALB/c mice (Jackson Laboratories) were anesthetized with isoflurane, a midline lower abdominal incision was made, and the bladder exteriorized. Freshly prepared *S. haematobium* eggs (3,000 eggs in 50 µl of phosphate-buffered saline, experimental group) or uninfected hamster liver and intestinal extract (in 50 µl of phosphate-buffered saline, control group) was injected submucosally into the anterior aspect of the bladder dome. Abdominal incisions were then closed with 4-0 Vicryl suture, and the surgical site was treated once with topical antibiotic ointment. Mice were sacrificed at 1, 3, and 5 weeks post-injection (n = 3 for each time point/treatment group [egg vs. vehicle])

### Micro-ultrasonography

Mouse bladder micro-ultrasonography was performed as previously described [11]. At various time points after bladder wall injection, mice were anesthetized using vaporized isoflurane and their abdominal walls were depilated. Transabdominal images of the bladder were then obtained using a VisualSonics Vevo 770 high-resolution ultrasound micro-imaging system with an RMV 704 scanhead [40 MHz] (Small Animal Imaging Facility, Stanford Center for Innovation in In-Vivo Imaging).

### Bladder histopathologic analysis

Mice were sacrificed after bladder wall injection, and bladders processed for routine histology. Five µm sections were stained with hematoxylin and eosin or Masson's Trichrome-stained sections.

### Arginase-1 immunohistochemistry

Bladders were removed from mice and placed in 10% phosphate-buffered formalin for 18 hours before dehydration and embedding in paraffin. Serial 5 µm sections were cut using a microtome, placed on positively charged glass slides and dried overnight at 56°C. Sections were heated to 100°C in citric acid buffer (pH 6.0) for 15 minutes. Endogenous peroxidase activity was blocked using 0.3% hydrogen peroxidase in methanol. An avidin-biotin complex immunoperoxidase protocol was employed, including staining of sections with anti-arginase-1 antibody (Clone 19/Arginase I, BD Biosciences) at a dilution of 1∶1000, biotinylated anti-mouse IgG1 at 1∶1000(BioLegend). Next, sections were incubated with streptavidin labeled horseradish peroxidase (Biocare Medical, Concord, CA) followed by use of a DAB chromogen substrate kit (Biocare Medical, Concord, CA) and counterstaining with hematoxylin.

### Bladder microarray analysis

Mice were sacrificed and bladders were immediately collected and preserved in RNA Later (Ambion) at −80°C. RNA was extracted for each individual bladder by Trizol Reagent (Ambion) and was reverse transcribed to cDNA. RNA yields were measured using a QuBit 2.0 Fluorimeter (Life Technologies, Grand Island, NY, USA) and quality was assessed using an Agilent Bioanalyzer and RNA 6000 Nano Labchips (Agilent Technologies, Foster City, USA). Next, individual bladder cDNA was *in vitro* transcribed to synthesize cRNA using Illumina TotalPrep RNA Amplification Kits (Ambion, Applied Biosystems, Foster City, CA), and hybridized using standard Illumina protocols on the MouseWG-6 v2.0 chip (Stanford Functional Genomics Facility, Stanford, CA). Illumina Beadstation-generated scanned array files were filtered and normalized (quantile method) in GeneSpring GX version 11. Genes in egg- versus control vehicle-injected bladders were considered differentially transcribed at various time points if they were transcribed ≥2-fold and p<0.05 by unpaired T-test. Functional annotation of differentially transcribed genes was performed using DAVID (DAVID Bioinformatics Resources 6.7, National Institute of Allergy and Infectious Diseases [NIAID], NIH) in combination with the Biocarta and KEGG pathway databases [13], [14].

### Real-time PCR

Mice were sacrificed after bladder wall injection, and bladder RNA preserved and isolated as above. RNA yields and quality were measured as above. cDNA was synthesized from the RNA of individual mouse bladders. Primer sequences for genes of interest were obtained from PrimerBank (http://pga.mgh.harvard.edu/primerbank/). (Table S3) GAPDH was used as a housekeeping gene. Real-time PCR was performed using SYBR Green and an Mx3005p thermal cycler (Stratagene). Cycle thresholds (Ct) were calculated for each reaction. Using the comparative Ct method relative gene transcription was calculated as 2^−ΔΔCt^, where ΔCt = Ct (gene of interest) - ΔCt (normalizer = β-actin). ΔΔCt was calculated as ΔCt (egg-injected) - ΔCt (calibrator). Correlations between microarray and real-time PCR results were assessed using Spearman's Rho measure of correlation in Microsoft Excel 2010 for Windows.

## Results

### Delivery of *S. haematobium* eggs to the bladder triggers time-dependent, differential gene transcription

**Figure 1:**
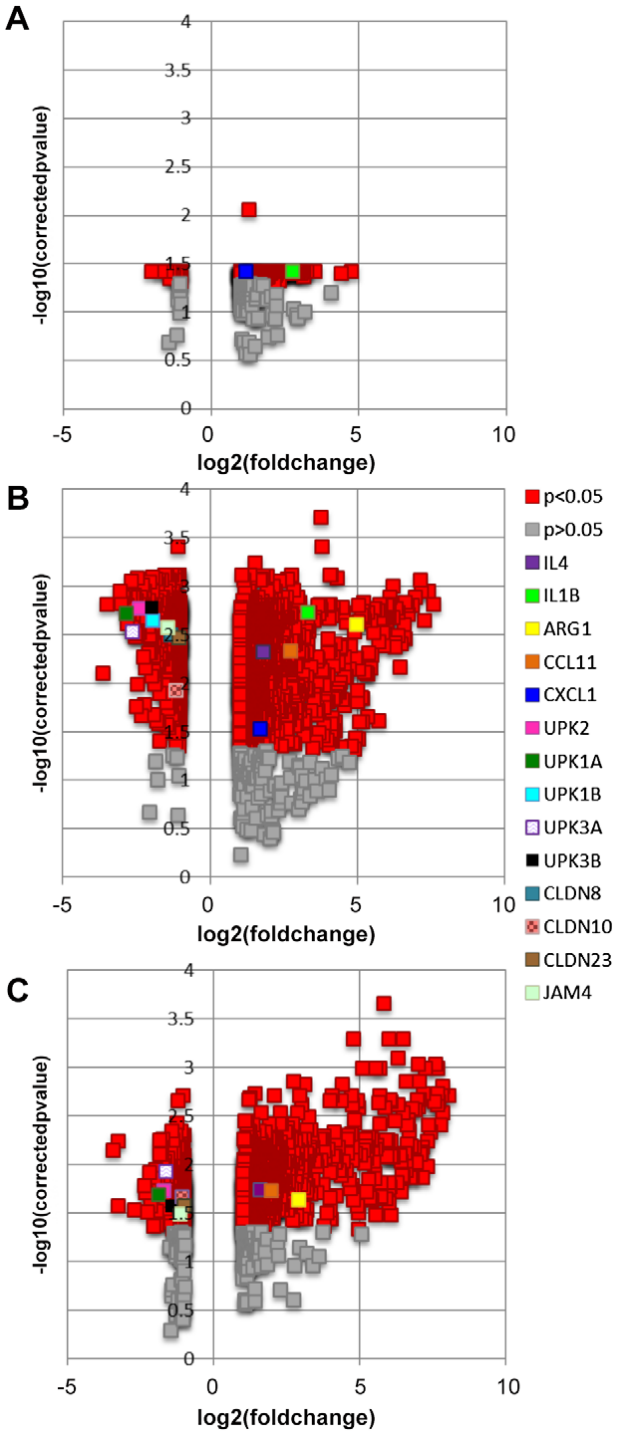
Volcano plots of differentially transcribed genes in egg-injected bladders. Separate plots are shown comparing microarray-derived gene transcription in egg-injected bladders relative to control vehicle-injected bladders (A, one week post-injection; B, three weeks post-injection; C, five weeks post-injection). Log fold changes are plotted on the x-axes and negative log10 p-values are plotted on the y-axes. Red and other non-dark gray, colored symbols denote statistically significant (p<0.05) changes in gene transcription. Gray symbols denote statistically insignificant (p>0.05) changes in change transcription. Legend for individual, selected genes of interest applies to all three panels. For clarity, each selected gene of interest with multiple microarray probes has been denoted using the probe featuring the greatest differential signal for that gene.

Microarray-based comparisons of *S. haematobium* egg- versus control vehicle-injected bladders demonstrated differential gene transcription over time (Figure 1 and Table S1). At one week post-egg injection, 279 and 22 genes featured significantly more and less transcription (≥2-fold and p<0.05), respectively. By three weeks post-injection, more genes were differentially transcribed, with 1001 and 570 genes demonstrating more and less transcription, respectively. At five weeks post-injection, fewer genes demonstrated altered transcription, with 794 and 308 genes exhibiting more and less transcription, respectively. Functional annotation clustering of genes featuring ≥2-fold differential transcription indicated that each of these gene clusters was associated with a discrete gene ontology (Table S2). Many of these clusters were related to immune responses. Illumina probe IDs and Entrez Gene IDs of selected genes are provided (Table S1).

**Figure 2:**
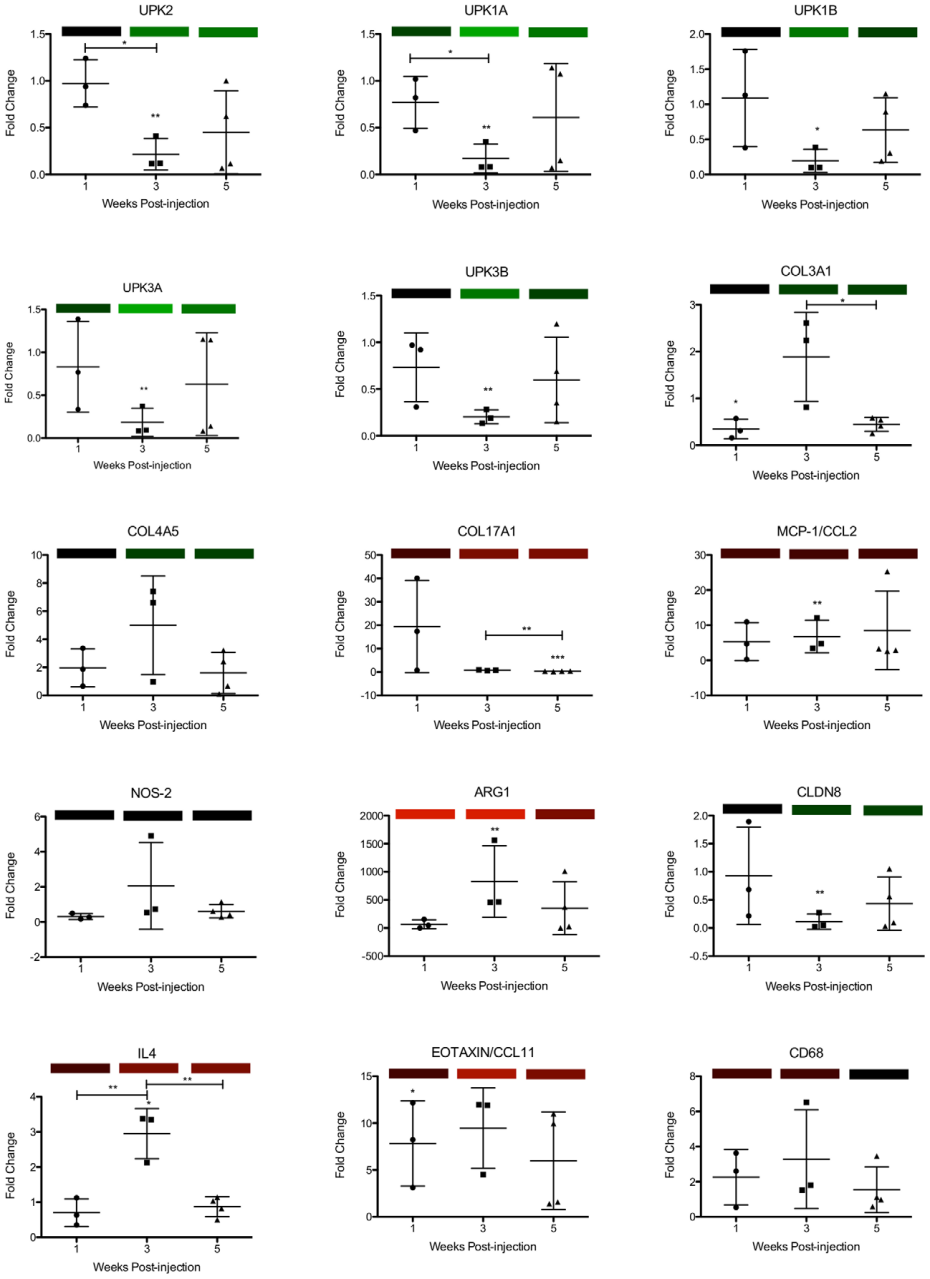
Real-time PCR corroboration of gene transcription profiles obtained by microarray analysis. Transcription of a subset of genes analyzed by real-time PCR is depicted in the scatter plots and is displayed as fold change relative to control-injected bladders at 1, 3 and 5 weeks post-injection. Colored bars correspond to microarray data, with decreased, increased, and unchanged transcription colored green, red, and black, respectively. Increasing intensity of green and red bars indicates more extreme changes in transcription (up to >20-fold). * = p<0.05, ** = p<0.01, ***p<0.001 in comparison to control vehicle-injected bladders (or across time points if annotated with brackets). UPK3A: uroplakin 3a, CLDN8: claudin-8, COL4A5: collagen type 4 alpha 5, COL17A1: collagen type 17 alpha 1, NOS2: inducible nitric oxide synthase, UPK3B: uroplakin 3b, UPK1B: uroplakin 1b, UPK1A: uroplakin 1a, UPK2: uroplakin 2, ARG1: arginase-1.

Validation of a subset of the microarray data was performed using real-time PCR (Figure 2). Specifically, genes related to inflammation (IL4, CCL2, CCL11, inducible nitric oxide synthase [iNOS], arginase-1, and CD68), urothelial function (uroplakins 1A, 1B, 2, 3A, and 3B and claudin-8), and collagen (collagen type 3α1, 4α5, and 17 α1) were assayed. Overall, correlations between microarray and PCR findings were tight, with Spearman's correlation r = 0.87, 0.94, and 0.84 at 1, 3, and 5 weeks post-injection, respectively.

### *S. haematobium* eggs induce bladder transcription of granulomatous, Type 2 inflammation-associated genes

Bladder wall injection with *S. haematobium* eggs induced increased transcription of numerous genes related to granulomatous inflammation and type 2 immunity (selected examples in Table 1). Specific genes which exhibited increased transcription included cytokine and cytokine-related genes such as IL-4, IL-4-induced 1, IL-1β, IL-6, interferon gamma-inducible proteins (IFI30 and IFI47), TGF-β, IL-13 receptor alpha 2, IL-10 receptor alpha, and cytokine inducible SH2-containing protein (CISH); markers of macrophages including macrophage-expressed gene 1 (MPEG1, general macrophage marker), and arginase, Ym1 (CHI3L3), and mannose receptor C type 1 (all alternatively activated macrophage markers); and chemokines such as CCL4 (MIP-1 beta), CCL5 (RANTES), CCL11 (eotaxin) and CXCL1 (KC). In fact, KEGG pathways analysis confirmed that one of the biological clusters featuring the greatest number of differentially transcribed genes at one and three weeks post-egg injection was “cytokine-cytokine receptor pathways” (1 week: 15 genes, 3 weeks: 41 genes, Tables 2 and 3). By 5 weeks post-egg injection, the biological cluster with the greatest number of differentially transcribed genes (26) was “B cell receptor signaling pathway” (Table 4). Accordingly, numerous IgG and IgE Fc receptor genes featured increased transcription. Other immune response-related genes exhibiting differential transcription included those encoding for eosinophil ribonucleases, C1q, arachidonate 15-lipoxygenase (ALOX15), and platelet thromboxane A synthase 1 (TBXAS1).

**Table 1:** Increased transcription of granulomatous and type 2 inflammation genes identified through microarray analysis.

Gene	Fold-Regulation Post-Injection Relative to Control*			
	Time Post-Injection	Week 1	Week 3	Week 5
IL4		**	3.48197	3.03925
IL4I1		4.62434	8.84372	9.386889
IL1B		6.73795	10.05383	**
IL6		**	3.294366	**
TGFB		**	2.2158	**
ARG1		**	21.9539	7.41649
CCL4		**	3.37419	**
CCL5		**	**	2.605253
CCL11		**	5.88034	3.112205
CXCL1		2.27438	3.28179	**
IFI30		**	2.545283	2.864982
IFI47		2.318303	2.252061	**
IL13RA2		**	5.486401	3.557954
IL10RA		2.014572	3.834927	3.070781
CISH		**	2.600125	**
ALOX15		**	3.397949	2.748778
TBXAS1		2.698519	2.63449	**
MPEG1		**	**	2.090888
CHI3L3		**	25.39334	6.99343
MRC1		2.973735	2.644192	**

* All values ≥2-fold and p<0.05.

** Value <2-fold and/or p≥0.05.

**Table 2:** KEGG pathways analysis reveals extensive cytokine-cytokine receptor interactions in the bladder one week post-egg exposure.

Entrez Gene ID	Gene Name
100048556	chemokine (C-C motif) ligand 12; similar to MCP-5
56221	chemokine (C-C motif) ligand 24
20306	chemokine (C-C motif) ligand 7
20308	chemokine (C-C motif) ligand 9
14825	chemokine (C-X-C motif) ligand 1
12978	colony stimulating factor 1 receptor
12984	CSF 2 receptor, beta 2, low-affinity (granulocyte-macrophage)
16178	interleukin 1 receptor, type II
16154	interleukin 10 receptor, alpha
16156	interleukin 11
12765	interleukin 8 receptor, beta
18413	oncostatin M
12986	predicted gene 4223; similar to Csf3r protein
100044702	similar to LPS-induced CXC chemokine; CXCL5
18383	TNF receptor superfamily, member 11b (osteoprotegerin)

All genes shown featured ≥2-fold differential transcription and p<0.05.

**Table 3:** KEGG pathways analysis reveals extensive cytokine-cytokine receptor interactions in the bladder three weeks post-egg exposure.

Entrez Gene ID	Gene Name
21940	CD27 antigen
21939	CD40 antigen
20292	chemokine (C-C motif) ligand 11
100048556	chemokine (C-C motif) ligand 12; similar to MCP-5
18829	chemokine (C-C motif) ligand 21A
20303	chemokine (C-C motif) ligand 4
20306	chemokine (C-C motif) ligand 7
20308	chemokine (C-C motif) ligand 9
12774	chemokine (C-C motif) receptor 5
12458	chemokine (C-C motif) receptor 6
12775	chemokine (C-C motif) receptor 7
14825	chemokine (C-X-C motif) ligand 1
66102	chemokine (C-X-C motif) ligand 16
80901	chemokine (C-X-C motif) receptor 6
12978	colony stimulating factor 1 receptor
12984	CSF 2 receptor, beta 2, low-affinity (granulocyte-macrophage)
12983	CSF 2 receptor, beta, low-affinity (granulocyte-macrophage)
16323	inhibin beta-A
16178	interleukin 1 receptor, type II
16154	interleukin 10 receptor, alpha
16156	interleukin 11
329244	interleukin 19
93672	interleukin 24
16189	interleukin 4 receptor, alpha
16190	interleukin 4
16193	interleukin 6
18053	nerve growth factor receptor(TNFR superfam., member 16)
18413	oncostatin M
100041504, 65956	similar to beta chemokine Exodus-2
24047	predicted chemokine (C-C motif) ligand 19
12986	similar to Csf3r protein
16186	predicted interleukin 2 receptor, gamma chain
57349	pro-platelet basic protein
100044702	similar to LPS-induced CXC chemokine; CXCL5
21803	transforming growth factor, beta 1
21943	tumor necrosis factor (ligand) superfamily, member 11
24099	tumor necrosis factor (ligand) superfamily, member 13b
72049	tumor necrosis factor receptor superfamily, member 13c
21935	tumor necrosis factor receptor superfamily, member 17
21936	tumor necrosis factor receptor superfamily, member 18
22163	tumor necrosis factor receptor superfamily, member 4

All genes shown featured ≥2-fold differential transcription and p<0.05.

**Table 4:** KEGG pathways analysis reveals differential transcription of multiple gene members of the B cell receptor pathway five weeks post-egg exposure.

ENTREZ GENE ID	GENE NAME
17060	B-cell linker
12229	Bruton agammaglobulinemia tyrosine kinase
12478	CD19 antigen
12483	CD22 antigen; hypothetical protein LOC100047973
12517	CD72 antigen
15985	CD79B antigen
14281	FBJ osteosarcoma oncogene
14130	Fc receptor, IgG, low affinity IIb
240168	RAS, guanyl releasing protein 3
19354	RAS-related C3 botulinum substrate 2
108723	caspase recruitment domain family, member 11
12902	complement receptor 2
380794	immunoglobulin heavy chain 3 (serum IgG2b); Ig heavy chain (gamma polypeptide)
16331	inositol polyphosphate-5-phosphatase D
68713	interferon induced transmembrane protein 1
240354	mucosa associated lymphoid tissue lymphoma translocation gene 1
18018	nuclear factor of activated T-cells, cytoplasmic, calcineurin-dependent 1
18037	nuclear factor of kappa light polypeptide gene enhancer in B-cells inhibitor, epsilon
18707	phosphatidylinositol 3-kinase catalytic delta polypeptide; RIKEN cDNA 2610208K16 gene
83490	phosphoinositide-3-kinase adaptor protein 1
30955	phosphoinositide-3-kinase, catalytic, gamma polypeptide
234779	phospholipase C, gamma 2
16061	predicted immunoglobulin heavy chain (J558 family)
19057	protein phosphatase 3, catalytic subunit, gamma isoform
15170	protein tyrosine phosphatase, non-receptor type 6
22324	vav 1 oncogene

All genes shown featured ≥2-fold differential transcription and p<0.05.

**Figure 3:**
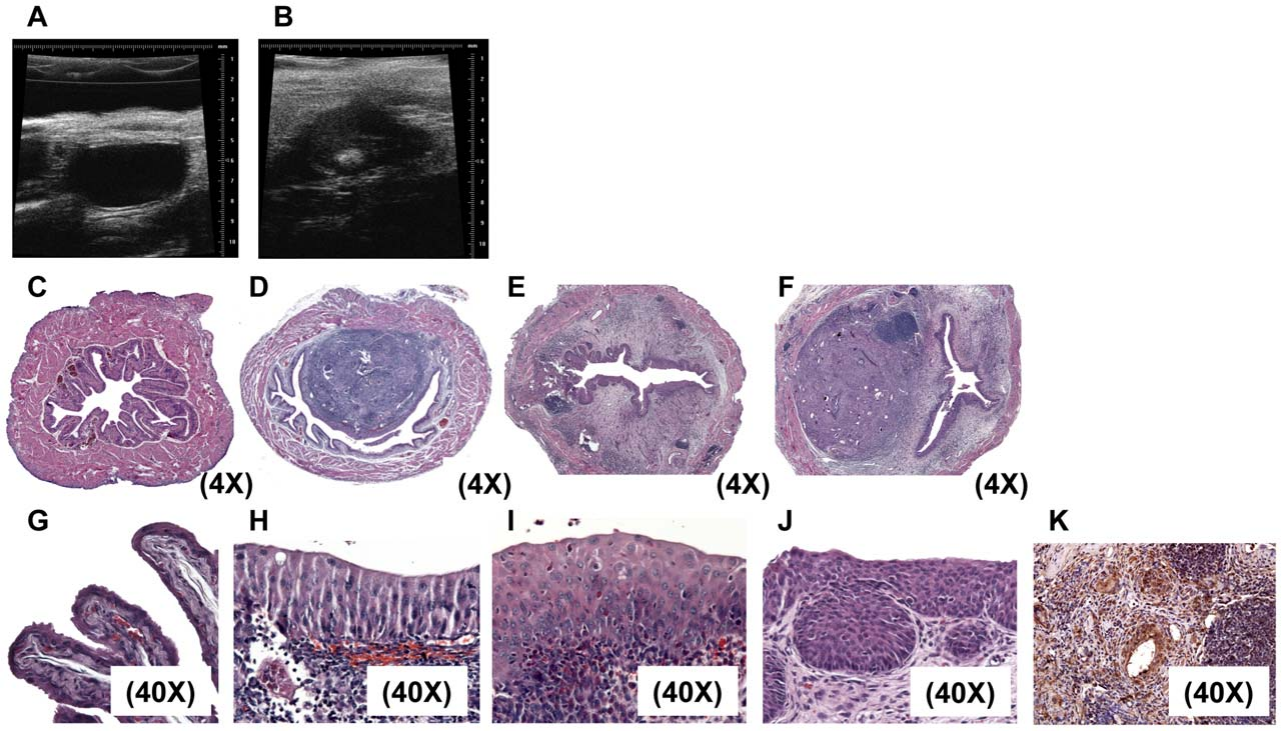
Bladder wall injection with *S. haematobium* eggs triggers granuloma growth and urothelial hyperplasia. Intramural injection of *S. haematobium* eggs results in granuloma development. A, micro-ultrasonography of a single representative animal injected with control vehicle showing no granuloma formation; B, micro-ultrasonography of a single representative animal injected with eggs, note the presence of a bright, echogenic round granuloma (denoted by white arrow). Intramural injection of *S. haematobium* eggs initiates histologically-evident granuloma formation by one week and persisting for over five weeks (D–F), while control vehicle injection does not result in granuloma formation (C). Intramural injection of *S. haematobium* eggs induces early and sustained urothelial hyperplasia with reactive nuclear changes (1, 3, and 5 weeks post-injection, H–J), whereas control vehicle injected-bladders feature normal appearing urothelium (G). Arginase-1-specific immunohistochemistry confirms widespread expression of the enzyme in the bladder 4 weeks post-egg injection (K).

Granuloma formation was confirmed *in vivo* by transabdominal bladder microultrasonography (Figure 3a–b) and histologically by hematoxylin and eosin staining of egg-injected bladders (Figure 3c–f). Finally, type 2 inflammation was verified through immunohistochemical staining for arginase-1, an enzyme strongly associated with type 2 immunity-mediated alternative activation of macrophages (Figure 3k).

### Introduction of *S. haematobium* eggs to the bladder elicits changes in urothelial function-related genes

**Figure 4:**
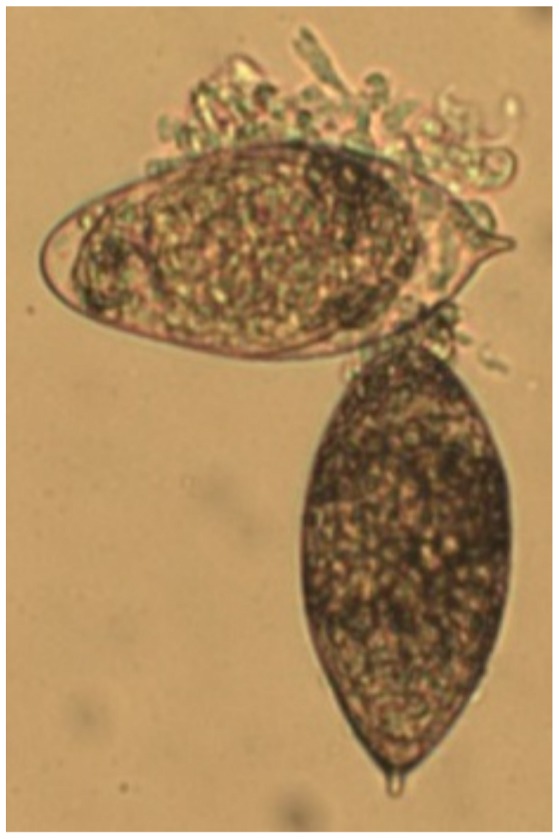
*S. haematobium* eggs are shed in the urine of bladder wall-injected mice. Micrograph showing two intact *S. haematobium* eggs isolated from the urine of a bladder wall-injected mouse 2 weeks post-egg injection.

Delivery of *S. haematobium* eggs to the bladder prompted global decreased transcription of all uroplakin genes, in addition to several tight junction-related genes, at three weeks post-egg injection (claudins and junctional adhesion molecule-4, Table 5). Interestingly, this occurred in the context of egg shedding in urine (Figure 4) and profound urothelial hyperplasia (Figure 3g–j), a precursor lesion for bladder cancer. Indeed, pathways analysis of microarray data implicated involvement of various carcinogenesis-related signaling pathways at 5 weeks post-injection, including vascular endothelial growth factor-, oncogene-, and mammary tumor-related genes (Tables 6, 7, 8).

**Table 5:** Microarray analysis reveals urothelial barrier function genes with less transcription after egg exposure.

Gene	Fold-Regulation Post-Injection Relative to Control*			
	Time Post-Injection	Week 1	Week 3	Week 5
UPK2		**	0.19028	0.303506
UPK1A		**	0.155542	0.311607
UPK1B		**	0.262438	**
UPK3A		**	0.159784	0.320718
UPK3B		**	0.281346	0.376946
CLDN8		**	0.398229	**
CLDN10		**	0.448328	0.46717
CLDN23		**	0.48318	0.495167
JAM4		**	0.374898	0.446032

* All values shown ≥2-fold and p<0.05.

** Value <2-fold and/or p≥0.05.

**Table 6:** Pathways analysis reveals differential transcription of multiple gene members of the vascular endothelial growth factor-related pathway five weeks post-egg exposure.

ENTREZ GENE ID	GENE NAME
19354	RAS-related C3 botulinum substrate 2
27371	SH2 domain protein 2A
15507	heat shock protein 1
18018	nuclear factor of activated T-cells, cytoplasmic, calcineurin-dependent 1
18707	phosphatidylinositol 3-kinase catalytic delta polypeptide; RIKEN cDNA 2610208K16 gene
30955	phosphoinositide-3-kinase, catalytic, gamma polypeptide
234779	phospholipase C, gamma 2
19057	protein phosphatase 3, catalytic subunit, gamma isoform

All genes shown featured ≥2-fold differential transcription and p<0.05.

**Table 7:** Pathways analysis reveals differential transcription of multiple gene members of oncogene-related pathways five weeks post-egg exposure.

ENTREZ GENE ID	GENE NAME
12143	B lymphoid kinase
66813	BCL2-like 14 (apoptosis facilitator)
14281	FBJ osteosarcoma oncogene
14191	Gardner-Rasheed feline sarcoma viral (Fgr) oncogene homolog
16909	LIM domain only 2
14159	feline sarcoma oncogene
17095	lymphoblastomic leukemia 1
16818	lymphocyte protein tyrosine kinase
20423	sonic hedgehog
22324	vav 1 oncogene

All genes shown feature ≥2-fold differential transcription and p<0.05.

**Table 8:** Pathways analysis reveals differential transcription of multiple gene members of mammary carcinogenesis-related pathways five weeks post-egg exposure.

ENTREZ GENE ID	GENE NAME
23960	2′-5′ oligoadenylate synthetase 1G
53313	ATPase, Ca++ transporting, ubiquitous
140703	EMI domain containing 1
246256	Fc receptor, IgG, low affinity IV
18194	NAD(P) dependent steroid dehydrogenase-like
18104	NAD(P)H dehydrogenase, quinone 1
229003	cDNA sequence BC006779
13040	cathepsin S
58187	claudin 10
12262	complement component 1, q subcomponent, C chain
93726	eosinophil-associated, ribonuclease A family, member 11
235439	hect domain and RCC1 (CHC1)-like domain (RLD) 1
14960, 14968	histocompatibility 2, class II antigen A/E alpha
14998	histocompatibility 2, class II, locus DMa
14999	histocompatibility 2, class II, locus Mb1
380794	Ig heavy chain 3 (serum IgG2b); Ig heavy chain (gamma polypeptide)
15894	intercellular adhesion molecule 1
68713	interferon induced transmembrane protein 1
107321	leupaxin
109225	membrane-spanning 4-domains, subfamily A, member 7
17969	neutrophil cytosolic factor 1
545007, 545013, 100040671	alpha7-takusan
16061, 100048770	immunoglobulin heavy chain (J558 family)
19283	protein tyrosine phosphatase, receptor type Z, polypeptide 1
20345	selectin, platelet (p-selectin) ligand
19261	signal-regulatory protein alpha
100044683	Leucine rich repeat containing 8 family, member E
100047788	similar to gamma-2a immunoglobulin heavy chain
100047619	similar to solute carrier family 7 (y+ system), member 5
21753	testis derived transcript
17230	tryptase alpha/beta 1

All genes shown feature ≥2-fold differential transcription and p<0.05.

### *S. haematobium* eggs initiate differential transcription of extracellular matrix-associated genes in the bladder

**Figure 5:**
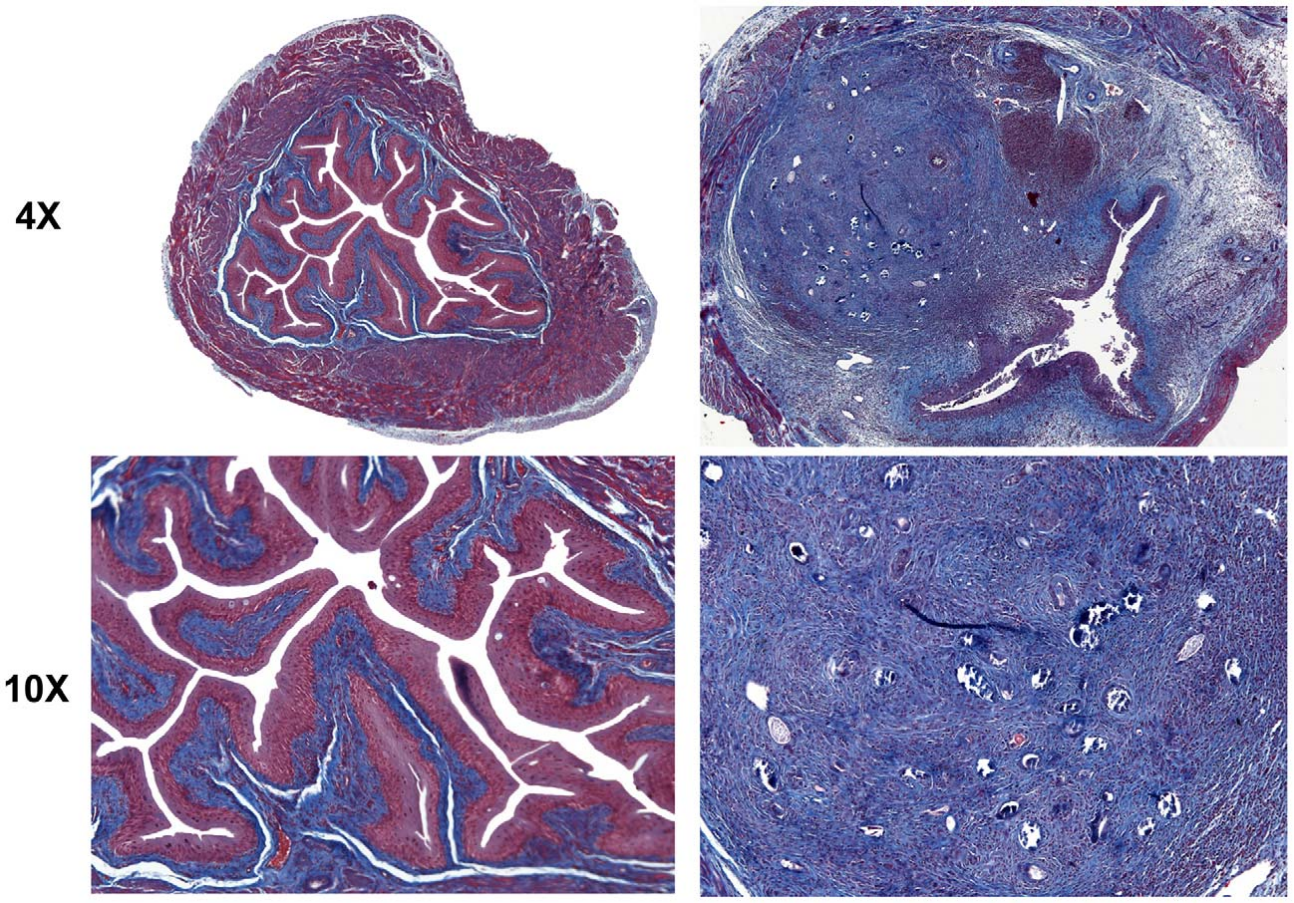
*S. haematobium* egg-injected bladders develop fibrosis. Egg-injected bladders demonstrate histologically-apparent fibrosis within granulomata (right column, sections from week 3 post-egg injection bladder, Masson's Trichrome stain, collagen stains blue; left column, sections from week 3 post-control vehicle injection bladder show no granuloma and normal collagen staining pattern).

The presence of *S. haematobium* eggs in the bladder prompted complex patterns of differential transcription of multiple collagen and metalloproteinase genes over time (Table 9). We confirmed through Masson's Trichrome staining that transcription of these extracellular matrix-associated genes was temporally associated with bladder tissue remodeling and fibrosis (Figure 5).

**Table 9:** Extracellular matrix-related genes featuring altered transcription by microarray analysis.

Gene	Fold-Regulation Post-Injection Relative to Control*			
	Time Post-Injection	Week 1	Week 3	Week 5
COL3A1		**	0.302919	0.258949
COL4A5		**	0.437578	**
COL6A3		**	0.448199	**
COL8A2		**	0.457323	**
COL17A1		**	3.59083	3.392723
COL7A1		**	2.00841	2.16737
TIMP1		6.01549	7.713105	3.41333
MMP10		8.79182	18.2758	11.5372
MMP13		4.98769	16.2878	5.75328
MMP3		2.21056	3.203963	3.084574
MMP9		**	3.90035	**

* All values shown ≥2-fold and p<0.05 unless otherwise noted.

** Value <2-fold and/or p≥0.05.

## Discussion

Although *S. haematobium* infection is one of the most important causes of helminth-related mortality worldwide, research on this important parasite has suffered due to a lack of high fidelity animal models. We recently demonstrated that direct injection of *S. haematobium* eggs into the bladder walls of mice recapitulates many features of human urogenital schistosomiasis, including granulomatous inflammation, urothelial hyperplasia, egg shedding, and bladder fibrosis [11]. Since oviposition is induced at a precisely known time point, our synchronous granuloma model is ideal for dissecting the initial biological responses that occur in the bladder after egg exposure. We applied gene microarray approaches to our mouse model in order to interrogate the early molecular events associated with the bladder sequelae of urogenital schistosomiasis. This first-ever microarray analysis revealed complex modulation of multiple genes, with a peak occurring 3 weeks after egg exposure. As expected, type 2 inflammation- and macrophage function-associated gene transcription was increased. Extracellular matrix remodeling-related gene transcription was differentially modulated over time. Pathways analysis pointed to differential transcription of multiple genes implicated in carcinogenesis. Surprisingly, microarray analysis uncovered decreased transcription of certain tight junction and all uroplakin genes, which occurred despite urothelial hyperplasia.

Clearly, *S. haematobium* eggs induced a complex bladder gene response that waxed and waned during the time course examined. Few genes were differentially transcribed one week after bladder injection with *S. haematobium* eggs. By three weeks after egg injection of the bladder, the numbers of differentially transcribed genes had peaked. For example, by five weeks post-egg injection, the pool of differentially transcribed genes was already contracting. This suggests that the chronic bladder changes seen in urogenital schistosomiasis cannot be sustained by a single set of eggs; rather, it is driven by continuous oviposition by adult worms. In this model, successive waves of oviposition, rather than any lone egg bolus (such as that featured in our model), would sustain a long-term bladder response. This is consistent with observations that early stage schistosomal urinary tract pathology eventually resolves after praziquantel therapy-induced worm death (which leads to cessation of oviposition) [15]. Regardless, our single bolus model of synchronous granuloma formation proved valuable for characterizing the earliest molecular events in the bladder that occur in response to exposure to *S. haematobium* eggs.

As expected, many of the early molecular events in the bladder were related to granulomatous type 2 inflammation. Schistosome eggs are potent inducers of granuloma formation in various tissues, including the intestines, liver, lung, and bladder. These granulomata feature various leukocyte subsets, including eosinophils, neutrophils, lymphocytes, macrophages, and epithelioid cells (activated macrophages). Pathways analysis suggested a role for B cells, given that a number of relevant genes were differentially transcribed. We suspect that many of these genes promote immunoglobulin functions, including those involved with IgE, the isotype most commonly associated with helminth infection (reviewed by Erb [16]). Accordingly, our mouse model features IgE production [11]. Granuloma formation in mouse models of *S. mansoni* and *Schistosoma japonicum* infection (i.e., hepatoenteric schistosomiasis) is associated with a local and systemic type 2 inflammatory response [17], [18]. This immune polarization typically features elevated levels of IL-4 and IL-13 which trigger alternative activation of macrophages. These macrophages selectively metabolize arginine through arginase-1, whereas their classically activated counterparts convert arginine to nitric oxide through nitric oxide synthase [19]–[21]. In our mouse model of urogenital schistosomiasis we have demonstrated that bladder granuloma formation is likewise associated with mixed leukocyte infiltration and regional and systemic production of type 2 cytokines [11]. Herein we have extended these findings by confirming granulomatous inflammation at additional time points and enhanced gene transcription for several chemokines, IL-4, the alternatively activated macrophage markers arginase-1, mannose receptor, and Ym-1/CHI3L3, and other indicators of type 2 inflammation. Although IL-13 gene transcription was unchanged, we have previously documented elevated protein levels of this cytokine from 1 through 4 weeks post-egg injection [11]. IL-13 has been implicated as a major mediator of fibrosis associated with *S. mansoni* egg exposure [22]–[29]. The pro-fibrogenic role of IL-13 is kept in check by the decoy receptor IL-13 receptor alpha 2 [28], [30]–[33]. Indeed, transcription of the IL-13 receptor alpha 2 gene was increased in our model, suggesting a possible role in negative feedback on IL-13-mediated fibrosis. Other genes with dampening influences over *S. mansoni*-induced inflammation and fibrosis include RELM-alpha/Fizz1 34,35, IL-10 [32], [36]–[38], and arginase [39]. Of these three mediators, only arginase featured increased transcription (although IL-10 receptor alpha, but not IL-10, also demonstrated enhanced transcription). Thus, we hypothesize that arginase may have a role in resolving bladder fibrosis. Additional studies will be necessary to clarify this issue. Finally, the observed protein expression of arginase-1 and lack of increased iNOS transcription is consistent with selective polarization of macrophages along the alternative activation program.

Another relevant issue is whether the immune and fibrosis responses to *S. haematobium* eggs in the bladder differ from those directed against *S. mansoni* eggs in other tissues. Perhaps the most appropriate comparisons can be made between our data and microarray analyses that have employed the *S. mansoni* egg-induced, synchronous lung granuloma model [40], [41]. These studies are methodologically analogous to this study's microarray analysis of our synchronous egg injection model. Numerous immune and fibrosis response genes feature increased transcription in both the *S. mansoni* and *S. haematobium* models. These genes include those encoding for CCL4 (MIP-1β), IL-4 induced 1, IL-6, cytokine inducible SH2-containing protein (CISH), C1q, IgG Fc receptors, eosinophil ribonucleases, arginase, arachidonate 15-lipoxygenase (ALOX15), platelet thromboxane A synthase 1 (TBXAS1), MMP9 and MMP13. Nonetheless, several notable genes showing elevated transcription in the *S. mansoni* studies did not feature differential transcription in our datasets, namely MCP1 (CCL2), CCR9, MCP2 (CCL8), and MMP12. The transcription patterns for MMP9, MMP12, and MMP13 may reflect distinct collagen-remodeling pathways in *S. mansoni* lung versus *S. haematobium* bladder fibrosis. Surprisingly, some genes with increased transcription in Th1-skewed mouse strains from the *S. mansoni* studies were also increased in our microarray analysis, which was based on the Th2-skewed BALB/c strain. These genes include IL-1β, interferon gamma-inducible proteins, CCL5 (RANTES), and macrophage-expressed gene 1 (MPEG1). Hence, our model seems to feature increased transcription of a greater mix of type 1 and 2 immune response-associated genes as compared to the *S. mansoni* studies. We conclude that although the *S. haematobium* egg-directed immune and fibrotic response in the bladder shares many similarities to the *S. mansoni* egg-triggered lung response, there are a number of potentially important disparities. There is a precedent in the literature for schistosome- and tissue-specific immune and fibrotic responses. Liver- and lung-associated, *S. mansoni* egg granulomata develop in a highly organ-specific fashion [42]. *S. japonicum* granulomata also evolve in a tissue-specific manner in the liver, lung, and intestinal tract [43]. These reports highlight the critical need to develop *in vivo* models which properly match schistosome species with their tropism for specific host organs.

Besides validating our prior immunologic- and fibrosis-related observations, microarray analysis also identified complex modulation of genes integral to urothelial function. Uroplakins are key structural proteins that form organized plaques on the surface of urothelial cells. The coordinated expression of the various uroplakin genes is believed to confer both impermeability and flexibility to the urothelium. These two functions are critical to the bladder's ability to safely sequester accumulating urine and expel it during micturition. Accordingly, mice deficient for various uroplakins exhibit bladder dysfunction and increased urothelial permeability [44], [45]. We were intrigued by the finding that the transcription of all uroplakin genes was dampened at three weeks after *S. haematobium* egg introduction to the bladder. Other workers have reported less uroplakin gene transcription and urothelial hyperplasia after administration of disparate noxious stimuli to the bladder, including Bacillus Calmette-Guerín (BCG) [46], cyclophosphamide [47]–[49], and an *in vitro* model of culture media-induced urothelial hyperplasia [50]. Urothelial hyperplasia in this setting is a secondary response to reseal urothelial defects that have resulted from desquamation of dead urothelial cells [51], [52]. Strikingly, this urothelial response appears to be conserved across species, given that these studies have encompassed mice, rats, and pigs. To our knowledge, we report here the first characterization of uroplakin changes triggered by urogenital schistosomiasis. In our past work we have identified the onset of urothelial hyperplasia after *S. haematobium* egg injection [11], and herein have corroborated this at other time points. Taken together, this suggests that decreased uroplakin gene transcription and urothelial hyperplasia are coupled processes that are part of a conserved bladder response to a range of forms of urothelial injury. In our model, decreased transcription of uroplakin genes and urothelial hyperplasia occurs in the setting of egg shedding in urine. We speculate that *S. haematobium* eggs induce, or at least exploit, the compromised urothelial barrier (i.e., decreased uroplakins) to pass into urine. These possibilities highlight the complex dynamics of urothelial biology in urogenital schistosomiasis. Our findings also underscore the importance of high fidelity urogenital schistosomiasis models. Namely, extrapolation of *S. haematobium* urogenital disease mechanisms from *S. mansoni* hepatoenteric disease models is not possible, given the tissue-specific expression of uroplakins.

Loss of uroplakin expression has been noted in some urothelial cancers [53], [54]. Although it is tempting to causally link these observations to our findings and schistosomal bladder cancer, the lack of reports of development of bladder cancer in uroplakin-deficient mice suggests that loss of uroplakin expression may not be carcinogenic. Conversely, we identified differential transcription of genes implicated in multiple carcinogenesis pathways, including vascular endothelial growth factor (VEGF)-, oncogene-, and mammary tumor-related genes. Tissue, plasma, and urine levels of VEGF have been reported to be elevated in patients with schistosomal bladder cancer [55]. Our past work is consistent with these findings in that bladder VEGF increases after egg injection [11]. We speculate that elevated VEGF in the bladder may promote cancer progression by stimulating tumor vasculogenesis. In addition, we also conjecture that VEGF mediates growth of abnormal, friable blood vessels which rupture and leak blood through the uroplakin-poor, compromised urothelium, ultimately resulting in the hematuria associated with urogenital schistosomiasis.

Additional evidence for a compromised urothelial barrier was identified in the form of dampened transcription of tight junction-related genes. While uroplakins contribute to the urothelium's water and urea permeability barrier, tight junctions have a complementary role. These structures confer transmembrane epithelial resistance to the urothelium [56]. We found that gene transcription of claudin-8 and junctional adhesion molecule-4, components of tight junctions in the bladder urothelium [50], [57]–[59], was lessened after egg injection. Bladder urothelial tight junction expression of claudins and junctional adhesion molecules is found in rats, mice, rabbits, pigs, and humans, which highlights the tight conservation of these genes across mammalian species and hints at their biological importance.

Bladder fibrosis, disorganized deposition of extracellular matrix in the bladder wall, is another critical biological process associated with urogenital schistosomiasis. We have previously reported induction of bladder fibrosis after *S. haematobium* egg injection that resembles human disease [11]. Here, we also histologically confirmed fibrosis at additional time points that correlated with fibrosis-related gene transcription. Specifically, we identified augmented transcription of the collagen genes COL7A1 and COL17A1, metalloproteinases-3, -9, -10, and -13, and tissue inhibitor of metalloproteinase 1 (TIMP1). Interestingly, transcription of other collagen genes, i.e., COL3A1, COL4A5, COL6A3, and COL8A2, were decreased after egg injection. Normal levels of COL3A1 have been shown through transgenic mouse studies to be important for proper bladder function [60]. In addition, MMP9, MMP13, and TIMP1 are expressed in many bladder cancers and may mediate tumor invasiveness through extracellular matrix regulation [61], [62]. The differential transcription of numerous genes linked to extracellular matrix remodeling underscores the intricate fibrosis- and cancer-promoting pathways associated with schistosomal granuloma formation.

Although our findings are highly informative, the employed mouse model features limitations. Since *Mus musculus* and *S. haematobium* are both eukaryotes, theoretically speaking these species may feature homologous genes. However, we believe that the probability of orthologs sharing significant nucleotide homology is quite low. Codon usage in mice and trematodes such as *S. haematobium* is different, as exemplified by the need for codon optimization to maximize expression of *Schistosoma* genes by mammalian cells [63], [64]. Moreover, we only injected 3000 eggs per mouse bladder, and eggs are shed in the urine over time. Hence, *S. haematobium* RNA is only a small fraction of the total RNA in egg-injected mouse bladder tissue, making it unlikely that *S. haematobium* cRNA hybridization to microarray chips (if any) significantly affected our analysis. Another limitation is that our egg injection model does not reproduce the entire *S. haematobium* life cycle of the human host. Cercariae are found in the skin and subcutaneous tissues, schistosomula circulate in the systemic and portal circulation, and adult worms reside in the pelvic venous plexus. However, for the purposes of this bladder-focused study, eggs are sufficient since it is the only *S. haematobium* life stage present in bladder tissue. It is also possible that *S. haematobium* eggs transferred from hamsters (the source of eggs in our model) to a final mouse host may be metabolically and immunologically distinct from eggs laid *in situ* in a single mouse host. This has been postulated to occur with *S. japonicum* eggs [43]. We have controlled for this in part by using control injections of hamster liver and intestine-derived “vehicle”. However, studies are underway to compare *in vitro* laid eggs to hamster-derived eggs in our mouse model. The single, large egg bolus utilized in our model is unlikely to reflect the kinetics of continuous oviposition in the human host. On the other hand, our model features highly focal, coalescing multiple egg-based granulomata that are reminiscent of those observed in the human bladder [65]. Regardless of these issues, our model mitigates the relative scarcity of early disease stage bladder tissue available for research. Bladder tissue from schistosomiasis patients is typically only available from those undergoing cystectomy or bladder reconstructive surgery for advanced bladder cancer or fibrosis, respectively. Thus, our model fills an important niche in basic research on urogenital schistosomiasis.

In conclusion, we have defined for the first time the initial molecular underpinnings of the bladder response to *S. haematobium* eggs in an experimental model of urogenital schistosomiasis. Although this response is manifold, it is discrete, involves known inflammatory, fibrosis, epithelial, and cancer-related pathways, and expands and contracts over time. This work may direct future efforts to develop diagnostic and therapeutic tools for the bladder sequelae of urogenital schistosomiasis and potentially cancers and inflammatory disorders of the bladder in general.

## Supporting Information

Table S1 **Functional annotation clustering of differentially transcribed genes in egg-injected mice.** All genes shown featured ≥2-fold differential transcription and p<0.05.
(DOCX)

Table S2 **Illumina probe IDs and Entrez Gene IDs of differentially transcribed genes.** All genes shown featured ≥2-fold differential transcription and p<0.05. Entrez Gene IDs and descriptions were not available for some genes.
(XLSX)

Table S3 **Primers used for real-time PCR validation of microarray data.** Primer sequences were derived from PrimerBank (http://pga.mgh.harvard.edu/primerbank/).
(XLSX)

## References

[1] King CH (2010) Health metrics for helminthic infections. Adv Parasitol 73: 51–69 Available: http://www.ncbi.nlm.nih.gov/entrez/query.fcgi?cmd=Retrieve&db=PubMed&dopt=Citation&list_uids=20627139. Accessed 2012 Oct 30. 20627139 10.1016/S0065-308X(10)73003-7

[2] van der Werf MJ, de Vlas SJ, Brooker S, Looman CW, Nagelkerke NJ, et al (2003) Quantification of clinical morbidity associated with schistosome infection in sub-Saharan Africa. Acta Trop 86: 125–139 Available: http://www.ncbi.nlm.nih.gov/entrez/query.fcgi?cmd=Retrieve&db=PubMed&dopt=Citation&list_uids=12745133. Accessed 2011 Aug 18. 12745133 10.1016/s0001-706x(03)00029-9

[3] Group W (2011) Schistosoma haematobium. In: International Agency for Research on Cancer WHO, editor. A Review of Human Carcinogens: Biological Agents. Geneva: World Health Organization, Vol. 100B. pp. 377–390.

[4] Bedwani R, Renganathan E, El Kwhsky F, Braga C, Abu Seif HH, et al (1998) Schistosomiasis and the risk of bladder cancer in Alexandria, Egypt. British journal of cancer 77: 1186–1189 Available: http://www.pubmedcentral.nih.gov/articlerender.fcgi?artid=2150141&tool=pmcentrez&rendertype=abstract. Accessed 2012 Oct 30. 9569060 10.1038/bjc.1998.197 PMC2150141

[5] Rollinson D (2009) A wake up call for urinary schistosomiasis: reconciling research effort with public health importance. Parasitology 136: 1593–1610 Available: http://www.ncbi.nlm.nih.gov/pubmed/19627633. Accessed 2011 Dec 24. 19627633 10.1017/S0031182009990552

[6] Loker ES (1983) A comparative study of the life-histories of mammalian schistosomes. Parasitology 87 ((Pt 2)) 343–369 Available: http://www.ncbi.nlm.nih.gov/entrez/query.fcgi?cmd=Retrieve&db=PubMed&dopt=Citation&list_uids=6359028. Accessed 2012 Oct 30. 6359028 10.1017/s0031182000052689

[7] Rheinberg CE, Mone H, Caffrey CR, Imbert-Establet D, Jourdane J, et al (1998) Schistosoma haematobium, S. intercalatum, S. japonicum, S. mansoni, and S. rodhaini in mice: relationship between patterns of lung migration by schistosomula and perfusion recovery of adult worms. Parasitol Res 84: 338–342 Available: http://www.ncbi.nlm.nih.gov/entrez/query.fcgi?cmd=Retrieve&db=PubMed&dopt=Citation&list_uids=9569102. Accessed 2012 Oct 30. 9569102 10.1007/s004360050407

[8] Kuntz RE, Malakatis GM (1955) Susceptibility studies in schistosomiasis. IV. Susceptibility of wild mammals to infection by Schistosoma haematobium in Egypt, with emphasis on rodents. J Parasitol 41: 467–475 Available: http://www.ncbi.nlm.nih.gov/entrez/query.fcgi?cmd=Retrieve&db=PubMed&dopt=Citation&list_uids=13264019. Accessed 2012 Oct 30. 13264019

[9] Vuong PN, Bayssade-Dufour C, Albaret JL, Farhati K (1996) Histopathological observations in new and classic models of experimental Schistosoma haematobium infections. Trop Med Int Health 1: 348–358 Available: http://www.ncbi.nlm.nih.gov/entrez/query.fcgi?cmd=Retrieve&db=PubMed&dopt=Citation&list_uids=8673838. Accessed 2012 Oct 30. 8673838 10.1046/j.1365-3156.1996.d01-52.x

[10] Ordan P, Goatly KD (1966) Experimental schistosomiasis in primates in Tanzania. I. A preliminary note on the susceptibility of Cercopithecus aethiops centralis to infection with Schistosoma haematobium and Schistosoma mansoni. Ann Trop Med Parasitol 60: 3–9 Available: http://www.ncbi.nlm.nih.gov/entrez/query.fcgi?cmd=Retrieve&db=PubMed&dopt=Citation&list_uids=4960090. Accessed 2012 Oct 30. 4960090

[11] Fu C-L, Odegaard JI, Herbert DR, Hsieh MH (2012) A Novel Mouse Model of Schistosoma haematobium Egg-Induced Immunopathology. PLoS Pathog 8: e1002605 Available: http://dx.plos.org/10.1371/journal.ppat.1002605. Accessed 2012 Mar 30. 22479181 10.1371/journal.ppat.1002605 PMC3315496

[12] Fu C-LL, Apelo CA, Torres B, Thai KH, Hsieh MH (2011) Mouse bladder wall injection. Journal of visualized experiments JoVE Available: http://www.ncbi.nlm.nih.gov/entrez/query.fcgi?cmd=Retrieve&db=PubMed&dopt=Citation&list_uids=21775962. Accessed 2012 Oct 30. 10.3791/2523 PMC3196188 21775962

[13] Dennis G, Sherman BT, Hosack DA, Yang J, Gao W, et al (2003) DAVID: Database for Annotation, Visualization, and Integrated Discovery. Genome Biology 4: R60 Available: http://www.ncbi.nlm.nih.gov/pubmed/12734009. Accessed 2012 Oct 30. PMC193660 12734009

[14] Huang DW, Sherman BT, Lempicki RA (2008) Systematic and integrative analysis of large gene lists using DAVID bioinformatics resources. Nat Protocols 4: 44–57 Available: http://www.ncbi.nlm.nih.gov/pubmed/19131956. Accessed 2012 Oct 30. 10.1038/nprot.2008.211 19131956

[15] Richter J, Poggensee G, Kjetland EF, Helling-Giese G, Chitsulo L, et al.. (1996) Reversibility of lower reproductive tract abnormalities in women with Schistosoma haematobium infection after treatment with praziquantel–an interim report. 10.1016/s0001-706x(96)00030-7 9028413

[16] Erb KJ (2007) Helminths, allergic disorders and IgE-mediated immune responses: where do we stand? European journal of immunology 37: 1170–1173 Available: http://www.ncbi.nlm.nih.gov/pubmed/17447233. Accessed 2012 Sep 10. 17447233 10.1002/eji.200737314

[17] Xu YH, Macedonia J, Sher A, Pearce E, Cheever AW (1991) Dynamic analysis of splenic Th1 and Th2 lymphocyte functions in mice infected with Schistosoma japonicum. Infection and Immunity 59: 2934–2940. 1679041 10.1128/iai.59.9.2934-2940.1991 PMC258116

[18] Hewitson JP, Grainger JR, Maizels RM (2009) Helminth immunoregulation: the role of parasite secreted proteins in modulating host immunity. Molecular and biochemical parasitology 167: 1–11 doi:10.1016/j.molbiopara.2009.04.008. 19406170 10.1016/j.molbiopara.2009.04.008 PMC2706953

[19] Munder M, Eichmann K, Moran JM, Centeno F, Soler G, et al (1999) Th1/Th2-regulated expression of arginase isoforms in murine macrophages and dendritic cells. J Immunol 163: 3771–3777 Available: http://www.ncbi.nlm.nih.gov/entrez/query.fcgi?cmd=Retrieve&db=PubMed&dopt=Citation&list_uids=10490974. Accessed 2012 Oct 30. 10490974

[20] Ellyard JI, Quah BJ, Simson L, Parish CR (2010) Alternatively activated macrophage possess antitumor cytotoxicity that is induced by IL-4 and mediated by arginase-1. J Immunother 33: 443–452 Available: http://www.ncbi.nlm.nih.gov/entrez/query.fcgi?cmd=Retrieve&db=PubMed&dopt=Citation&list_uids=20463604. Accessed 2012 Oct 30. 20463604 10.1097/CJI.0b013e3181cd8746

[21] Modolell M, Corraliza IM, Link F, Soler G, Eichmann K (1995) Reciprocal regulation of the nitric oxide synthase/arginase balance in mouse bone marrow-derived macrophages by TH1 and TH2 cytokines. Eur J Immunol 25: 1101–1104 Available: http://www.ncbi.nlm.nih.gov/entrez/query.fcgi?cmd=Retrieve&db=PubMed&dopt=Citation&list_uids=7537672. Accessed 2012 Oct 30. 7537672 10.1002/eji.1830250436

[22] Chiaramonte MG, Cheever AW, Malley JD, Donaldson DD, Wynn TA (2001) Studies of murine schistosomiasis reveal interleukin-13 blockade as a treatment for established and progressive liver fibrosis. Hepatology (Baltimore, Md) 34: 273–282 Available: http://www.ncbi.nlm.nih.gov/pubmed/11481612. Accessed 2012 Sep 9. 10.1053/jhep.2001.26376 11481612

[23] Chiaramonte MG, Donaldson DD, Cheever AW, Wynn TA (1999) An IL-13 inhibitor blocks the development of hepatic fibrosis during a T-helper type 2-dominated inflammatory response. The Journal of clinical investigation 104: 777–785 Available: http://www.pubmedcentral.nih.gov/articlerender.fcgi?artid=408441&tool=pmcentrez&rendertype=abstract. Accessed 2012 Sep 9. 10491413 10.1172/JCI7325 PMC408441

[24] de Jesus AR, Magalhães A, Miranda DG, Miranda RG, Araújo MI, et al (2004) Association of type 2 cytokines with hepatic fibrosis in human Schistosoma mansoni infection. Infection and immunity 72: 3391–3397 Available: http://www.pubmedcentral.nih.gov/articlerender.fcgi?artid=415716&tool=pmcentrez&rendertype=abstract. Accessed 2012 Sep 9. 15155645 10.1128/IAI.72.6.3391-3397.2004 PMC415716

[25] Fallon PG, Richardson EJ, McKenzie GJ, McKenzie AN (2000) Schistosome infection of transgenic mice defines distinct and contrasting pathogenic roles for IL-4 and IL-13: IL-13 is a profibrotic agent. Journal of immunology (Baltimore, Md: 1950) 164: 2585–2591 Available: http://www.ncbi.nlm.nih.gov/pubmed/10679097. Accessed 2012 Sep 9. 10.4049/jimmunol.164.5.2585 10679097

[26] Jakubzick C, Choi ES, Kunkel SL, Joshi BH, Puri RK, et al (2003) Impact of interleukin-13 responsiveness on the synthetic and proliferative properties of Th1- and Th2-type pulmonary granuloma fibroblasts. The American journal of pathology 162: 1475–1486 Available: http://www.pubmedcentral.nih.gov/articlerender.fcgi?artid=1851205&tool=pmcentrez&rendertype=abstract. Accessed 2012 Sep 9. 12707030 10.1016/S0002-9440(10)64280-0 PMC1851205

[27] Kaviratne M, Hesse M, Leusink M, Cheever AW, Davies SJ, et al (2004) IL-13 activates a mechanism of tissue fibrosis that is completely TGF-beta independent. Journal of immunology (Baltimore, Md: 1950) 173: 4020–4029 Available: http://www.ncbi.nlm.nih.gov/pubmed/15356151. Accessed 2012 Sep 9. 10.4049/jimmunol.173.6.4020 15356151

[28] Madala SK, Pesce JT, Ramalingam TR, Wilson MS, Minnicozzi S, et al (2010) Matrix metalloproteinase 12-deficiency augments extracellular matrix degrading metalloproteinases and attenuates IL-13-dependent fibrosis. Journal of immunology (Baltimore, Md: 1950) 184: 3955–3963 Available: http://www.pubmedcentral.nih.gov/articlerender.fcgi?artid=3175622&tool=pmcentrez&rendertype=abstract. Accessed 2012 Jul 20. 10.4049/jimmunol.0903008 PMC3175622 20181883

[29] Singh KP, Gerard HC, Hudson AP, Boros DL (2004) Dynamics of collagen, MMP and TIMP gene expression during the granulomatous, fibrotic process induced by Schistosoma mansoni eggs. Annals of tropical medicine and parasitology 98: 581–593 Available: http://www.ncbi.nlm.nih.gov/pubmed/15324465. Accessed 2012 Jul 25. 15324465 10.1179/000349804225021316

[30] Chiaramonte MG, Mentink-Kane M, Jacobson BA, Cheever AW, Whitters MJ, et al (2003) Regulation and function of the interleukin 13 receptor alpha 2 during a T helper cell type 2-dominant immune response. The Journal of experimental medicine 197: 687–701 Available: http://www.pubmedcentral.nih.gov/articlerender.fcgi?artid=2193852&tool=pmcentrez&rendertype=abstract. Accessed 2012 Sep 9. 12642601 10.1084/jem.20020903 PMC2193852

[31] Mentink-Kane MM, Cheever AW, Thompson RW, Hari DM, Kabatereine NB, et al (2004) IL-13 receptor alpha 2 down-modulates granulomatous inflammation and prolongs host survival in schistosomiasis. Proceedings of the National Academy of Sciences of the United States of America 101: 586–590 Available: http://www.pubmedcentral.nih.gov/articlerender.fcgi?artid=327191&tool=pmcentrez&rendertype=abstract. Accessed 2012 Sep 9. 14699044 10.1073/pnas.0305064101 PMC327191

[32] Mentink-Kane MM, Cheever AW, Wilson MS, Madala SK, Beers LM, et al (2011) Accelerated and progressive and lethal liver fibrosis in mice that lack interleukin (IL)-10, IL-12p40, and IL-13Rα2. Gastroenterology 141: 2200–2209 Available: http://www.ncbi.nlm.nih.gov/pubmed/21864478. Accessed 2012 Sep 9. 21864478 10.1053/j.gastro.2011.08.008 PMC3221932

[33] Wynn TA, Hesse M, Sandler NG, Kaviratne M, Hoffmann KF, et al (2004) P-selectin suppresses hepatic inflammation and fibrosis in mice by regulating interferon gamma and the IL-13 decoy receptor. Hepatology (Baltimore, Md) 39: 676–687 Available: http://www.ncbi.nlm.nih.gov/pubmed/14999686. Accessed 2012 Sep 9. 10.1002/hep.20102 14999686

[34] Pesce JT, Ramalingam TR, Wilson MS, Mentink-Kane MM, Thompson RW, et al (2009) Retnla (relmalpha/fizz1) suppresses helminth-induced Th2-type immunity. PLoS Pathog 5: e1000393 Available: http://www.ncbi.nlm.nih.gov/entrez/query.fcgi?cmd=Retrieve&db=PubMed&dopt=Citation&list_uids=19381262. Accessed 2012 Sep 9. 19381262 10.1371/journal.ppat.1000393 PMC2663845

[35] Nair MG, Du Y, Perrigoue JG, Zaph C, Taylor JJ, et al (2009) Alternatively activated macrophage-derived RELM-{alpha} is a negative regulator of type 2 inflammation in the lung. The Journal of experimental medicine 206: 937–952 Available: http://www.pubmedcentral.nih.gov/articlerender.fcgi?artid=2715126&tool=pmcentrez&rendertype=abstract. Accessed 2012 Aug 22. 19349464 10.1084/jem.20082048 PMC2715126

[36] Fairfax KC, Amiel E, King IL, Freitas TC, Mohrs M, et al (2012) IL-10R blockade during chronic schistosomiasis mansoni results in the loss of B cells from the liver and the development of severe pulmonary disease. PLoS pathogens 8: e1002490 Available: http://www.pubmedcentral.nih.gov/articlerender.fcgi?artid=3266936&tool=pmcentrez&rendertype=abstract. Accessed 2012 Sep 3. 22291593 10.1371/journal.ppat.1002490 PMC3266936

[37] Booth M, Mwatha JK, Joseph S, Jones FM, Kadzo H, et al (2004) Periportal fibrosis in human Schistosoma mansoni infection is associated with low IL-10, low IFN-gamma, high TNF-alpha, or low RANTES, depending on age and gender. Journal of immunology (Baltimore, Md: 1950) 172: 1295–1303 Available: http://www.ncbi.nlm.nih.gov/pubmed/14707108. Accessed 2012 Sep 9. 10.4049/jimmunol.172.2.1295 14707108

[38] Wynn TA, Cheever AW, Williams ME, Hieny S, Caspar P, et al (1998) IL-10 regulates liver pathology in acute murine Schistosomiasis mansoni but is not required for immune down-modulation of chronic disease. Journal of immunology (Baltimore, Md: 1950) 160: 4473–4480 Available: http://www.ncbi.nlm.nih.gov/pubmed/9574553. Accessed 2012 Sep 9. 9574553

[39] Pesce JT, Ramalingam TR, Mentink-Kane MM, Wilson MS, El Kasmi KC, et al (2009) Arginase-1–Expressing Macrophages Suppress Th2 Cytokine–Driven Inflammation and Fibrosis. PLoS Pathog 5: e1000371 Available: http://www.ncbi.nlm.nih.gov/entrez/query.fcgi?cmd=Retrieve&db=PubMed&dopt=Citation&list_uids=19360123. Accessed 2011 Jun 15. 19360123 10.1371/journal.ppat.1000371 PMC2660425

[40] Global Gene Expression Profiles During Acute Pathogen-Induced Pulmonary Inflammation Reveal Divergent Roles for Th1 and Th2 Responses in Tissue Repair (n.d.). Available: http://www.jimmunol.org.laneproxy.stanford.edu/content/171/7/3655/T2.expansion.html. Accessed 2012 Sep 9. 10.4049/jimmunol.171.7.3655 14500663

[41] Wilson MS, Madala SK, Ramalingam TR, Gochuico BR, Rosas IO, et al (2010) Bleomycin and IL-1beta-mediated pulmonary fibrosis is IL-17A dependent. J Exp Med 207: 535–552 Available: http://www.ncbi.nlm.nih.gov/entrez/query.fcgi?cmd=Retrieve&db=PubMed&dopt=Citation&list_uids=20176803. Accessed 2012 Oct 30. 20176803 10.1084/jem.20092121 PMC2839145

[42] Edungbola LD, Schiller EL (1979) Histopathology of hepatic and pulmonary granulomata experimentally induced with eggs of Schistosoma mansoni. J Parasitol 65: 253–261 Available: http://www.ncbi.nlm.nih.gov/entrez/query.fcgi?cmd=Retrieve&db=PubMed&dopt=Citation&list_uids=571911. Accessed 2012 Oct 30. 571911

[43] Hirata M, Takushima M, Kage M, Fukuma T (1993) Comparative analysis of hepatic, pulmonary, and intestinal granuloma formation around freshly laid Schistosoma japonicum eggs in mice. Parasitol Res 79: 316–321 Available: http://www.ncbi.nlm.nih.gov/entrez/query.fcgi?cmd=Retrieve&db=PubMed&dopt=Citation&list_uids=8327455. Accessed 2012 Oct 30. 8327455 10.1007/BF00932188

[44] Hu P, Deng F-M, Liang F-X, Hu C-M, Auerbach AB, et al (2000) Ablation of uroplakin III gene results in small urothelial plaques, urothelial leakage, and vesicoureteral reflux. The Journal of Cell Biology 151: 961–972 Available: http://www.ncbi.nlm.nih.gov/pubmed/11378094. Accessed 2012 Oct 30. 11085999 10.1083/jcb.151.5.961 PMC2174354

[45] Aboushwareb T, Zhou G, Deng F-M, Turner C, Andersson K-E, et al (2009) Alterations in bladder function associated with urothelial defects in uroplakin II and IIIa knockout mice. Neurourology and Urodynamics 28: 1028–1033 Available: http://www.ncbi.nlm.nih.gov/entrez/query.fcgi?cmd=Retrieve&db=PubMed&dopt=Citation&list_uids=19267388. Accessed 2012 Oct 30. 19267388 10.1002/nau.20688 PMC4048927

[46] Saban MR, Hellmich HL, Simpson C, Davis CA, Lang ML, et al (2007) Repeated BCG treatment of mouse bladder selectively stimulates small GTPases and HLA antigens and inhibits single-spanning uroplakins. BMC Cancer 7: 204 Available: http://www.pubmedcentral.nih.gov/articlerender.fcgi?artid=2212656&tool=pmcentrez&rendertype=abstract. Accessed 2012 Oct 30. 17980030 10.1186/1471-2407-7-204 PMC2212656

[47] Kyung YS, Park HY, Lee G (2011) Preservation of uroplakins by 2-mercaptoethanesulfonate in cyclophosphamide-induced rat cystitis. Archives of Toxicology 85: 51–57. 20186394 10.1007/s00204-010-0523-y

[48] Choi SH, Byun Y, Lee G (2009) Expressions of Uroplakins in the Mouse Urinary Bladder with Cyclophosphamide-Induced Cystitis. Journal of Korean Medical Science 24: 684–689. 19654953 10.3346/jkms.2009.24.4.684 PMC2719198

[49] Romih R, Jezernik K (1996) Reorganisation of the urothelial luminal plasma membrane in the cyclophosphamide treated rats. Pflugers Archiv European journal of physiology 431: R241–R242. 8739354 10.1007/BF02346358

[50] Višnjar T, Kocbek P, Kreft ME (2011) Hyperplasia as a mechanism for rapid resealing urothelial injuries and maintaining high transepithelial resistance. Histochemistry and cell biology Available: http://www.ncbi.nlm.nih.gov/pubmed/22127649. Accessed 2012 Jan 15. 10.1007/s00418-011-0893-0 22127649

[51] Veranic P, Jezernik K (2000) The response of junctional complexes to induced desquamation in mouse bladder urothelium. Biology of the cell under the auspices of the European Cell Biology Organization 92: 105–113. 10.1016/s0248-4900(00)89018-8 10879631

[52] Veranic P, Erman A, Kerec-Kos M, Bogataj M, Mrhar A, et al (2009) Rapid differentiation of superficial urothelial cells after chitosan-induced desquamation. Histochemistry and Cell Biology 131: 129–139. 18797916 10.1007/s00418-008-0492-x

[53] Matsumoto K, Satoh T, Irie A, Ishii J, Kuwao S, et al (2008) Loss expression of uroplakin III is associated with clinicopathologic features of aggressive bladder cancer. Urology 72: 444–449 Available: http://www.ncbi.nlm.nih.gov/pubmed/18313120. Accessed 2012 Oct 30. 18313120 10.1016/j.urology.2007.11.128

[54] Olsburgh J, Harnden P, Weeks R, Smith B, Joyce A, et al (2003) Uroplakin gene expression in normal human tissues and locally advanced bladder cancer. Journal of Pathology The 199: 41–49 Available: http://dx.doi.org/10.1002/path.1252. Accessed 2012 Oct 30. 10.1002/path.1252 12474225

[55] Salem HK, Ragab H, Maksoud NAE (2012) Vascular endothelial growth factor expression in Schistosomiasis-associated bladder cancer, correlation with histopathological features and schistosomiasis. American Urological Association Annual Meeting

[56] Hu P, Meyers S, Liang F-X, Deng F-M, Kachar B, et al (2002) Role of membrane proteins in permeability barrier function: uroplakin ablation elevates urothelial permeability. American journal of physiology Renal physiology 283: F1200–F1207 Available: http://www.ncbi.nlm.nih.gov/pubmed/12388410. Accessed 2012 Oct 30. 12388410 10.1152/ajprenal.00043.2002

[57] Acharya P, Beckel J, Ruiz WG, Wang E, Rojas R, et al (2004) Distribution of the tight junction proteins ZO-1, occludin, and claudin-4, -8, and -12 in bladder epithelium. American journal of physiology Renal physiology 287: F305–F318 Available: http://www.ncbi.nlm.nih.gov/pubmed/15068973. Accessed 2012 Oct 30. 15068973 10.1152/ajprenal.00341.2003

[58] Khandelwal P, Abraham SN, Apodaca G (2009) Cell biology and physiology of the uroepithelium. American journal of physiology Renal physiology 297: F1477–501 Available: http://www.pubmedcentral.nih.gov/articlerender.fcgi?artid=2801337&tool=pmcentrez&rendertype=abstract. Accessed 2012 Jan 26. 19587142 10.1152/ajprenal.00327.2009 PMC2801337

[59] Sánchez Freire V, Burkhard FC, Schmitz A, Kessler TM, Monastyrskaya K (2011) Structural differences between the bladder dome and trigone revealed by mRNA expression analysis of cold-cut biopsies. BJU International 108: E126–E135 Available: http://www.ncbi.nlm.nih.gov/pubmed/21244608. Accessed 2012 Oct 30. 21244608 10.1111/j.1464-410X.2010.09934.x

[60] Stevenson K, Kucich U, Whitbeck C, Levin RM, Howard PS (2006) Functional changes in bladder tissue from type III collagen-deficient mice. Molecular and Cellular Biochemistry 283: 107–114 Available: http://www.ncbi.nlm.nih.gov/pubmed/16444592. Accessed 2012 Oct 30. 16444592 10.1007/s11010-006-2388-1

[61] Boström PJ, Ravanti L, Reunanen N, Aaltonen V, Söderström KO, et al (2000) Expression of collagenase-3 (matrix metalloproteinase-13) in transitional-cell carcinoma of the urinary bladder. International journal of cancer Journal international du cancer 88: 417–423. 11054671

[62] Staack A, Badendieck S, Schnorr D, Loening SA, Jung K (2006) Combined determination of plasma MMP2, MMP9, and TIMP1 improves the non-invasive detection of transitional cell carcinoma of the bladder. BMC Urology 6: 19 Available: http://www.pubmedcentral.nih.gov/articlerender.fcgi?artid=1560390&tool=pmcentrez&rendertype=abstract. Accessed 2012 Oct 30. 16901349 10.1186/1471-2490-6-19 PMC1560390

[63] Zhu Y, Lu F, Dai Y, Wang X, Tang J, et al (2010) Synergistic enhancement of immunogenicity and protection in mice against Schistosoma japonicum with codon optimization and electroporation delivery of SjTPI DNA vaccines. Vaccine 28: 5347–5355 Available:http://www.ncbi.nlm.nih.gov/pubmed/20483191. Accessed 2012 Sep 8. 20483191 10.1016/j.vaccine.2010.05.017

[64] Hamdan FF, Mousa A, Ribeiro P (2002) Codon optimization improves heterologous expression of a Schistosoma mansoni cDNA in HEK293 cells. Parasitology research 88: 583–586 Available: http://www.ncbi.nlm.nih.gov/pubmed/12107483. Accessed 2012 Sep 8. 12107483 10.1007/s00436-001-0585-0

[65] Cheever AW, Kamel IA, Elwi AM, Mosimann JE, Danner R, et al (1978) Schistosoma mansoni and S. haematobium infections in Egypt. III. Extrahepatic pathology. The American Journal of Tropical Medicine and Hygiene 27: 55–75 Available: http://www.ncbi.nlm.nih.gov/entrez/query.fcgi?cmd=Retrieve&db=PubMed&dopt=Citation&list_uids=626283. Accessed 2012 Oct 30. 626283 10.4269/ajtmh.1978.27.55

