# Supplementary material for: Transcriptional Profiling of the Bladder in Urogenital Schistosomiasis Reveals Pathways of Inflammatory Fibrosis and Urothelial Compromise
Source: PLoS Negl Trop Dis. 2012 Nov 29;6(11):e1912. doi: 10.1371/journal.pntd.0001912 (PMC3510078; doi:10.1371/journal.pntd.0001912)
Supplement: Table S1 — Functional annotation clustering of differentially transcribed genes in egg-injected mice. All genes shown featured ≥2-fold differential transcription and p<0.05. (DOCX) [file pntd.0001912.s001.docx]

**WEEK 1**

| Gene Symbol | Illumina Probe ID | Fold Change | Entrez Gene  ID |
| --- | --- | --- | --- |
| 1200013B08Rik | 4260400 | 2.2379625 | 74131 |
| 1810041L15Rik | 4880491 | -2.081358 | 72301 |
| 2010001M09Rik | 7210672 | 2.0513952 | 69816 |
| 2310002D06Rik | 110300 | -2.0755405 | 69522 |
| 2310058A03Rik | 5340091 | -2.214367 |  |
| 3110018K01Rik | 160136 | 2.3486698 |  |
| 381484 | 3130400 | 2.0640867 | 381484 |
| 8430408G22Rik | 5550161 | -2.7890863 | 213393 |
| 9030625A11Rik | 4200678 | -2.137883 |  |
| 9930032O22Rik | 730020 | 2.5680993 | 320454 |
| A130082M07Rik | 4260463 | 2.2077987 | 279882 |
| A130092J06Rik | 770669 | 2.350023 | 241303 |
| A430084P05Rik | 5390088 | 2.326157 | 327957 |
| AI987692 | 1660414 | 2.0118115 | 331063 |
| Actc1 | 1260669 | -2.1961923 | 11464 |
| Actc1 | 6180554 | -2.4194965 | 11464 |
| Adamts4 | 5720064 | 2.461994 |  |
| Aif1 | 1400672 | 2.1084933 | 11629 |
| Aif1 | 3940561 | 2.1360927 | 11629 |
| Aldh1a3 | 6420681 | 2.4969819 | 56847 |
| Alox12 | 4180717 | -2.0530155 | 11684 |
| Alox5 | 5820286 | 2.199046 | 11689 |
| Alox5ap | 3800372 | 2.2048147 |  |
| Alox5ap | 5270279 | 2.6524434 | 11690 |
| Apob48r | 4220189 | 2.1085887 | 171504 |
| Aqp9 | 5810452 | 2.479982 | 64008 |
| Areg | 1990373 | 4.8890724 | 11839 |
| Arhgap30 | 4250544 | 2.1972861 | 226652 |
| Arhgap4 | 7160474 | 2.4725256 | 171207 |
| B4galnt1 | 270711 | 2.1584234 | 14421 |
| BC100530 | 7150100 | 2.343929 | 100034684 |
| Bmper | 160097 | 2.268488 | 73230 |
| C030033M19Rik | 3310403 | -2.368319 |  |
| C130090K23Rik | 1710204 | 3.219592 | 231293 |
| C1qb | 580332 | 2.6119034 | 12260 |
| C1qc | 3710170 | 2.369028 | 12262 |
| C3 | 5860347 | 2.4263468 |  |
| C6 | 6220553 | 2.3990197 | 12274 |
| Car4 | 1500286 | 5.1924253 | 12351 |
| Ccdc88b | 1470079 | 2.7682834 | 78317 |
| Ccl24 | 7330681 | 4.495314 | 56221 |
| Ccl6 | 7040717 | 3.3496928 |  |
| Ccl7 | 430131 | 4.397011 |  |
| Ccl7 | 4670674 | 5.1939445 | 20306 |
| Ccl9 | 7050538 | 3.6747308 | 20308 |
| Cd163 | 1230161 | 3.7966492 | 93671 |
| Cd209d | 6250497 | 3.0200796 | 170779 |
| Cd209e | 6020630 | 5.444318 | 170780 |
| Cd3d | 610324 | 2.4558425 |  |
| Cd3g | 940747 | 2.3772912 | 12502 |
| Cd52 | 1170181 | 2.169077 |  |
| Cd52 | 6290768 | 2.674683 | 23833 |
| Cd53 | 6560301 | 3.095666 | 12508 |
| Cd74 | 6770195 | 2.282648 | 16149 |
| Cd74 | 6130072 | 2.0850742 | 16149 |
| Cd74 | 3360338 | 2.2027678 | 16149 |
| Cd83 | 7150377 | 2.300341 | 12522 |
| Cd8b1 | 1690079 | 2.040825 | 12526 |
| Cebpe | 1340634 | 2.8881445 | 110794 |
| Cfp | 1770717 | 2.069012 | 18636 |
| Ciita | 2630463 | 2.195019 | 12265 |
| Cma1 | 2650056 | 2.5993893 | 17228 |
| Cma1 | 4210039 | 3.408914 | 17228 |
| Cma2 | 2940102 | 5.4438534 | 545055 |
| Coro1a | 4290661 | 3.125882 | 12721 |
| Coro1a | 2480725 | 2.5948555 | 12721 |
| Coro1a | 1500463 | 3.1112535 | 12721 |
| Coro1a | 270661 | 2.5884335 | 12721 |
| Cpa3 | 3420672 | 2.8310366 | 12873 |
| Cpa3 | 1410739 | 4.530721 | 12873 |
| Cpa3 | 7650487 | 4.1611795 | 12873 |
| Csf1r | 3400369 | 2.2467856 | 12978 |
| Csf2rb2 | 5820528 | 3.5380883 | 12984 |
| Csf3r | 5900259 | 2.8690977 | 12986 |
| Csf3r | 6840040 | 2.4149842 | 12986 |
| Ctsg | 7650682 | 2.9248192 | 13035 |
| Ctss | 4200646 | 2.533566 | 13040 |
| Ctsz | 4150725 | 2.143727 | 64138 |
| Cxcl1 | 3610082 | 2.2743802 | 14825 |
| Cxcl13 | 3060040 | 2.4144402 |  |
| Cxcl2 | 4150750 | 2.7626843 |  |
| Cyp4f18 | 2120392 | 2.9996254 | 72054 |
| Cyth4 | 7160044 | 2.174673 | 72318 |
| Cytip | 6650070 | 4.098953 | 227929 |
| Dbp | 3180750 | -4.061441 | 13170 |
| Dennd1c | 6220044 | 2.0024521 | 70785 |
| Dok2 | 4540564 | 2.9543488 | 13449 |
| Dok3 | 610291 | 2.6189444 | 27261 |
| Dpp4 | 3060546 | 2.014763 | 13482 |
| E430021P16Rik | 6040072 | 2.4081929 |  |
| EG433016 | 4260025 | 2.9825363 | 433016 |
| EG665033 | 6560711 | 8.384138 | 665033 |
| Ear10 | 7560228 | 3.5406747 | 93725 |
| Ear11 | 1980095 | 27.139128 | 93726 |
| Ear12 | 4540543 | 3.5471437 | 503845 |
| Ear2 | 3130681 | 7.861621 | 13587 |
| Ear2 | 4070148 | 4.697641 | 13587 |
| Ear2 | 5870605 | 4.9921155 | 13587 |
| Ear2 | 2810379 | 2.6373472 | 13587 |
| Ear3 | 6860674 | 4.192631 | 53876 |
| Ear3 | 2140035 | 2.6971872 | 53876 |
| Ear3 | 4180435 | 2.9266183 | 53876 |
| Ear4 | 6250546 | 8.229246 | 53877 |
| Emb | 270341 | 2.9087722 | 13723 |
| Emilin2 | 5910164 | 2.2815664 | 246707 |
| Emilin2 | 5490026 | 2.1660318 |  |
| Emr1 | 1030411 | 2.1216671 | 13733 |
| Fbln2 | 4230228 | 2.18748 | 14115 |
| Fbxl22 | 6860475 | -2.105614 | 74165 |
| Fcer1a | 5290026 | 3.7251377 | 14125 |
| Fcer1a | 3420082 | 2.8229454 |  |
| Fcgr2b | 5700220 | 3.7088823 | 14130 |
| Fcgr2b | 1110326 | 4.0339913 | 14130 |
| Fcgr4 | 830632 | 2.9859064 | 246256 |
| Fcgrt | 7050047 | 2.0079267 | 14132 |
| Fchsd2 | 5260079 | 2.0496736 | 207278 |
| Fcna | 7200341 | 2.1645346 | 14133 |
| Fcrla | 4050093 | 2.0532293 | 98752 |
| Fgd2 | 5900440 | 2.004534 | 26382 |
| Gja1 | 1710193 | 2.2300906 | 14609 |
| Gjb2 | 6280382 | 2.2721503 | 14619 |
| Gjb2 | 2230730 | 2.3697624 | 14619 |
| Glipr2 | 3940639 | 2.0107589 | 384009 |
| Gmfg | 7050450 | 2.1271496 | 63986 |
| Gp49a | 5820739 | 3.289341 |  |
| Gpr114 | 4890092 | 2.1810627 | 382045 |
| Gpr171 | 2370039 | 4.041138 | 229323 |
| Gpr176 | 1770634 | 2.3249025 | 381413 |
| Gpx2 | 2680291 | 2.036319 | 14776 |
| Gsdmc3 | 5870520 | 2.9223223 | 270328 |
| Gsdmc3 | 4210475 | 3.4559216 | 270328 |
| Gvin1 | 450735 | 2.16242 | 74558 |
| H19 | 3290056 | 2.0765045 | 14955 |
| H2-Ab1 | 2570487 | 2.0771232 | 14961 |
| H2-Ab1 | 1440307 | 2.2984176 | 14961 |
| H2-Ab1 | 5860435 | 2.4922233 | 14961 |
| H2-DMb1 | 4900754 | 2.5251024 | 14999 |
| H2-DMb2 | 7040731 | 2.3854792 | 15000 |
| H2-Ea | 3450528 | 2.024553 | 14968 |
| H2-Eb1 | 520072 | 2.2445824 | 14969 |
| Hdc | 3890086 | 2.1757812 |  |
| Hdc | 2970324 | 5.856331 | 15186 |
| Hfe | 2360672 | 2.104139 | 15216 |
| Hist1h2ad | 7160253 | 2.1053603 | 319165 |
| Hist1h2ad | 3520717 | 2.0726242 | 319165 |
| Hist1h2af | 4250711 | 2.1639867 | 319173 |
| Hist1h2ah | 1470341 | 2.2846541 | 319168 |
| Hist1h2ah | 670739 | 2.0025668 | 319168 |
| Hist1h2ak | 3130609 | 2.2850132 | 319169 |
| Hist1h2an | 4610129 | 2.3113134 | 319170 |
| Hist1h2ao | 6510253 | 2.3018637 | 319171 |
| Hmgcs2 | 6280392 | -2.6164598 | 15360 |
| Hsd11b1 | 4050369 | 2.186432 | 15483 |
| Hsd11b1 | 4570196 | 2.118933 |  |
| Hsd11b1 | 2340301 | 2.2066052 |  |
| Ibrdc3 | 3800181 | 2.0927844 | 75234 |
| Ifi202b | 650601 | 2.0270922 | 26388 |
| Ifi47 | 1090139 | 2.3183026 | 15953 |
| Ifitm1 | 4010019 | 2.3271625 | 68713 |
| Ifitm1 | 5820068 | 2.1902342 | 68713 |
| Igf1 | 450632 | 2.6530557 | 16000 |
| Igf1 | 2000647 | 2.6973493 | 16000 |
| Igfbp4 | 5080435 | 2.4341168 |  |
| Igfbp4 | 3400747 | 2.4562917 | 16010 |
| Igh-VJ558 | 6380762 | 3.5928445 | 16061 |
| Igh-VJ558 | 240242 | 2.0975323 |  |
| Il10ra | 3990474 | 2.014572 | 16154 |
| Il11 | 7650477 | 3.3862739 | 16156 |
| Il1b | 3420754 | 6.7379546 |  |
| Il1r2 | 3420139 | 2.4356358 | 16178 |
| Il1rl1 | 3870291 | 8.9048 | 17082 |
| Il1rn | 3390131 | 2.2479107 | 16181 |
| Il33 | 3440767 | 4.6838355 | 77125 |
| Il4i1 | 6020224 | 4.624341 | 14204 |
| Il8rb | 1300095 | 2.3186572 | 12765 |
| Inmt | 2360050 | -2.4416876 | 21743 |
| Irf7 | 6590653 | 2.0414183 | 54123 |
| Itgae | 7100687 | 2.1202648 | 16407 |
| Jak3 | 4850086 | 2.067436 | 16453 |
| Kcnc4 | 4010465 | -2.1361835 | 99738 |
| Klk1 | 2760181 | 3.8006148 | 16612 |
| Klk1b26 | 6100324 | 3.059003 | 16618 |
| Klk1b27 | 1820731 | 2.3672965 | 16619 |
| Klk1b27 | 4670021 | 2.2201803 | 16619 |
| Klk1b4 | 6040626 | 2.2897754 | 18048 |
| Klk1b5 | 2060136 | 2.7531917 | 16622 |
| Kng1 | 4290521 | 3.138688 | 16644 |
| Krt14 | 1470619 | 11.348722 | 16664 |
| Krt6b | 1090730 | 3.1219485 | 16688 |
| LOC100039742 | 6220274 | 2.0286107 | 100039742 |
| LOC100044439 | 6020487 | 5.4340034 | 100044439 |
| LOC100044702 | 6180202 | 3.911222 | 100044702 |
| LOC100045877 | 3120037 | 2.4241936 | 100045877 |
| LOC100046232 | 3840521 | 2.598625 | 100046232 |
| LOC100046793 | 6330239 | 2.216379 | 100046793 |
| LOC100047162 | 6840279 | 2.6464753 | 100047162 |
| LOC100047427 | 3310672 | -3.210718 | 100047427 |
| LOC100048295 | 1660703 | 2.9611392 | 100048295 |
| LOC100048554 | 2480296 | 7.3942266 | 100048554 |
| LOC100048556 | 1990221 | 2.688732 | 100048556 |
| LOC218617 | 6580047 | 2.1953702 |  |
| LOC547343 | 1400132 | 2.0359309 | 547343 |
| LOC636944 | 7400497 | 2.0488188 | 636944 |
| LOC641240 | 780315 | 2.0850818 | 641240 |
| LOC665506 | 6760735 | 3.0711188 | 665506 |
| Laptm5 | 940438 | 2.2896698 | 16792 |
| Lcp2 | 5290647 | 2.4160228 | 16822 |
| Lgi2 | 5270139 | 2.0027027 | 246316 |
| Lrg1 | 290097 | 3.1638386 | 76905 |
| Lst1 | 5860154 | 2.0904448 |  |
| Ly86 | 430167 | 2.440537 | 17084 |
| Mcpt1 | 2680215 | 21.050032 | 17224 |
| Mcpt4 | 1820367 | 5.003541 | 17227 |
| Mcpt6 | 5670441 | 2.4493241 | 17229 |
| Mefv | 4220437 | 2.2436674 | 54483 |
| Mest | 1690019 | 2.655396 | 17294 |
| Mest | 6770356 | 3.2732356 | 17294 |
| Mgl1 | 1740546 | 2.0175028 | 17312 |
| Mgl2 | 4150497 | 2.422062 | 216864 |
| Mmp10 | 3890600 | 8.791817 | 17384 |
| Mmp13 | 5690131 | 4.9876914 | 17386 |
| Mmp3 | 1510750 | 2.2105627 | 17392 |
| Mrc1 | 4730220 | 2.973735 | 17533 |
| Ms4a6d | 3180025 | 3.5493996 | 68774 |
| Ms4a7 | 1660528 | 2.4722412 | 109225 |
| Muc16 | 7210079 | 2.2685175 | 73732 |
| Myo1g | 7200356 | 2.0528636 | 246177 |
| Napsa | 5690500 | 2.0489926 | 16541 |
| Ncf4 | 2940504 | 2.7101946 | 17972 |
| Nckap1l | 940202 | 2.127147 | 105855 |
| Nfe2 | 1170170 | 2.6536455 | 18022 |
| Nr1d1 | 6580753 | -2.5121846 | 217166 |
| Nr1d2 | 1260039 | -2.1003466 | 353187 |
| Nt5dc2 | 3460446 | 2.9828193 | 70021 |
| Nuak2 | 1770592 | 2.0057588 | 74137 |
| OTTMUSG00000000971 | 5360370 | 2.8362603 | 100034251 |
| Oas1g | 130598 | 2.2142947 | 23960 |
| Osm | 6270600 | 4.369907 | 18413 |
| P2rx1 | 110274 | -2.175576 | 18436 |
| P2ry6 | 3710403 | 2.8696015 | 233571 |
| Padi1 | 1690201 | 2.2790844 | 18599 |
| Pcp4l1 | 5080392 | -2.0199726 | 66425 |
| Pglyrp1 | 3140246 | 2.6948943 |  |
| Pira3 | 5870113 | 3.1040688 | 18726 |
| Plek | 1820075 | 2.4307272 | 56193 |
| Plvap | 3290239 | 2.2965145 | 84094 |
| Prg2 | 4280180 | 3.037782 | 19074 |
| Ptpn18 | 4050689 | 2.0356889 | 19253 |
| Ptpn6 | 4060240 | 2.0076103 | 15170 |
| Ptprc | 1230541 | 2.263889 | 19264 |
| Ptx3 | 1740008 | 2.433146 |  |
| Ptx3 | 5390136 | 2.3674352 | 19288 |
| Reg3g | 830619 | 3.4605627 | 19695 |
| Retnla | 1110709 | 7.6649776 | 57262 |
| Retnlg | 7160711 | 7.0387454 | 245195 |
| Rnase6 | 430703 | 2.4120145 | 78416 |
| S100a8 | 1190546 | 8.2229 | 20201 |
| S100a9 | 1980603 | 8.763353 | 20202 |
| Saa3 | 6400719 | 3.6947312 | 20210 |
| Samsn1 | 6020400 | 3.5194802 | 67742 |
| Scin | 3800671 | 3.0934954 | 20259 |
| Scube2 | 6590041 | -2.0124803 | 56788 |
| Sell | 5360707 | 2.7868109 | 20343 |
| Selplg | 1070307 | 2.525837 | 20345 |
| Serpina3g | 1050092 | 6.7674103 | 20715 |
| Serpina3h | 1450491 | 5.1730514 | 546546 |
| Serpina3n | 2450347 | 2.6328225 | 20716 |
| Serpinb6b | 5820056 | 3.0954428 | 20708 |
| Sla | 110064 | 2.2938972 | 20491 |
| Slc16a6 | 3120427 | 2.045833 | 104681 |
| Slco4a1 | 5820270 | 2.6664598 | 108115 |
| Slpi | 2810487 | 4.278454 | 20568 |
| Socs3 | 1570594 | 5.063469 | 12702 |
| Sprr2f | 2760669 | 9.526677 | 20760 |
| Sprr2g | 4220037 | 6.8276587 | 20761 |
| Srgn | 4610433 | 3.712274 | 19073 |
| Srgn | 5490187 | 3.6662126 | 19073 |
| Ssbp4 | 1030475 | 2.0222528 |  |
| Stab1 | 2320414 | 2.9578512 | 192187 |
| Stat4 | 5560066 | 2.3415818 | 20849 |
| Stfa1 | 940717 | 3.4037018 | 20861 |
| Stfa1 | 3360056 | 3.013967 | 20861 |
| Tbc1d10c | 380672 | 2.4563258 | 108995 |
| Tbxas1 | 5690164 | 2.6985188 | 21391 |
| Tcap | 990253 | -2.4063523 |  |
| Tcrb-V8.2 | 2510138 | 2.4498978 |  |
| Timp1 | 4640215 | 5.469879 |  |
| Timp1 | 160332 | 6.561098 | 21857 |
| Tnfrsf11b | 5220324 | 2.2393603 | 18383 |
| Tnfrsf13b | 4280296 | 2.1718771 |  |
| Tpcn2 | 2190364 | 2.0175157 | 233979 |
| Tph1 | 130066 | 2.4065766 | 21990 |
| Tpsab1 | 5130553 | 2.541569 | 17230 |
| Ttr | 2680113 | -2.5934768 | 22139 |
| Tyrobp | 450739 | 2.299063 | 22177 |
| Upp1 | 1050095 | 2.1123884 | 22271 |
| Vav1 | 5270608 | 2.2434132 | 22324 |
| Vrk1 | 3610731 | 2.5235078 |  |
| Was | 5340180 | 2.1775062 | 22376 |
| scl0001849.1_2273 | 6370020 | 3.3011901 |  |
| scl0002540.1_6 | 6280403 | 2.3676176 |  |

**WEEK 3**

| Gene Symbol | Illumina Probe ID | Fold Change | Entrez Gene  ID |
| --- | --- | --- | --- |
| 0610007P22Rik | 6590561 | -2.0043018 | 68327 |
| 0610007P22Rik | 3870091 | -2.0820475 |  |
| 0610010I05Rik | 3990427 | -2.7339053 |  |
| 1110003E01Rik | 6110577 | -2.0150824 | 68552 |
| 1110012J17Rik | 2000739 | -2.5952215 | 68617 |
| 1110018J23Rik | 6940296 | -2.0518563 |  |
| 1110059G02Rik | 6520240 | -2.0481458 |  |
| 1190020J12Rik | 7000114 | 3.3603866 | 233038 |
| 1200002N14Rik | 5270100 | 2.359826 | 71712 |
| 1200009O22Rik | 5420309 | -2.1782756 | 66873 |
| 1200013B08Rik | 4260400 | 4.7130046 | 74131 |
| 1300007L22Rik | 7610161 | -2.4820912 |  |
| 1300013J15Rik | 6520022 | -5.293731 | 67473 |
| 1700019D03Rik | 1300768 | -2.018983 | 67080 |
| 1700029G01Rik | 1110021 | -2.0533683 | 66938 |
| 1700061G19Rik | 6100546 | -2.208619 |  |
| 1700124P09Rik | 5690129 | -2.1939611 | 76998 |
| 1700124P09Rik | 7100367 | -3.3057907 | 76998 |
| 1810011H11Rik | 7550458 | 2.35982 | 69069 |
| 1810013B01Rik | 6370364 | -2.011482 |  |
| 1810019D21Rik | 7560400 | -2.5824192 |  |
| 1810041L15Rik | 4880491 | -2.7401202 | 72301 |
| 2010001M09Rik | 7210672 | 46.418354 | 69816 |
| 2010001P08Rik | 3840672 | 2.2940922 |  |
| 2010011I20Rik | 1510152 | -2.1498792 | 67017 |
| 2200001I15Rik | 460682 | -5.544731 | 69134 |
| 2300002D11Rik | 990221 | -2.5865417 | 69539 |
| 2310002D06Rik | 110300 | -4.2439685 | 69522 |
| 2310005E10Rik | 6400377 | -2.0191088 | 67861 |
| 2310014F06Rik | 6840162 | -2.213751 |  |
| 2310040A07Rik | 1940762 | -2.4852836 | 69638 |
| 2310043J07Rik | 2760333 | 2.7779996 | 69665 |
| 2310047D13Rik | 130768 | 2.2575977 |  |
| 2310047D13Rik | 7320162 | 2.3700256 | 116972 |
| 2310051E17Rik | 4050037 | -2.5706375 | 70273 |
| 2310058A03Rik | 5340091 | -3.3487873 |  |
| 2610307O08Rik | 4640328 | 2.3493054 | 72512 |
| 2810046M22Rik | 7200719 | 2.5128953 |  |
| 2900060P06Rik | 2190224 | -2.4489925 |  |
| 3100002J23Rik | 4810465 | -2.083828 | 75429 |
| 3110004L20Rik | 3990484 | -2.1667788 | 73102 |
| 3110018K01Rik | 160136 | 2.4788332 |  |
| 3632451O06Rik | 10592 | -2.3515422 | 67419 |
| 381484 | 3130400 | 2.1937983 | 381484 |
| 3830431G21Rik | 3370603 | -2.9607522 | 217682 |
| 4631405K08Rik | 1230022 | -2.2717474 |  |
| 4632417K18Rik | 1780279 | 2.460796 | 107373 |
| 4632417N05Rik | 5420332 | -3.1267457 | 74032 |
| 4733401O04Rik | 3130091 | 2.8386335 |  |
| 4833412C05Rik | 2000768 | -2.0713227 |  |
| 4921507P07Rik | 1940653 | -2.005331 | 70821 |
| 4921517B04Rik | 1570592 | -2.1933582 |  |
| 4930412O13Rik | 730484 | -2.756103 | 381347 |
| 4930426D05Rik | 3360674 | -2.588648 |  |
| 4930431B09Rik | 6840270 | 6.3512006 | 74645 |
| 4930452B06Rik | 290379 | -3.1909425 | 74430 |
| 4930572D21Rik | 5360669 | 2.800587 | 78052 |
| 4931406C07Rik | 630576 | -2.1390991 | 70984 |
| 4933428A15Rik | 5670176 | -2.5889633 |  |
| 5031414D18Rik | 1110575 | 2.0635045 | 271221 |
| 5033414K04Rik | 360053 | -2.0810695 | 98496 |
| 5430417J04Rik | 1450180 | -2.126879 |  |
| 5830411E10Rik | 2140441 | -2.4293764 |  |
| 5830496L11Rik | 3190722 | 3.1510143 |  |
| 6030408B16Rik | 3520390 | 2.025837 | 77717 |
| 6030408B16Rik | 4590451 | 2.143506 | 77717 |
| 6030495B01Rik | 2600438 | -2.0553527 |  |
| 6330404C01Rik | 2470228 | 5.931067 |  |
| 6330407A03Rik | 110670 | 2.9112785 |  |
| 6330414G02Rik | 150184 | -2.2689657 |  |
| 6330442E10Rik | 5270167 | 2.1256418 | 268567 |
| 6330500D04Rik | 4200202 | 2.9374666 | 193385 |
| 6330500D04Rik | 1450097 | 2.9027061 |  |
| 6330505N24Rik | 4390553 | -2.0194335 | 229474 |
| 6720458D17Rik | 4920491 | -2.0208652 |  |
| 8430403J19Rik | 4560747 | 2.5690327 |  |
| 8430408G22Rik | 5550161 | -5.6869802 | 213393 |
| 9030605I04Rik | 6580358 | 2.7939935 |  |
| 9030625A11Rik | 4200678 | -3.2397292 |  |
| 9130211I03Rik | 7100753 | 2.168195 | 381319 |
| 9330162L04Rik | 5090682 | -2.3150928 |  |
| 9530027K23Rik | 580255 | -2.1451545 |  |
| 9530051K01Rik | 3840187 | -3.7736647 |  |
| 9530081N05Rik | 6280689 | -2.9347153 |  |
| 9626100_224 | 1710136 | 2.1895418 |  |
| 9630015D15Rik | 2630086 | -4.3887796 |  |
| 9830134C10Rik | 450088 | 2.5240104 | 442827 |
| 9930014A18Rik | 7550196 | -2.0624766 |  |
| 9930023K05Rik | 1030438 | 2.0697074 | 226245 |
| 9930031P18Rik | 5560204 | 2.1322007 |  |
| 9930032O22Rik | 730020 | 6.8090415 | 320454 |
| 9930115F03Rik | 2630451 | -2.386491 |  |
| A130010J15Rik | 3390672 | 2.5741017 | 319266 |
| A130052C08Rik | 4180095 | 3.0999432 |  |
| A130082M07Rik | 4260463 | 3.030297 | 279882 |
| A130090K04Rik | 6650626 | 2.0060387 |  |
| A130092J06Rik | 770669 | 2.5845284 | 241303 |
| A430084P05Rik | 5390088 | 2.439458 | 327957 |
| A430107D22Rik | 670050 | 3.2835863 | 320484 |
| A630006E02Rik | 7330131 | 3.8005614 |  |
| A830073O21Rik | 2070465 | -2.6546998 |  |
| A830080H07Rik | 2470746 | 2.0717292 |  |
| A930004K21Rik | 110026 | 2.0839128 |  |
| AA407270 | 4570201 | -2.8998678 | 102371 |
| AA467197 | 7160047 | 4.698165 | 433470 |
| AI324046 | 3120113 | 34.823696 |  |
| AI324046 | 4120458 | 14.526971 |  |
| AI324046 | 1770594 | 32.31577 |  |
| AI324046 | 3130280 | 18.948978 |  |
| AI467606 | 380670 | 2.23967 | 101602 |
| AU019823 | 4890328 | -2.258632 | 270156 |
| AU020206 | 1740609 | 2.1760793 |  |
| Abca8a | 6560594 | -2.0272927 | 217258 |
| Abcb1b | 4920129 | 2.1044886 | 18669 |
| Abcc3 | 4780040 | -3.0184314 | 76408 |
| Abhd14b | 3180730 | -2.42114 | 76491 |
| Abi3 | 6450687 | 2.811723 |  |
| Acaa1b | 4780528 | -5.846115 | 235674 |
| Acaa2 | 3310424 | -2.0745087 | 52538 |
| Acaa2 | 4010398 | -2.501294 |  |
| Acaa2 | 5090414 | -2.0372002 |  |
| Ace2 | 5340450 | -2.317709 | 70008 |
| Acot1 | 520630 | -3.8787472 | 26897 |
| Acot1 | 450356 | -7.335157 | 26897 |
| Acpp | 2570280 | -2.2008893 | 56318 |
| Acsbg1 | 2900603 | 2.5481436 | 94180 |
| Acsbg1 | 3780390 | 3.0753534 | 94180 |
| Acss1 | 6200719 | 2.0176919 | 68738 |
| Acta1 | 2030450 | -3.242791 | 11459 |
| Acta2 | 3130136 | -2.0072896 |  |
| Acta2 | 4540376 | -2.0812104 |  |
| Acta2 | 1850022 | -2.1149864 | 11475 |
| Acta2 | 2140255 | -2.2408953 | 11475 |
| Actc1 | 1260669 | -5.6312947 | 11464 |
| Actc1 | 6180554 | -5.0221133 | 11464 |
| Adam28 | 7380653 | -2.4290621 | 13522 |
| Adam8 | 7200037 | 2.532111 | 11501 |
| Adam8 | 2680131 | 2.2663975 | 11501 |
| Adamts4 | 5720064 | 4.457516 |  |
| Adcy2 | 1740541 | 2.4486809 | 210044 |
| Adh1 | 110458 | -4.230651 | 11522 |
| Adh1 | 4850521 | -4.903793 | 11522 |
| Adora3 | 4070014 | 2.7450037 | 11542 |
| Adra2a | 830736 | 2.295082 | 11551 |
| Ahrr | 4070681 | -2.6151931 | 11624 |
| Aif1 | 1400672 | 2.378713 | 11629 |
| Aif1 | 290068 | 2.0102613 | 11629 |
| Aif1 | 3940561 | 2.542093 | 11629 |
| Aim1l | 5080600 | -2.0598204 | 230806 |
| Akap12 | 2320341 | -2.0048807 | 83397 |
| Akna | 1070703 | 2.1489496 | 100182 |
| Aldh1a3 | 6420681 | 9.3961735 | 56847 |
| Aldh3a1 | 5050382 | -3.7022703 | 11670 |
| Aldh5a1 | 2490717 | -2.0776825 |  |
| Alox12 | 4180717 | -2.8787682 | 11684 |
| Alox15 | 4560592 | 2.0771081 | 11687 |
| Alox15 | 6580601 | 4.7187905 | 11687 |
| Alox5 | 6960259 | 2.1903417 | 11689 |
| Alox5 | 5820286 | 3.7971046 | 11689 |
| Alox5ap | 3800372 | 3.0617552 |  |
| Alox5ap | 5270279 | 3.0375926 | 11690 |
| Amica1 | 3850692 | 2.2399306 | 270152 |
| Amn | 2510743 | -2.0263214 | 93835 |
| Angptl4 | 1710215 | 2.7392786 | 57875 |
| Angptl7 | 6520451 | -2.781277 | 654812 |
| Ank1 | 1260255 | -3.67793 | 11733 |
| Ank3 | 6020176 | -2.185271 | 11735 |
| Anxa8 | 2690215 | 3.5376105 | 11752 |
| Anxa8 | 4180239 | 3.8092756 | 11752 |
| Aof1 | 6480377 | -3.2358425 | 218214 |
| Aof1 | 6480376 | -2.499068 | 218214 |
| Aph1b | 6220612 | -2.0137486 | 208117 |
| Aplnr | 7510653 | 2.0531483 | 23796 |
| Aplp2 | 270593 | -2.0495186 | 11804 |
| Apob48r | 4220189 | 2.494521 | 171504 |
| Apoc1 | 5820600 | -2.0749736 | 11812 |
| Aqp9 | 5810452 | 3.1677768 | 64008 |
| Areg | 1990373 | 19.669369 | 11839 |
| Arg1 | 1190445 | 31.563108 | 11846 |
| Arg1 | 5360035 | 12.344723 |  |
| Arhgap12 | 5260114 | -2.1996062 | 75415 |
| Arhgap30 | 5560333 | 2.2568758 | 226652 |
| Arhgap30 | 4250544 | 4.11767 | 226652 |
| Arhgap4 | 7160474 | 4.877763 | 171207 |
| Arhgap9 | 5870470 | 2.5369983 | 216445 |
| Arhgdib | 1090180 | 3.1746278 | 11857 |
| Arl4c | 6560743 | 2.273823 | 320982 |
| Arpc1a | 2120053 | -2.2356482 | 56443 |
| Arpc1a | 6220202 | -2.172853 | 56443 |
| Arrb2 | 1110451 | 2.7151868 | 216869 |
| Art3 | 5890338 | -2.7148893 | 109979 |
| Art3 | 4260594 | -2.4980412 | 109979 |
| Asah3l | 1470386 | -6.022733 | 230379 |
| Atp2a2 | 770349 | -2.5547764 | 11938 |
| Atp2a2 | 940730 | -2.2287474 | 11938 |
| Atp2a3 | 2570672 | 2.9093838 | 53313 |
| Atp2a3 | 60288 | 3.0522418 | 53313 |
| Atrn | 3890184 | -3.803079 | 11990 |
| Aurkb | 6290390 | 2.365701 | 20877 |
| Avil | 1190427 | 2.4427452 | 11567 |
| Avpr1a | 2630195 | -3.7060125 | 54140 |
| Avpr1a | 6330482 | -6.0064163 | 54140 |
| B230114H05Rik | 2970440 | -2.2480066 |  |
| B4galnt1 | 270711 | 4.5529494 | 14421 |
| BC006779 | 6420170 | 2.435341 |  |
| BC018462 | 3780010 | -2.1581185 |  |
| BC064033 | 6840437 | -2.3496954 |  |
| BC064033 | 7380626 | -2.6198456 | 208164 |
| BC100530 | 7150100 | 8.088809 | 100034684 |
| Bank1 | 4830082 | 3.0498517 | 242248 |
| Batf | 160035 | 2.1772609 | 53314 |
| Bcas1 | 6560347 | -3.686401 | 76960 |
| Bcl11a | 460692 | 2.6583934 | 14025 |
| Bcl11a | 1570022 | 3.2762187 |  |
| Bcl11b | 6110008 | 2.9075177 | 58208 |
| Bcl11b | 7650475 | 2.0831015 | 58208 |
| Bcl2a1b | 130097 | 3.8391604 | 12045 |
| Bcl2a1d | 7040202 | 4.158689 |  |
| Bcl2l14 | 3310471 | -3.1622267 | 66813 |
| Bcl2l14 | 60452 | -2.0617168 | 66813 |
| Bcl3 | 4070020 | 3.1723156 | 12051 |
| Bcl7a | 650110 | -2.157639 | 77045 |
| Bdh2 | 240433 | -2.0203109 | 69772 |
| Bex4 | 2480523 | -2.1985812 | 406217 |
| Bglap-rs1 | 460402 | -3.34707 | 12095 |
| Bglap-rs1 | 3450521 | -2.261495 | 12095 |
| Bglap1 | 4540537 | -2.5287046 | 12096 |
| Bglap2 | 4670497 | -4.8131394 | 12097 |
| Bhlhb2 | 1230341 | 2.554527 | 20893 |
| Bhmt2 | 610576 | -2.1809635 | 64918 |
| Birc5 | 7400215 | 3.0849345 |  |
| Birc5 | 1240446 | 3.1988852 | 11799 |
| Birc5 | 2810612 | 2.9066296 | 11799 |
| Blk | 3290279 | 5.376125 | 12143 |
| Bmp3 | 940121 | -3.032583 | 110075 |
| Btk | 5050343 | 3.1959858 | 12229 |
| Bub1b | 2350095 | 2.0364633 |  |
| Bves | 580136 | -2.615937 |  |
| C030002B11Rik | 2850670 | -2.8043342 |  |
| C030014I23Rik | 5670750 | -3.0448012 |  |
| C030025P15Rik | 6130095 | -2.1772728 |  |
| C030033M19Rik | 3310403 | -2.6980212 |  |
| C130090K23Rik | 1710204 | 8.131255 | 231293 |
| C1qa | 2350132 | 2.6642838 | 12259 |
| C1qb | 580332 | 2.9817991 | 12260 |
| C1qc | 3710170 | 2.5653138 | 12262 |
| C1qtnf3 | 270671 | -2.1201742 | 81799 |
| C1qtnf3 | 5260672 | -2.6800563 | 81799 |
| C1qtnf3 | 4780180 | -2.5406294 | 81799 |
| C1qtnf4 | 5670039 | -2.1239755 | 67445 |
| C3 | 5860347 | 3.0897076 |  |
| C330006A16Rik | 7200189 | -2.209091 |  |
| C4a | 60368 | 3.5166373 | 625018 |
| C4a | 130348 | 3.6700103 | 625018 |
| C4b | 7510243 | 3.1881201 | 12268 |
| C6 | 6220553 | 4.7865486 | 12274 |
| C6 | 5690711 | 2.324388 | 12274 |
| C730013O11Rik | 520504 | 2.34787 |  |
| C920004C08Rik | 4540477 | 2.830313 |  |
| Cadps2 | 580369 | -2.1062307 | 320405 |
| Calca | 1470465 | 3.8684974 |  |
| Calcb | 5340707 | 3.2497442 | 116903 |
| Calcrl | 650138 | -2.0940478 | 54598 |
| Cald1 | 5130494 | -2.0938628 | 109624 |
| Cald1 | 4210431 | -2.2896633 | 109624 |
| Calu | 1410091 | -2.0599918 | 12321 |
| Camkk1 | 7610424 | -2.7764704 | 55984 |
| Capn13 | 3610148 | 2.331594 | 381122 |
| Capn5 | 7210022 | -2.0394077 | 12337 |
| Car4 | 1500286 | 6.990093 | 12351 |
| Card11 | 7330731 | 2.2415593 | 108723 |
| Casp1 | 1940722 | 2.1416209 | 12362 |
| Casp4 | 5290017 | 2.0668652 | 12363 |
| Casz1 | 4730440 | -2.1474278 | 69743 |
| Cav1 | 4610072 | -2.2446284 | 12389 |
| Cav2 | 4480576 | -2.4228356 | 12390 |
| Ccbl1 | 1470170 | -2.9152873 | 70266 |
| Ccbl1 | 2850746 | -2.0493112 | 70266 |
| Ccdc80 | 5810538 | 2.0874443 | 67896 |
| Ccdc88b | 1470079 | 4.3053694 | 78317 |
| Ccl11 | 830273 | 5.149929 | 20292 |
| Ccl11 | 6060520 | 6.6107497 | 20292 |
| Ccl19 | 4220717 | 2.3783462 | 24047 |
| Ccl21a | 1710612 | 3.7061825 | 18829 |
| Ccl21c | 4230315 | 3.855018 | 65956 |
| Ccl21c | 6370475 | 3.3072798 |  |
| Ccl4 | 6940184 | 3.3741899 | 20303 |
| Ccl6 | 7040717 | 3.8751647 |  |
| Ccl7 | 430131 | 5.5450068 |  |
| Ccl7 | 4670674 | 5.3170285 | 20306 |
| Ccl9 | 7050538 | 4.4330435 | 20308 |
| Ccnb1 | 2190164 | 2.6104429 | 268697 |
| Ccnb1 | 7550156 | 2.5417993 | 268697 |
| Ccnd2 | 6770286 | 2.2755208 |  |
| Ccr5 | 1780324 | 2.1628253 | 12774 |
| Ccr5 | 2120397 | 2.4610598 | 12774 |
| Ccr6 | 1010132 | 6.6001506 | 12458 |
| Ccr7 | 2570291 | 2.018141 | 12775 |
| Cd14 | 6020674 | 2.4081035 | 12475 |
| Cd177 | 7000224 | 2.0252295 | 68891 |
| Cd2 | 60221 | 2.7677698 | 12481 |
| Cd209d | 6250497 | 4.9729247 | 170779 |
| Cd209e | 6020630 | 11.775177 | 170780 |
| Cd209f | 6290093 | 2.4841506 | 69142 |
| Cd27 | 4810204 | 2.5289164 | 21940 |
| Cd274 | 3870561 | 2.3538764 | 60533 |
| Cd37 | 5050431 | 3.5292518 | 12493 |
| Cd3d | 610324 | 3.134723 |  |
| Cd3e | 6590239 | 3.2861526 | 12501 |
| Cd3g | 940747 | 4.14309 | 12502 |
| Cd40 | 4760241 | 3.422492 | 21939 |
| Cd44 | 4880138 | 2.1504478 | 12505 |
| Cd52 | 1170181 | 5.6167307 |  |
| Cd52 | 6290768 | 9.0978 | 23833 |
| Cd53 | 6560301 | 4.411975 | 12508 |
| Cd6 | 1440681 | 2.393463 | 12511 |
| Cd6 | 1030278 | 2.1789536 | 12511 |
| Cd6 | 1170246 | 2.0522664 |  |
| Cd69 | 540647 | 3.5733588 | 12515 |
| Cd72 | 5700528 | 5.985952 | 12517 |
| Cd74 | 6770195 | 3.7444353 | 16149 |
| Cd74 | 6130072 | 3.6486762 | 16149 |
| Cd74 | 3360338 | 3.444002 | 16149 |
| Cd79b | 2060373 | 12.306439 | 15985 |
| Cd79b | 2060543 | 6.172227 | 15985 |
| Cd83 | 7150377 | 3.6055932 | 12522 |
| Cd84 | 2450753 | 2.4988933 | 12523 |
| Cd86 | 2850010 | 2.0476668 | 12524 |
| Cd8b1 | 1690079 | 2.7634754 | 12526 |
| Cdc20 | 4570088 | 2.2807217 | 107995 |
| Cdc20 | 4390228 | 3.1635978 | 107995 |
| Cdc20 | 4610722 | 3.6162355 | 107995 |
| Cdc2a | 6060379 | 2.722444 | 12534 |
| Cdc42ep3 | 4150739 | -2.3984158 | 260409 |
| Cdca3 | 1050170 | 2.1184347 | 14793 |
| Cdca8 | 6450634 | 2.7698703 | 52276 |
| Cdkn3 | 1090382 | 2.2457216 | 72391 |
| Cds1 | 6270528 | -3.5543249 | 74596 |
| Cdt1 | 1050706 | 2.2698162 | 67177 |
| Ceacam1 | 1050465 | 3.4144146 | 26365 |
| Ceacam2 | 1570367 | 6.152771 | 26367 |
| Cebpb | 4230348 | 2.4682627 | 12608 |
| Cebpe | 1340634 | 4.4215755 | 110794 |
| Cebpe | 6330634 | 2.5471423 | 110794 |
| Cebpe | 3400608 | 2.3850613 | 110794 |
| Cenpa | 7200519 | 3.2822273 | 12615 |
| Cep55 | 3710050 | 2.0251772 | 74107 |
| Cfp | 1770717 | 2.3433378 | 18636 |
| Cgn | 20092 | -2.2466426 | 70737 |
| Ch25h | 1510349 | 4.2654843 | 12642 |
| Chi3l3 | 2570240 | 36.956886 | 12655 |
| Chi3l3 | 6370010 | 12.400829 | 12655 |
| Chi3l3 | 3870242 | 26.822296 |  |
| Chi3l4 | 5570327 | 9.079697 | 104183 |
| Chi3l4 | 2600180 | 44.3547 | 104183 |
| Ciita | 2630463 | 4.2988253 | 12265 |
| Cish | 4200017 | 2.6001246 | 12700 |
| Ckm | 2510053 | -2.1955845 | 12715 |
| Clca3 | 3130196 | 21.13292 | 23844 |
| Clca3 | 2690349 | 16.931965 |  |
| Clca5 | 1740167 | 2.4677424 | 229933 |
| Cldn10 | 2340056 | -2.2305102 | 58187 |
| Cldn23 | 2350398 | -2.069618 | 71908 |
| Cldn8 | 3780575 | -2.5111206 | 54420 |
| Clec4a1 | 2760259 | 2.4693646 | 269799 |
| Clec4n | 5360056 | 2.0592809 | 56620 |
| Clec7a | 1450333 | 3.504163 | 56644 |
| Clspn | 2600019 | 2.4562554 | 269582 |
| Clspn | 7570278 | 2.4269154 | 269582 |
| Cma1 | 2650056 | 6.867124 | 17228 |
| Cma1 | 4210039 | 8.732295 | 17228 |
| Cma2 | 6550086 | 4.964048 | 545055 |
| Cma2 | 2940102 | 17.77436 | 545055 |
| Cmah | 3140441 | 2.02796 |  |
| Cmtm7 | 460347 | 2.1675892 | 102545 |
| Cmtm7 | 1980768 | 2.5824146 | 102545 |
| Cnr2 | 7610743 | 2.5849388 |  |
| Cnr2 | 6590196 | 2.7831094 | 12802 |
| Cntnap2 | 6760427 | -2.390198 | 66797 |
| Cntnap2 | 620703 | -6.421075 | 66797 |
| Col17a1 | 2510064 | 3.394601 | 12821 |
| Col17a1 | 2140148 | 3.8787751 | 12821 |
| Col17a1 | 6770369 | 3.4991145 | 12821 |
| Col3a1 | 5340692 | -3.3012135 | 12825 |
| Col4a5 | 6620563 | -2.0738113 |  |
| Col4a5 | 6250463 | -2.4968047 | 12830 |
| Col6a3 | 7210392 | -2.2311502 | 12835 |
| Col7a1 | 3290209 | 2.0084076 | 12836 |
| Col8a2 | 6290221 | -2.1866426 |  |
| Coro1a | 4290661 | 7.955007 | 12721 |
| Coro1a | 2480725 | 4.604842 | 12721 |
| Coro1a | 1500463 | 7.36813 | 12721 |
| Coro1a | 270661 | 5.0994773 | 12721 |
| Cotl1 | 990438 | 2.4725578 | 72042 |
| Cotl1 | 3120703 | 2.57491 |  |
| Cox6a2 | 2470356 | 2.7827766 | 12862 |
| Cox6b2 | 7150309 | 2.1970155 | 333182 |
| Cp | 6250494 | 2.1932344 | 12870 |
| Cpa3 | 3420672 | 4.9778547 | 12873 |
| Cpa3 | 1410739 | 9.714459 | 12873 |
| Cpa3 | 7650487 | 8.886531 | 12873 |
| Creld2 | 4860079 | 5.1214933 | 76737 |
| Creld2 | 290685 | 4.5644746 | 76737 |
| Crlf1 | 3780450 | 2.8265839 | 12931 |
| Csf1r | 3400369 | 2.4386477 | 12978 |
| Csf2rb | 6760484 | 2.7207525 | 12983 |
| Csf2rb2 | 5820528 | 6.061249 | 12984 |
| Csf3r | 5900259 | 2.4879982 | 12986 |
| Csf3r | 6840040 | 2.5674217 | 12986 |
| Csgalnact1 | 5090619 | 2.2725167 | 234356 |
| Cspg5 | 7610176 | -2.3376734 | 29873 |
| Csprs | 6520392 | 3.309057 | 114564 |
| Csrp1 | 4780066 | -2.4623678 | 13007 |
| Ctla4 | 5560288 | 2.6781151 | 12477 |
| Ctnnal1 | 2320168 | -3.205606 | 54366 |
| Ctnnd2 | 150113 | -2.272547 | 18163 |
| Ctsz | 4150725 | 2.6317923 | 64138 |
| Ctsz | 3450091 | 2.877228 | 64138 |
| Cxcl1 | 3610082 | 3.2817936 | 14825 |
| Cxcl13 | 3060040 | 10.061315 |  |
| Cxcl16 | 630274 | 2.1013749 | 66102 |
| Cxcl2 | 4150750 | 2.598956 |  |
| Cxcr4 | 160392 | 3.3007922 |  |
| Cxcr6 | 7570630 | 2.0100229 | 80901 |
| Cyb5 | 7560639 | -2.0722084 | 109672 |
| Cyba | 830369 | 2.1665387 | 13057 |
| Cyhr1 | 3390605 | -2.2196596 | 54151 |
| Cyp2f2 | 6040689 | -5.4728227 | 13107 |
| Cyp2s1 | 6250180 | -2.1199536 | 74134 |
| Cyp3a57 | 7160017 | -3.1431458 | 622127 |
| Cyp4f15 | 6040392 | -6.4658227 | 106648 |
| Cyp4f18 | 2120392 | 5.784324 | 72054 |
| Cyp7b1 | 360187 | 2.6022034 | 13123 |
| Cyth4 | 7160044 | 3.166789 | 72318 |
| Cytip | 6650070 | 9.367889 | 227929 |
| D11Bwg0517e | 5700669 | -4.499131 | 52897 |
| D11Bwg0517e | 5720601 | -4.034373 | 52897 |
| D130032J17Rik | 1850474 | 2.1179616 |  |
| D13Ertd608e | 1030431 | 2.6308067 | 52673 |
| D17H6S56E-5 | 3170494 | 7.1731014 | 110956 |
| D230007K08Rik | 2940441 | 2.4478738 | 268857 |
| D230048P18Rik | 70093 | 2.1547143 |  |
| D4Bwg0951e | 3310025 | -2.5117455 | 52829 |
| D630004K10Rik | 1230743 | -2.610134 |  |
| D630008P07Rik | 6840296 | -2.0925508 |  |
| D6Mit97 | 1850452 | 36.434322 |  |
| D9Ertd280e | 6290279 | -3.4939106 |  |
| Dbc1 | 1190364 | 2.0763447 | 56710 |
| Dbp | 3180750 | -11.384092 | 13170 |
| Ddah1 | 2060592 | 2.2653284 |  |
| Decr2 | 5910349 | -2.052016 |  |
| Decr2 | 3310161 | -2.3074868 |  |
| Dennd1c | 6220044 | 4.4462833 | 70785 |
| Derl3 | 7400719 | 2.577632 | 70377 |
| Dhx58 | 5720397 | 2.172314 | 80861 |
| Dhx58 | 6330592 | 2.0447145 | 80861 |
| Dio2 | 1660129 | 2.0005045 | 13371 |
| Dkk3 | 2680189 | -2.4651697 | 50781 |
| Dkk3 | 3180682 | -2.0511904 | 50781 |
| Dlk2 | 6330672 | -2.0942926 | 106565 |
| Dm15 | 4290066 | -2.322328 |  |
| Dnajc6 | 6940537 | -2.308972 |  |
| Dnajc6 | 2070594 | -2.1959202 | 72685 |
| Dnase1l2 | 1010168 | -2.1907127 | 66705 |
| Dnase1l3 | 6520193 | 2.4894536 |  |
| Dnase2a | 6590626 | -2.0700023 |  |
| Dner | 290142 | -2.4762888 | 227325 |
| Dnmt3l | 4280100 | -2.1844842 | 54427 |
| Dnmt3l | 460746 | -2.1179562 | 54427 |
| Dock2 | 1580551 | 2.0467165 |  |
| Dok2 | 4540564 | 3.991598 | 13449 |
| Dok3 | 610291 | 4.5425353 | 27261 |
| Dsg2 | 4220554 | -2.0259907 |  |
| Dstn | 2100170 | -2.0755527 | 56431 |
| Dtna | 1260025 | -2.5611148 | 13527 |
| Duoxa2 | 1850411 | 5.094731 | 66811 |
| Dusp16 | 5550086 | -2.4402087 | 70686 |
| Dusp16 | 7160397 | -2.319758 | 70686 |
| Dusp16 | 6220279 | -2.2311227 | 70686 |
| Dusp2 | 1170521 | 3.16643 | 13537 |
| Dusp6 | 60239 | 2.7942472 | 67603 |
| Dusp6 | 6330768 | 3.0759819 | 67603 |
| E2f1 | 60369 | 2.0331943 | 13555 |
| E2f2 | 770162 | 2.1414127 | 242705 |
| E330020G21Rik | 5270687 | 2.5977159 |  |
| E430021P16Rik | 6040072 | 4.6845055 |  |
| E430033B07Rik | 2900403 | 2.105822 |  |
| EG433016 | 4260025 | 2.0013633 | 433016 |
| EG433229 | 240259 | -4.0262256 | 433229 |
| EG665033 | 6560711 | 13.090959 | 665033 |
| ENSMUSG00000068790 | 1990021 | -2.2122078 | 545007 |
| Ear10 | 7560228 | 4.8735223 | 93725 |
| Ear11 | 1980095 | 40.589558 | 93726 |
| Ear12 | 4540543 | 4.528867 | 503845 |
| Ear2 | 3130681 | 11.580773 | 13587 |
| Ear2 | 4070148 | 8.934737 | 13587 |
| Ear2 | 5870605 | 8.091071 | 13587 |
| Ear2 | 2810379 | 3.4336565 | 13587 |
| Ear3 | 6860674 | 6.7637014 | 53876 |
| Ear3 | 2140035 | 3.6477208 | 53876 |
| Ear3 | 4180435 | 3.8913355 | 53876 |
| Ear4 | 6250546 | 15.425788 | 53877 |
| Ebi2 | 2600377 | 2.1658518 |  |
| Ebpl | 5810722 | -2.3235354 | 68177 |
| Ebpl | 110630 | -2.33673 | 68177 |
| Edem1 | 4120689 | 2.0984848 | 192193 |
| Edem2 | 5220639 | 2.2727447 | 108687 |
| Edem2 | 1070678 | 2.2891197 | 108687 |
| Efs | 4610338 | -2.138859 | 13644 |
| Egln3 | 2750326 | 2.9765222 | 112407 |
| Ehd1 | 2230653 | 2.0565405 | 13660 |
| Eif4a2 | 4850747 | -2.1208358 | 13682 |
| Elf5 | 6040196 | -2.1091917 | 13711 |
| Elf5 | 3460424 | -3.2491426 | 13711 |
| Elovl4 | 1740424 | -2.4958098 | 83603 |
| Emb | 270341 | 5.135227 | 13723 |
| Emilin2 | 5910164 | 2.766193 | 246707 |
| Emilin2 | 5490026 | 2.6997972 |  |
| Emr1 | 1030411 | 2.1117659 | 13733 |
| Emr1 | 2710110 | 2.1634395 | 13733 |
| Eno3 | 3830601 | -2.702636 | 13808 |
| Enpp5 | 4850082 | -2.4045925 | 83965 |
| Entpd5 | 1240731 | -2.6515644 | 12499 |
| Entpd5 | 7650070 | -2.902479 | 12499 |
| Entpd5 | 2320292 | -2.7177875 | 12499 |
| Epb4.1l4b | 1570026 | -2.0033689 | 54357 |
| Eps8l2 | 4180132 | -2.2857819 | 98845 |
| Epsti1 | 2760274 | 2.375696 | 108670 |
| Ermp1 | 6350458 | -2.2747526 | 226090 |
| Ermp1 | 6290356 | -3.5510802 | 226090 |
| Esm1 | 3420451 | -2.1512067 | 71690 |
| Ets1 | 6020398 | 2.0743546 | 23871 |
| Evi2a | 6760255 | 2.320754 | 14017 |
| Extl1 | 7210669 | -2.1582205 | 56219 |
| Extl1 | 1340133 | -2.6271474 |  |
| F10 | 1230639 | 2.314052 |  |
| F5 | 4610138 | 3.25499 | 14067 |
| Fa2h | 2810706 | -2.1787753 | 338521 |
| Faim3 | 1990546 | 12.017371 | 69169 |
| Fbp2 | 3610347 | -2.5977783 | 14120 |
| Fbxl22 | 6860475 | -2.268044 | 74165 |
| Fcer1a | 5290026 | 8.672921 | 14125 |
| Fcer1a | 3420082 | 6.662932 |  |
| Fcgr2b | 5700220 | 3.8884647 | 14130 |
| Fcgr2b | 1110326 | 4.4881177 | 14130 |
| Fcgr3 | 3830678 | 2.752597 | 14131 |
| Fcgr4 | 830632 | 5.5190787 | 246256 |
| Fcho1 | 4390239 | 2.7874358 | 74015 |
| Fchsd2 | 5260079 | 2.0454752 | 207278 |
| Fcrla | 5220176 | 2.235747 | 98752 |
| Fcrla | 1260477 | 6.446697 | 98752 |
| Fcrla | 4050093 | 7.4352245 | 98752 |
| Fcrla | 670703 | 7.804754 | 98752 |
| Fen1 | 3460037 | 3.032374 | 14156 |
| Fer1l4 | 5960215 | -3.1372287 | 74562 |
| Fermt2 | 1780377 | -2.2201724 | 218952 |
| Fermt3 | 160259 | 2.854946 | 108101 |
| Fes | 5420739 | 2.4755397 | 14159 |
| Fetub | 4540725 | 2.8717487 | 59083 |
| Ffar2 | 6520725 | 3.8636458 | 233079 |
| Fgd2 | 5900440 | 3.1095903 | 26382 |
| Fgf10 | 2600286 | 2.7532346 | 14165 |
| Fgf10 | 130577 | 2.1447954 | 14165 |
| Fgr | 5690041 | 3.5194173 | 14191 |
| Fhod3 | 5870273 | -3.323442 | 225288 |
| Fkbp11 | 1050671 | 2.6884637 | 66120 |
| Fkbp2 | 1050082 | 2.2919478 | 14227 |
| Fmo5 | 4560300 | -8.028149 | 14263 |
| Fmo5 | 5220477 | -5.0889826 | 14263 |
| Foxq1 | 1850487 | -2.9344375 | 15220 |
| Fxyd4 | 7040139 | 2.2936585 | 108017 |
| Fxyd5 | 4780196 | 2.563173 | 18301 |
| Fxyd5 | 2630008 | 2.8888977 | 18301 |
| Galntl1 | 2690373 | -3.8847163 | 108760 |
| Gata3 | 5900070 | -3.1682148 | 14462 |
| Gch1 | 2320403 | 2.2291462 | 14528 |
| Gdpd2 | 510327 | 2.216997 | 71584 |
| Ggt5 | 7040091 | 2.60445 | 23887 |
| Gimap1 | 150433 | 2.8262484 | 16205 |
| Gimap6 | 6650477 | 2.1051931 | 231931 |
| Gimap7 | 670204 | 2.5035443 | 231932 |
| Gja1 | 1710193 | 2.7671638 | 14609 |
| Gja5 | 360133 | 2.4413095 | 14613 |
| Gjb2 | 6280382 | 5.2972946 | 14619 |
| Gjb2 | 2230730 | 5.37606 | 14619 |
| Gjb6 | 610746 | -2.4906368 | 14623 |
| Glb1l2 | 3520328 | -2.881783 | 244757 |
| Gldc | 6590097 | -4.3121333 | 104174 |
| Gldc | 5560468 | -5.0039816 | 104174 |
| Gm459 | 5720037 | 24.807112 | 243451 |
| Gm566 | 3800746 | 13.593445 | 229672 |
| Gmfg | 7050450 | 3.0093558 | 63986 |
| Gmfg | 1070091 | 3.3266125 | 63986 |
| Gmip | 1940739 | 2.3904805 | 78816 |
| Gmip | 3400114 | 2.152382 | 78816 |
| Golim4 | 4220079 | -2.1299572 | 73124 |
| Gp49a | 5820739 | 3.5037384 |  |
| Gpnmb | 4670228 | -2.1745603 | 93695 |
| Gpr109a | 5700161 | 2.0535383 | 80885 |
| Gpr114 | 4890092 | 3.2875626 | 382045 |
| Gpr171 | 2370039 | 6.6614532 | 229323 |
| Gpr18 | 2750543 | 3.8959608 | 110168 |
| Gpr65 | 5670687 | 2.910792 | 14744 |
| Gpr88 | 1470725 | 2.9210248 | 64378 |
| Gprc5a | 6060026 | 2.8569105 | 232431 |
| Gpt1 | 520634 | -2.009445 | 76282 |
| Gpx2 | 2680291 | 4.5623465 | 14776 |
| Grb7 | 780348 | -2.3582454 | 14786 |
| Grem1 | 1450445 | 3.3637872 |  |
| Grhl1 | 6270327 | -2.2134647 | 195733 |
| Grhl3 | 2340400 | -2.4908519 | 230824 |
| Grhl3 | 6280020 | -2.4335277 | 230824 |
| Grm6 | 610092 | 2.2337341 | 108072 |
| Gsdmc3 | 5870520 | 5.4016542 | 270328 |
| Gsdmc3 | 4210475 | 5.3817034 | 270328 |
| Gsn | 4260709 | -2.576511 | 227753 |
| Gsta1 | 70544 | -2.5587716 |  |
| Gsta2 | 3710465 | -2.1006143 | 14858 |
| Gsta3 | 3780193 | -3.7538276 | 14859 |
| Gsta4 | 360348 | -2.082145 | 14860 |
| Gstm1 | 3850129 | -5.1244497 | 14862 |
| Gstm2 | 7510072 | -2.191467 | 14863 |
| Gstm2 | 2690025 | -2.088445 | 14863 |
| Gstm4 | 2320228 | -2.496956 | 14865 |
| Gstm6 | 2940541 | -7.0290384 |  |
| Gsto1 | 1010435 | 2.3247972 | 14873 |
| Gstt3 | 2350324 | -3.8648534 | 103140 |
| Gtf2i | 5130343 | -2.0485196 |  |
| Gtl2 | 6940750 | -2.1499448 |  |
| Gvin1 | 450735 | 3.3535156 | 74558 |
| H19 | 1440543 | 2.3196886 | 14955 |
| H19 | 3290056 | 2.8931224 | 14955 |
| H2-Aa | 650707 | 2.1689203 | 14960 |
| H2-Ab1 | 2570487 | 2.9336107 | 14961 |
| H2-Ab1 | 1440307 | 3.2698367 | 14961 |
| H2-Ab1 | 5860435 | 3.5640771 | 14961 |
| H2-DMa | 870154 | 3.553461 | 14998 |
| H2-DMa | 150138 | 3.003783 | 14998 |
| H2-DMb1 | 4900754 | 5.0536795 | 14999 |
| H2-DMb2 | 7040731 | 5.4676743 | 15000 |
| H2-Ea | 3450528 | 2.7985952 | 14968 |
| H2-Eb1 | 520072 | 3.7935765 | 14969 |
| H2-Eb2 | 5220474 | 2.0024738 | 381091 |
| H2-K1 | 6280026 | 2.1544583 | 14972 |
| H2-Oa | 5050739 | 3.5775988 |  |
| Havcr2 | 60603 | 2.0630152 | 171285 |
| Hbegf | 6040364 | 2.7139137 | 15200 |
| Hcls1 | 7570348 | 2.557767 | 15163 |
| Hcst | 1510497 | 2.419796 | 23900 |
| Hdc | 3890086 | 2.4468968 |  |
| Hdc | 2970324 | 9.8625145 | 15186 |
| Herc1 | 5420138 | -2.2457287 | 235439 |
| Hfe | 2360672 | 2.4264247 | 15216 |
| Hgf | 5570079 | 2.5218065 |  |
| Hhip | 1410397 | -3.1941922 | 15245 |
| Hip1r | 4860070 | -2.0884242 | 29816 |
| Hist1h2ad | 7160253 | 4.537146 | 319165 |
| Hist1h2ad | 3520717 | 4.6925898 | 319165 |
| Hist1h2af | 4250711 | 4.7535744 | 319173 |
| Hist1h2ag | 70546 | 2.3036342 | 319167 |
| Hist1h2ah | 1470341 | 3.7920718 | 319168 |
| Hist1h2ah | 670739 | 3.9304442 | 319168 |
| Hist1h2ai | 5490193 | 4.5840254 | 319191 |
| Hist1h2ak | 3130609 | 3.823975 | 319169 |
| Hist1h2an | 4610129 | 4.0853386 | 319170 |
| Hist1h2ao | 6510253 | 4.3324666 | 319171 |
| Hist2h2ab | 110039 | 2.1625626 | 621893 |
| Hlx | 4180181 | 2.5589428 | 15284 |
| Hmgcs2 | 6280392 | -2.7816923 | 15360 |
| Hmha1 | 4850594 | 4.4743485 | 70719 |
| Homer2 | 2650209 | -2.739117 |  |
| Hopx | 4830612 | -2.0950954 | 74318 |
| Hp | 1170564 | 2.847208 | 15439 |
| Hp | 4390113 | 3.648538 | 15439 |
| Hp | 840044 | 4.8207073 | 15439 |
| Hpgd | 2450343 | -3.362878 |  |
| Hsd11b1 | 4050369 | 2.3849049 | 15483 |
| Hsd11b1 | 4570196 | 2.4315097 |  |
| Hsd11b1 | 2340301 | 2.5449805 |  |
| Hsd17b2 | 7320753 | -3.3978455 | 15486 |
| Hspb6 | 4040386 | -2.4754333 | 243912 |
| Htra1 | 6980093 | -2.3819854 | 56213 |
| Hvcn1 | 620646 | 2.7395205 | 74096 |
| IGHA_J00475$V00785_Ig_heavy_constant_alpha_135 | 430053 | 17.899496 |  |
| IGHG1_J00453$V00793_Ig_heavy_constant_gamma_1_792 | 6420458 | 169.73865 |  |
| IGHV12S1_M22439_Ig_heavy_variable_12S1_339 | 6520711 | 10.663907 |  |
| IGHV1S113_L33954_Ig_heavy_variable_1S113_110 | 670706 | 9.23851 |  |
| IGHV1S119_L33961_Ig_heavy_variable_1S119_14 | 2000465 | 20.742487 |  |
| IGHV1S120_AF025443_Ig_heavy_variable_1S120_8 | 4150180 | 55.490208 |  |
| IGHV1S124_AF025449_Ig_heavy_variable_1S124_11 | 20397 | 23.538416 |  |
| IGHV1S133_AF304553_Ig_heavy_variable_1S133_89 | 1770241 | 4.457494 |  |
| IGHV1S135_AF304556_Ig_heavy_variable_1S135_43 | 5870296 | 4.818854 |  |
| IGHV1S14_K00707$X00161_Ig_heavy_variable_1S14_164 | 4260717 | 37.806328 |  |
| IGHV1S28_X02460_Ig_heavy_variable_1S28_13 | 1440373 | 18.23204 |  |
| IGHV1S30_X02462_Ig_heavy_variable_1S30_12 | 1240202 | 18.921297 |  |
| IGHV1S31_X02463_Ig_heavy_variable_1S31_40 | 1660239 | 13.552545 |  |
| IGHV1S34_X02467_Ig_heavy_variable_1S34_71 | 3870139 | 30.30434 |  |
| IGHV1S35_M12376_Ig_heavy_variable_1S35_13 | 1470286 | 47.839207 |  |
| IGHV1S36_M13788_Ig_heavy_variable_1S36_40 | 1510768 | 29.974247 |  |
| IGHV1S41_X06868_Ig_heavy_variable_1S41_72 | 6900348 | 2.7426584 |  |
| IGHV1S52_M34982_Ig_heavy_variable_1S52_158 | 4920377 | 4.5787187 |  |
| IGHV1S59_L17134_Ig_heavy_variable_1S59_150 | 2230327 | 6.3662004 |  |
| IGHV2S1_V00767$J00492_Ig_heavy_variable_2S1_5 | 6580504 | 7.591173 |  |
| IGHV5S18_AF290972_Ig_heavy_variable_5S18_125 | 3610576 | 8.156576 |  |
| IGHV7S2_J00500_Ig_heavy_variable_7S2_59 | 2340703 | 8.261619 |  |
| IGHV8S6_U23021_Ig_heavy_variable_8S6_61 | 4890475 | 31.61454 |  |
| IGHV8S7_U23022_Ig_heavy_variable_8S7_163 | 3390743 | 12.458451 |  |
| IGKV12-98_AJ235949_Ig_kappa_variable_12-98_12 | 6370687 | 7.3734264 |  |
| IGKV2-137_AJ231263_Ig_kappa_variable_2-137_15 | 70619 | 17.793158 |  |
| IGKV3-2_X16954_Ig_kappa_variable_3-2_18 | 4040079 | 86.55103 |  |
| IGKV4-71_AJ231218_Ig_kappa_variable_4-71_20 | 2070300 | 47.229034 |  |
| IGKV4-73_AJ231216_Ig_kappa_variable_4-73_18 | 3850528 | 64.14907 |  |
| IGKV4-91_AJ231229_Ig_kappa_variable_4-91_29 | 1170079 | 16.120708 |  |
| IGKV8-31_AJ235957_Ig_kappa_variable_8-31_3 | 610592 | 35.796604 |  |
| IGKV9-120_V00804$J00566_Ig_kappa_variable_9-120_12 | 2680367 | 67.72477 |  |
| IGKV9-128_AJ231245_Ig_kappa_variable_9-128_15 | 5570168 | 59.966465 |  |
| IGLC2_J00595_Ig_lambda_constant_2_14 | 130162 | 67.934654 |  |
| Ibrdc3 | 3800181 | 3.3305857 | 75234 |
| Icam1 | 1450095 | 2.0308547 | 15894 |
| Id4 | 5870097 | -3.1048024 | 15904 |
| Idb4 | 4290546 | -2.8038847 |  |
| Ier3 | 6520075 | 3.076321 | 15937 |
| Ifi202b | 650601 | 2.2653036 | 26388 |
| Ifi205 | 7400528 | 2.0121038 |  |
| Ifi30 | 4290709 | 2.4383101 | 65972 |
| Ifi30 | 4730367 | 2.6522558 | 65972 |
| Ifi47 | 1090139 | 2.2520611 | 15953 |
| Ifitm1 | 4010019 | 3.0141263 | 68713 |
| Ifitm1 | 5820068 | 3.116652 | 68713 |
| Ifitm3 | 3840292 | 2.0399704 | 66141 |
| Ifitm6 | 5130059 | 2.1762629 | 213002 |
| Igf1 | 450632 | 3.8116355 | 16000 |
| Igf1 | 2000647 | 4.005129 | 16000 |
| Igfbp3 | 4920288 | 3.6787028 | 16009 |
| Igfbp3 | 1820601 | 4.2637224 | 16009 |
| Igfbp4 | 5080435 | 4.4059653 |  |
| Igfbp4 | 3400747 | 2.8221042 | 16010 |
| Igfbp5 | 5080292 | 2.3857973 | 16011 |
| Igfbp5 | 160100 | 2.3782244 | 16011 |
| Igh-4 | 5960402 | 103.465775 |  |
| Igh-6 | 5340278 | 33.170227 |  |
| Igh-6 | 1010138 | 36.237247 |  |
| Igh-V11 | 3800246 | 31.688501 |  |
| Igh-VJ558 | 6380762 | 140.13147 | 16061 |
| Igh-VJ558 | 240242 | 154.08391 |  |
| Igh-VS107 | 3120301 | 15.70856 |  |
| Ighg | 1110253 | 76.99476 | 380794 |
| Ighg | 6660156 | 8.642649 | 380794 |
| Igj | 4250162 | 7.5016994 | 16069 |
| Igk-C | 630730 | 17.859613 |  |
| Igk-V38 | 830576 | 27.91223 |  |
| Igk-V38 | 5340196 | 29.864084 |  |
| Igk-V5 | 2030372 | 23.687693 | 381777 |
| Igk-V5 | 4640113 | 39.90227 | 381777 |
| Igl-V1 | 5080091 | 123.399414 |  |
| Igl-V1 | 4730180 | 190.70491 |  |
| Igtp | 6290037 | 2.5428255 | 16145 |
| Ikzf1 | 2070070 | 2.5013182 | 22778 |
| Ikzf1 | 7000446 | 2.1243806 | 22778 |
| Il10ra | 3990474 | 3.8349266 | 16154 |
| Il11 | 7650477 | 10.075335 | 16156 |
| Il13ra2 | 5820446 | 5.486401 | 16165 |
| Il16 | 2760427 | 3.2363138 | 16170 |
| Il17rd | 7400603 | -2.2422047 | 171463 |
| Il17re | 50706 | -2.2719567 | 57890 |
| Il17re | 7100020 | -2.5866477 | 57890 |
| Il17re | 6480369 | -2.9297893 | 57890 |
| Il19 | 3940168 | 2.3631437 | 329244 |
| Il1b | 3420754 | 10.053833 |  |
| Il1r2 | 3420139 | 3.345354 | 16178 |
| Il1rl1 | 3870291 | 21.953129 | 17082 |
| Il1rn | 3390131 | 3.2446058 | 16181 |
| Il24 | 6180609 | 5.799424 | 93672 |
| Il27ra | 6620446 | 2.700322 | 50931 |
| Il2rg | 270176 | 2.7591403 | 16186 |
| Il33 | 3440767 | 11.50122 | 77125 |
| Il4 | 1300445 | 3.4819696 | 16189 |
| Il4i1 | 6020224 | 8.8437195 | 14204 |
| Il4ra | 130093 | 2.7524705 | 16190 |
| Il6 | 5670731 | 3.2943664 | 16193 |
| Ildr1 | 2190382 | -3.030963 | 106347 |
| Ildr1 | 3850747 | -3.4798455 | 106347 |
| Incenp | 630634 | 2.3730416 | 16319 |
| Inhba | 3060458 | 3.4706993 | 16323 |
| Inmt | 4640221 | -3.9304206 | 21743 |
| Inmt | 2360050 | -4.2206707 | 21743 |
| Inpp5d | 4040523 | 3.0101833 | 16331 |
| Irf4 | 1010397 | 5.763458 | 16364 |
| Irf5 | 3140646 | 2.5646136 | 27056 |
| Irf7 | 6590653 | 2.276214 | 54123 |
| Irf8 | 4610373 | 2.7256672 | 15900 |
| Itga11 | 6960397 | -5.8400884 | 319480 |
| Itga11 | 4050136 | -3.9470575 | 319480 |
| Itgae | 520196 | 2.021783 | 16407 |
| Itgal | 1170091 | 2.9318242 | 16408 |
| Itgb7 | 830543 | 4.546083 | 16421 |
| Itgbl1 | 3800139 | -2.9868627 | 223272 |
| Itk | 6180154 | 2.450408 | 16428 |
| Jak1 | 6650039 | -3.2470746 | 16451 |
| Jak3 | 2510392 | 2.0602694 | 16453 |
| Jak3 | 4850086 | 2.8408015 | 16453 |
| Jam4 | 2370246 | -2.667387 | 72058 |
| Junb | 3120014 | 2.3757756 | 16477 |
| Kazald1 | 1230068 | -3.1828914 | 107250 |
| Kcnab2 | 7400575 | 3.653287 | 16498 |
| Kcnc4 | 4010465 | -2.587716 | 99738 |
| Kcne1 | 2190112 | -2.3636465 | 16509 |
| Kcnf1 | 7380228 | -4.6499863 | 382571 |
| Kcnk1 | 1450608 | -2.2586336 | 16525 |
| Kcnk1 | 5860228 | -2.0519667 |  |
| Kcnk2 | 6290452 | -2.395179 | 16526 |
| Kif15 | 6330114 | 2.2886395 | 209737 |
| Kif22 | 4210246 | 2.7201145 | 110033 |
| Kif2c | 1780543 | 2.2309813 | 73804 |
| Klf9 | 4210327 | -2.2984369 | 16601 |
| Klhl6 | 5490358 | 3.0130243 | 239743 |
| Klhl6 | 6860040 | 2.8683755 | 239743 |
| Klk1 | 2760181 | 6.047008 | 16612 |
| Klk1b26 | 6100324 | 4.2337174 | 16618 |
| Klk1b27 | 1820731 | 2.574621 | 16619 |
| Klk1b27 | 4670021 | 2.6762455 | 16619 |
| Klk1b4 | 6040626 | 2.8909976 | 18048 |
| Klk1b5 | 2060136 | 3.351952 | 16622 |
| Kndc1 | 6960376 | 4.72221 | 76484 |
| Kng1 | 4290521 | 5.429724 | 16644 |
| Krt13 | 4610414 | 16.02087 | 16663 |
| Krt14 | 1470619 | 36.979355 | 16664 |
| Krt16 | 2970392 | 3.4151468 | 16666 |
| Krt17 | 1780341 | 3.204045 | 16667 |
| Krt18 | 990424 | -2.85081 | 16668 |
| Krt20 | 4610327 | -2.2099144 | 66809 |
| Krt23 | 4780164 | 4.230172 | 94179 |
| Krt4 | 4920438 | 4.33471 | 16682 |
| Krt6a | 3990086 | 4.0289574 | 16687 |
| Krt6a | 840528 | 4.1855903 | 16687 |
| Krt6b | 1090730 | 16.89431 | 16688 |
| Krt6b | 670397 | 2.5496123 | 16688 |
| Krtdap | 830333 | -2.493254 | 64661 |
| Kynu | 520138 | 2.1568031 | 70789 |
| LOC100038908 | 2510296 | 2.163191 | 100038908 |
| LOC100039742 | 6220274 | 4.057038 | 100039742 |
| LOC100040671 | 2940673 | -2.0164604 | 100040671 |
| LOC100041004 | 460754 | 2.1471264 | 100041004 |
| LOC100041103 | 7400400 | -3.1052678 | 100041103 |
| LOC100041137 | 3140307 | 2.0233474 | 100041137 |
| LOC100041137 | 4180048 | 2.634223 | 100041137 |
| LOC100041504 | 6420376 | 4.144068 | 100041504 |
| LOC100041569 | 3800300 | -2.2768884 | 100041569 |
| LOC100042270 | 1770301 | 7.2201014 | 100042270 |
| LOC100042514 | 5260053 | 2.2129235 | 100042514 |
| LOC100043918 | 1740196 | 2.2139804 | 100043918 |
| LOC100043991 | 4810392 | 11.956407 | 100043991 |
| LOC100044430 | 2190133 | 2.3845716 | 100044430 |
| LOC100044439 | 6020487 | 11.855326 | 100044439 |
| LOC100044538 | 4180537 | 3.3182898 | 100044538 |
| LOC100044683 | 1580520 | -2.5051463 | 100044683 |
| LOC100044702 | 6180202 | 7.882365 | 100044702 |
| LOC100045250 | 1500082 | 2.283357 | 100045250 |
| LOC100045250 | 6980050 | 2.056683 | 100045250 |
| LOC100045280 | 2120377 | -2.1169848 | 100045280 |
| LOC100045280 | 6200523 | -2.2439158 | 100045280 |
| LOC100045391 | 4260086 | 5.4140086 | 100045391 |
| LOC100045567 | 4890113 | 2.0886161 | 100045567 |
| LOC100045678 | 4860092 | 2.0639398 | 100045678 |
| LOC100045680 | 1010632 | 3.4694479 | 100045680 |
| LOC100045877 | 3120037 | 5.5452094 | 100045877 |
| LOC100046087 | 4860482 | 2.0750651 | 100046087 |
| LOC100046129 | 1770441 | -2.2030044 | 100046129 |
| LOC100046232 | 3840521 | 4.975421 | 100046232 |
| LOC100046250 | 1690047 | 2.4082959 | 100046250 |
| LOC100046275 | 4260735 | 16.184336 | 100046275 |
| LOC100046496 | 3780368 | 18.752413 | 100046496 |
| LOC100046546 | 1450538 | 19.674862 | 100046546 |
| LOC100046552 | 1770767 | 13.1875105 | 100046552 |
| LOC100046690 | 6650424 | -3.5999858 | 100046690 |
| LOC100046793 | 6330239 | 61.41582 | 100046793 |
| LOC100046793 | 6960673 | 79.38628 | 100046793 |
| LOC100047053 | 5700347 | 32.89114 | 100047053 |
| LOC100047053 | 6180747 | 52.55576 | 100047053 |
| LOC100047132 | 2940167 | 6.2096877 | 100047132 |
| LOC100047132 | 520343 | 9.124109 | 100047132 |
| LOC100047162 | 6840279 | 63.289524 | 100047162 |
| LOC100047200 | 6510162 | -2.0276632 | 100047200 |
| LOC100047316 | 6270072 | 63.345673 | 100047316 |
| LOC100047316 | 2690538 | 14.744435 | 100047316 |
| LOC100047385 | 3440215 | -2.382787 | 100047385 |
| LOC100047427 | 3310672 | -2.6607022 | 100047427 |
| LOC100047579 | 4230368 | -4.686407 | 100047579 |
| LOC100047583 | 1400053 | 2.4161637 | 100047583 |
| LOC100047619 | 4180437 | 2.14268 | 100047619 |
| LOC100047628 | 3400100 | 22.26154 | 100047628 |
| LOC100047776 | 5890273 | 3.1845891 | 100047776 |
| LOC100047788 | 2760243 | 32.791992 | 100047788 |
| LOC100047788 | 4810040 | 35.473804 | 100047788 |
| LOC100047815 | 1340553 | 11.603902 | 100047815 |
| LOC100047816 | 7550110 | -2.1500943 | 100047816 |
| LOC100047934 | 2140136 | 2.3386967 | 100047934 |
| LOC100048554 | 2480296 | 8.866194 | 100048554 |
| LOC100048556 | 2510333 | 2.9492073 | 100048556 |
| LOC100048556 | 1990221 | 3.332835 | 100048556 |
| LOC100048770 | 4670091 | 21.14351 | 100048770 |
| LOC192690 | 7050259 | 3.1374476 |  |
| LOC207685 | 5860133 | 142.04541 | 207685 |
| LOC207685 | 6250753 | 8.707587 | 207685 |
| LOC207685 | 5420128 | 10.726525 | 207685 |
| LOC213684 | 3780639 | 65.54082 |  |
| LOC218617 | 6580047 | 3.4687278 |  |
| LOC226017 | 3370286 | -3.8658729 |  |
| LOC232060 | 1030373 | 36.364426 |  |
| LOC232065 | 1710446 | 33.134003 |  |
| LOC238447 | 6760307 | 83.525 |  |
| LOC240672 | 4040382 | 2.287444 |  |
| LOC243431 | 4220100 | 95.43108 | 243431 |
| LOC243439 | 6550477 | 20.913671 | 243439 |
| LOC268853 | 2650403 | -2.1728778 |  |
| LOC270152 | 6940398 | 2.1804786 |  |
| LOC272683 | 5490156 | 52.994923 |  |
| LOC380801 | 2370386 | 6.9603252 |  |
| LOC380805 | 5310040 | 14.655334 |  |
| LOC381140 | 5900370 | 3.1712942 |  |
| LOC381284 | 7100747 | 2.321883 |  |
| LOC381739 | 1410241 | -2.1245682 |  |
| LOC381774 | 1580750 | 43.319637 |  |
| LOC381782 | 4890176 | 4.2753997 |  |
| LOC382646 | 6980681 | 3.8408365 |  |
| LOC383196 | 6110220 | 17.228062 | 383196 |
| LOC383196 | 5870358 | 17.833141 | 383196 |
| LOC384273 | 1500703 | -2.5678287 |  |
| LOC384412 | 7550706 | 7.50458 |  |
| LOC384413 | 1050731 | 97.82227 |  |
| LOC384419 | 6580309 | 18.732763 |  |
| LOC384422 | 2340022 | 21.646286 |  |
| LOC385109 | 7200039 | 58.34309 |  |
| LOC385277 | 7380064 | 11.089409 |  |
| LOC385291 | 3870307 | 51.35187 |  |
| LOC385644 | 60132 | -2.8450954 |  |
| LOC386520 | 6220471 | 23.555515 |  |
| LOC432709 | 2570743 | 7.6419125 | 432709 |
| LOC432709 | 620719 | 26.511978 | 432709 |
| LOC433943 | 6860070 | -7.635959 | 433943 |
| LOC434031 | 3890685 | 41.11948 | 434031 |
| LOC544904 | 780064 | 21.134413 | 544904 |
| LOC545013 | 6180379 | -2.2167025 | 545013 |
| LOC547380 | 1690368 | -2.1580446 | 547380 |
| LOC624610 | 6400347 | 7.237142 | 624610 |
| LOC626347 | 110523 | 10.197369 | 626347 |
| LOC630242 | 7400692 | 20.235601 | 630242 |
| LOC630253 | 6330193 | 14.321122 | 630253 |
| LOC630284 | 1780370 | 30.612946 | 630284 |
| LOC630284 | 1300195 | 12.213468 | 630284 |
| LOC630302 | 460521 | 9.179679 | 630302 |
| LOC630302 | 2850228 | 18.22613 | 630302 |
| LOC630305 | 4640364 | 33.75252 | 630305 |
| LOC630337 | 3290139 | 61.10546 | 630337 |
| LOC630347 | 4890471 | 41.952324 | 630347 |
| LOC630837 | 2230129 | 12.872279 | 630837 |
| LOC633273 | 3870240 | 6.0587635 | 633273 |
| LOC635601 | 450327 | 23.284037 | 635601 |
| LOC635601 | 5810575 | 22.557903 | 635601 |
| LOC635815 | 7200672 | 77.27732 | 635815 |
| LOC636696 | 5700241 | 38.25653 | 636696 |
| LOC636752 | 20132 | 54.023907 | 636752 |
| LOC636818 | 3290543 | 2.5593996 | 636818 |
| LOC636875 | 5050703 | 17.426977 | 636875 |
| LOC636944 | 7400497 | 95.86029 | 636944 |
| LOC636944 | 1990706 | 90.26935 | 636944 |
| LOC637155 | 5900010 | 7.076396 | 637155 |
| LOC637227 | 2190021 | 57.503857 | 637227 |
| LOC637260 | 2260504 | 14.5043955 | 637260 |
| LOC637785 | 3850241 | 54.171993 | 637785 |
| LOC638301 | 6450259 | 2.9955122 | 638301 |
| LOC640696 | 5390553 | 7.354585 | 640696 |
| LOC640739 | 1990270 | 2.3377926 | 640739 |
| LOC641221 | 4060324 | 12.616822 | 641221 |
| LOC641240 | 780315 | 3.1545296 | 641240 |
| LOC665506 | 6760735 | 5.1742845 | 665506 |
| LOC666053 | 6900504 | -2.3741546 | 666053 |
| LOC669053 | 5690300 | 73.20903 | 669053 |
| LOC672329 | 6520437 | 17.032515 | 672329 |
| LOC672339 | 4570408 | 43.714523 | 672339 |
| LOC672342 | 1070356 | 56.672104 | 672342 |
| LOC674094 | 6980168 | 12.558909 | 674094 |
| LOC674107 | 10367 | 5.1059976 | 674107 |
| LOC674110 | 1240347 | 16.501934 | 674110 |
| LOC674147 | 2230112 | 14.454593 | 674147 |
| LOC674147 | 1090376 | 16.02979 | 674147 |
| LOC676124 | 540014 | 2.8604147 | 676124 |
| LOC676136 | 3190687 | 66.47566 | 676136 |
| LOC676136 | 7000202 | 99.08706 | 676136 |
| LOC676222 | 4780379 | 116.35844 | 676222 |
| LOC676389 | 5360278 | 2.2855544 | 676389 |
| LOC676420 | 1190377 | 3.0207095 | 676420 |
| LOC677369 | 5310162 | 2.0660746 | 677369 |
| LOC677643 | 50064 | 72.91404 | 677643 |
| LOC677648 | 610433 | 9.735948 | 677648 |
| Lama2 | 5910039 | -2.1274714 | 16773 |
| Laptm5 | 940438 | 4.0852346 | 16792 |
| Lass3 | 4880075 | -2.2238479 | 545975 |
| Lat | 6900044 | 2.6608038 | 16797 |
| Lat2 | 4830326 | 2.5500102 | 56743 |
| Lat2 | 6370553 | 4.68149 | 56743 |
| Lck | 1820541 | 2.9079285 | 16818 |
| Lcp1 | 1470711 | 2.2273521 | 18826 |
| Lcp2 | 2350241 | 2.2456996 |  |
| Lcp2 | 5290647 | 3.5215685 | 16822 |
| Ldhd | 1580592 | 2.587071 | 52815 |
| Ldhd | 1430689 | 2.4024625 | 52815 |
| Lgi2 | 5270139 | 2.2841597 | 246316 |
| Lgmn | 1690719 | 2.7743864 | 19141 |
| Lilrb4 | 830072 | 2.4646733 | 14728 |
| Lims2 | 4040438 | -2.127927 | 225341 |
| Lims2 | 3180681 | -2.796962 | 225341 |
| Lims2 | 2350682 | -2.659849 | 225341 |
| Liph | 380332 | -2.5091825 | 239759 |
| Lmod1 | 2470458 | -2.0654502 | 93689 |
| Lonp2 | 6180300 | -2.1996887 | 66887 |
| Lpxn | 7050433 | 3.7999544 | 107321 |
| Lrg1 | 290097 | 6.8711267 | 76905 |
| Lrmp | 5390064 | 2.9830122 | 16970 |
| Lrmp | 5340369 | 4.135036 |  |
| Lrrc33 | 7050743 | 3.1503904 | 224109 |
| Lst1 | 5860154 | 3.1052306 |  |
| Ltb4r1 | 4230402 | 2.2207227 | 16995 |
| Ltf | 6940037 | 5.384002 | 17002 |
| Ly6c1 | 5550671 | 2.9273188 | 17067 |
| Ly6e | 6550376 | 2.0490873 | 17069 |
| Ly6e | 4780184 | 2.2046642 |  |
| Ly86 | 430167 | 3.731721 | 17084 |
| Lyl1 | 360195 | 2.7219462 | 17095 |
| Lypd6b | 110326 | -2.0433142 | 71897 |
| MALT-1 | 4070768 | 2.680181 |  |
| Mad | 6660039 | 2.7579374 |  |
| Mall | 5220041 | 2.8293047 | 228576 |
| Map3k14 | 5690220 | 2.2722642 | 53859 |
| Map4k1 | 4010097 | 3.653459 | 26411 |
| Mapk11 | 5690358 | 2.1933599 | 19094 |
| 1-Mar | 2680669 | 2.282642 | 72925 |
| Marveld3 | 4590110 | -2.4270384 | 73608 |
| Matk | 1940646 | 2.118131 | 17179 |
| Mcm10 | 4220326 | 2.5757904 | 70024 |
| Mcm2 | 1770612 | 2.0209146 | 17216 |
| Mcm5 | 6220270 | 3.2441096 | 17218 |
| Mcm6 | 270379 | 2.6570003 | 17219 |
| Mcm6 | 3290437 | 2.9582648 | 17219 |
| Mcoln2 | 1050184 | 2.1156547 | 68279 |
| Mcpt1 | 2680215 | 105.4663 | 17224 |
| Mcpt4 | 1820367 | 9.43349 | 17227 |
| Mcpt6 | 5670441 | 8.0911 | 17229 |
| Mcpt8 | 2650382 | 2.0758247 | 17231 |
| Mef2c | 1230440 | 2.4406805 | 17260 |
| Mef2c | 5820369 | 2.9540095 | 17260 |
| Mef2c | 2570427 | 2.1783824 | 17260 |
| Mefv | 4220437 | 2.4409015 | 54483 |
| Mela | 3940129 | 2.452722 |  |
| Mest | 6770356 | 2.6399024 | 17294 |
| Mfap3l | 5860446 | -2.2359667 | 71306 |
| Mfsd2 | 2350368 | 4.640341 | 76574 |
| Mfsd7c | 380132 | -2.265488 | 217721 |
| Mgl1 | 1740546 | 3.0592942 | 17312 |
| Mgl2 | 1010047 | 3.2150414 |  |
| Mgl2 | 4150497 | 4.323873 | 216864 |
| Mgst1 | 7610114 | -2.0453284 | 56615 |
| Mgst2 | 7330292 | -3.137073 | 211666 |
| Mist1 | 2030669 | 2.9252057 |  |
| Mki67 | 5360129 | 4.396223 | 17345 |
| Mmp10 | 3890600 | 18.275768 | 17384 |
| Mmp13 | 5690131 | 16.287804 | 17386 |
| Mmp3 | 6180544 | 3.320551 | 17392 |
| Mmp3 | 5270670 | 2.1702433 | 17392 |
| Mmp3 | 1510750 | 4.121096 | 17392 |
| Mmp9 | 1570551 | 3.9003484 | 17395 |
| Mocs1 | 60600 | -2.8751962 | 56738 |
| Mras | 160451 | -2.1725197 | 17532 |
| Mrc1 | 4730220 | 2.644192 | 17533 |
| Ms4a6d | 2190475 | 2.8695347 | 68774 |
| Ms4a6d | 3180025 | 3.2872505 | 68774 |
| Ms4a7 | 1660528 | 2.143214 | 109225 |
| Ms4a8a | 2340463 | 2.1216526 | 64381 |
| Msi2 | 6650082 | -2.3586679 | 76626 |
| Msn | 1780474 | 2.1113958 |  |
| Msr2 | 6130746 | 2.1942043 | 80891 |
| Msx3 | 5810626 | 2.7895713 |  |
| Mthfd2 | 4210121 | 2.5727735 |  |
| Muc13 | 4880386 | 3.538955 | 17063 |
| Mx2 | 520278 | 2.0777826 |  |
| Mycl1 | 1580445 | -2.5574112 | 16918 |
| Myh10 | 7040608 | -2.7084901 | 77579 |
| Myl9 | 1170307 | -2.1657784 |  |
| Myl9 | 50148 | -2.0350013 | 98932 |
| Myo18b | 4560070 | -2.3987114 | 74376 |
| Myo1g | 7200356 | 2.2627172 | 246177 |
| Myoc | 7400463 | -3.4923346 | 17926 |
| Myocd | 5860674 | -2.5059347 | 214384 |
| Myocd | 6760673 | -2.69778 | 214384 |
| Naip5 | 2060674 | 2.0626493 | 17951 |
| Napsa | 5690500 | 5.4221487 | 16541 |
| Napsa | 380369 | 4.0929327 | 16541 |
| Napsa | 4830551 | 3.2600565 | 16541 |
| Ncapd2 | 4480070 | 2.1325228 | 68298 |
| Ncf1 | 3520300 | 2.448382 | 17969 |
| Ncf4 | 2940504 | 3.427193 | 17972 |
| Nckap1l | 940202 | 2.800393 | 105855 |
| Ndrg2 | 2630543 | -3.8170016 |  |
| Ndrg2 | 1450601 | -2.9745102 | 29811 |
| Nek3 | 4540523 | -2.2538073 | 23954 |
| Neo1 | 1090288 | -2.0918415 |  |
| Neurl | 730754 | -2.2150972 |  |
| Nfe2 | 1170170 | 3.0816891 | 18022 |
| Nfe2l2 | 1110201 | -2.3269784 | 18024 |
| Nfe2l3 | 4200671 | -2.1454206 |  |
| Nfe2l3 | 2480204 | -2.1340363 | 18025 |
| Nfe2l3 | 7650685 | -2.4807756 | 18025 |
| Nfkb1 | 5050541 | 2.036657 |  |
| Nfkb1 | 4180669 | 2.0122175 | 18033 |
| Nfkbid | 630091 | 2.6325662 | 243910 |
| Nfkbie | 6420450 | 2.4397423 | 18037 |
| Ngfr | 4050064 | 2.886265 | 18053 |
| Nhlrc1 | 1710736 | -2.037249 | 105193 |
| Nicn1 | 5490343 | -2.0057342 | 66257 |
| Nipsnap1 | 3990397 | -2.9163675 | 18082 |
| Nkd2 | 5560100 | -2.4919772 | 72293 |
| Nope | 2230687 | -2.7700613 | 56741 |
| Notum | 1820433 | -3.8980348 | 77583 |
| Nptx2 | 3120497 | -2.2377684 | 53324 |
| Npy6r | 520411 | -3.481035 | 18169 |
| Nqo1 | 4010673 | -4.745511 | 18104 |
| Nqo1 | 780301 | -2.0543551 | 18104 |
| Nr1d1 | 6580753 | -2.7206662 | 217166 |
| Nr1d2 | 1260039 | -3.1727412 | 353187 |
| Nr1d2 | 4730019 | -2.210598 | 353187 |
| Nrarp | 4810328 | -2.5212824 | 67122 |
| Nrg1 | 2490039 | 2.0410829 | 211323 |
| Nt5dc2 | 3460446 | 2.1054904 | 70021 |
| Nt5e | 3610619 | -2.2506971 | 23959 |
| Nuak2 | 1770592 | 3.9300628 | 74137 |
| Nudt11 | 1170553 | -2.0015574 | 58242 |
| Nup210 | 1230703 | 4.0653205 | 54563 |
| Nusap1 | 1500491 | 2.6009977 | 108907 |
| ORF9 | 4810259 | 2.5917017 | 52793 |
| ORF9 | 1230653 | 2.4651086 | 52793 |
| OTTMUSG00000000971 | 5360370 | 3.6125338 | 100034251 |
| OTTMUSG00000004551 | 1990259 | -2.0969274 | 100038514 |
| Oas1g | 130598 | 2.543488 | 23960 |
| Oas1g | 3890328 | 2.934862 | 23960 |
| Oas2 | 3060450 | 2.2046137 | 246728 |
| Oasl1 | 4780253 | 2.285547 | 231655 |
| Ociad2 | 6350133 | -2.3331666 | 433904 |
| Ociad2 | 4880494 | -2.4683254 | 433904 |
| Ociad2 | 3520053 | -2.1606367 | 433904 |
| Odc1 | 6840121 | 2.029653 | 18263 |
| Odz4 | 5310497 | 2.3513854 | 23966 |
| Olfml3 | 1190202 | 2.3347898 | 99543 |
| Osbpl2 | 6270022 | -2.3873906 | 228983 |
| Osbpl6 | 2940114 | -2.094105 | 99031 |
| Osgin1 | 430037 | -3.184023 | 71839 |
| Osm | 6270600 | 6.6692476 | 18413 |
| Ovol2 | 110500 | -2.3641465 | 107586 |
| P2rx1 | 110274 | -3.7450337 | 18436 |
| P2rx1 | 6220360 | -4.6940928 | 18436 |
| P2ry14 | 1240538 | 2.1972415 | 140795 |
| P2ry14 | 6580379 | 3.3214774 | 140795 |
| P2ry6 | 3710403 | 3.3505871 | 233571 |
| Padi1 | 1690201 | 5.4901724 | 18599 |
| Pafah2 | 7650148 | -2.0415025 | 100163 |
| Paqr5 | 5860288 | -2.9312594 | 74090 |
| Paqr6 | 2750632 | -2.2686014 | 68957 |
| Parvg | 3310612 | 2.4940882 | 64099 |
| Pbk | 2900300 | 2.9826148 | 52033 |
| Pcp4l1 | 2100609 | -2.5268362 | 66425 |
| Pcyox1 | 6940431 | -2.461207 |  |
| Pdcd1lg2 | 2070035 | 2.0794878 | 58205 |
| Pdcd4 | 5310600 | -2.067863 | 18569 |
| Pde4b | 870195 | 3.3903737 |  |
| Pdgfrl | 1110440 | -2.2721543 | 68797 |
| Pdia4 | 4040274 | 2.8637903 | 12304 |
| Pdlim1 | 7560673 | 2.3395722 | 54132 |
| Pdlim3 | 650452 | -2.8547218 | 53318 |
| Pdlim3 | 2000482 | -2.654881 | 53318 |
| Pdlim4 | 2940615 | -2.0096648 | 30794 |
| Pecam1 | 1030739 | 2.3016152 | 18613 |
| Pecam1 | 5670020 | 2.2474763 | 18613 |
| Peg3 | 2690435 | -2.7164595 | 18616 |
| Per2 | 5900497 | -2.5785663 | 18627 |
| Per2 | 5670424 | -3.0059574 | 18627 |
| Per3 | 6110753 | -2.423314 |  |
| Pfkm | 6250672 | -2.0932934 | 18642 |
| Pfn2 | 290672 | -2.8967 | 18645 |
| Pfn2 | 5720241 | -2.5201287 | 18645 |
| Pglyrp1 | 3140246 | 3.50242 |  |
| Pgm5 | 3400719 | -2.3977203 | 226041 |
| Pgm5 | 7100504 | -2.2230964 | 226041 |
| Pgrmc1 | 2680408 | -2.537449 | 53328 |
| Phlda1 | 4480180 | 2.3989623 | 21664 |
| Pigh | 1570458 | -2.0899742 | 110417 |
| Pigh | 2370563 | -2.1995392 | 110417 |
| Pik3ap1 | 770189 | 2.1549022 | 83490 |
| Pik3cd | 6560017 | 3.8020263 | 18707 |
| Pik3cg | 3450075 | 3.4256935 | 30955 |
| Pik3cg | 4850291 | 2.1043358 |  |
| Pim1 | 5860039 | 2.370514 | 18712 |
| Pip4k2a | 3140678 | 2.0925598 | 18718 |
| Pira3 | 5870113 | 5.8734612 | 18726 |
| Pira4 | 7330114 | 2.6808627 | 18727 |
| Pitx2 | 1820746 | -2.1269927 | 18741 |
| Pitx2 | 7320338 | -2.2499154 | 18741 |
| Pkp1 | 3940070 | 3.0912378 | 18772 |
| Pla1a | 4210372 | 2.6096785 | 85031 |
| Pla2g4c | 2120021 | 6.8775816 | 232889 |
| Plac8 | 5860243 | 3.099563 | 231507 |
| Plat | 2760561 | 2.862345 |  |
| Plcb4 | 2490086 | -2.1990333 | 18798 |
| Plce1 | 940519 | -2.0666008 | 74055 |
| Plcg2 | 1940255 | 2.9876244 | 234779 |
| Plch2 | 6280564 | -2.344582 | 269615 |
| Pld4 | 5720255 | 3.2152364 | 104759 |
| Plek | 1820075 | 2.9457574 | 56193 |
| Plekha6 | 2490390 | -2.03026 | 240753 |
| Plekha6 | 2260021 | -2.1792865 | 240753 |
| Plk1 | 520427 | 2.1463373 | 18817 |
| Pllp | 430278 | -2.4693544 | 67801 |
| Plscr1 | 1340148 | 2.006532 | 22038 |
| Plvap | 3290239 | 2.9897866 | 84094 |
| Plxnc1 | 5050072 | 2.0558202 |  |
| Pmepa1 | 3420544 | 2.005141 | 65112 |
| Pon3 | 610400 | 2.6962228 | 269823 |
| Pou2af1 | 1690392 | 24.58675 | 18985 |
| Pou2f2 | 6370253 | 2.5381715 | 18987 |
| Pparg | 2320402 | -2.318546 | 19016 |
| Pparg | 6380066 | -2.5399091 | 19016 |
| Ppbp | 6580324 | 4.3432603 | 57349 |
| Ppbp | 110100 | 4.9263396 | 57349 |
| Ppfibp2 | 430403 | -4.1217737 | 19024 |
| Ppfibp2 | 4850097 | -2.8009808 | 19024 |
| Ppp1cb | 610202 | -2.8482077 | 19046 |
| Ppp1cb | 6580685 | -2.934524 |  |
| Ppp2r2b | 5550600 | -2.1412494 | 72930 |
| Ppyr1 | 2710278 | -2.418467 | 19065 |
| Prap1 | 7560138 | 2.4278772 | 22264 |
| Prc1 | 780475 | 3.3815875 | 233406 |
| Prg2 | 4280180 | 7.457961 | 19074 |
| Prg4 | 3370201 | 2.0023654 |  |
| Prom2 | 7160228 | -4.390929 | 192212 |
| Pros1 | 4670372 | 2.0889387 | 19128 |
| Prss22 | 650324 | 3.099673 | 70835 |
| Prss23 | 3830670 | -2.1786432 | 76453 |
| Prss32 | 4560133 | 4.153793 | 69814 |
| Prss35 | 4900296 | -2.6705627 | 244954 |
| Prss8 | 2650601 | -2.8157108 | 76560 |
| Prss8 | 6350291 | -3.562714 | 76560 |
| Psca | 3390192 | -3.031546 | 72373 |
| Psd2 | 630544 | -2.0285158 | 74002 |
| Psmb8 | 2570095 | 2.0519097 |  |
| Psmb8 | 2900136 | 2.0997937 | 16913 |
| Pstpip1 | 2360554 | 3.0029738 | 19200 |
| Ptch1 | 1300301 | -2.570792 |  |
| Pthlh | 7160102 | -2.4957435 | 19227 |
| Ptn | 830397 | -2.4269423 | 19242 |
| Ptplb | 4070554 | -2.1246264 | 70757 |
| Ptpn1 | 1430110 | 2.31092 | 19246 |
| Ptpn18 | 4050689 | 2.8307815 | 19253 |
| Ptpn22 | 5900593 | 3.799269 | 19260 |
| Ptpn6 | 4060240 | 4.033622 | 15170 |
| Ptpn6 | 2810541 | 3.12913 | 15170 |
| Ptprc | 1230541 | 2.8578095 | 19264 |
| Ptprcap | 6840360 | 2.4062722 | 19265 |
| Ptprr | 1940736 | -2.3965707 | 19279 |
| Ptprr | 2320475 | -2.0417614 | 19279 |
| Ptprz1 | 1690239 | -2.6006923 | 19283 |
| Pttg1ip | 6330309 | -2.055454 | 108705 |
| Ptx3 | 1740008 | 4.5215573 |  |
| Ptx3 | 5390136 | 3.4496267 | 19288 |
| Punc | 2450100 | -2.0174344 | 19289 |
| Pvrl3 | 5670465 | -2.2507927 | 58998 |
| Pygb | 3170110 | -2.0882983 | 110078 |
| Rab25 | 1580139 | -2.0030382 | 53868 |
| Rab25 | 630451 | -2.4925904 | 53868 |
| Rab27b | 1190706 | -2.735958 | 80718 |
| Rab27b | 2360167 | -2.90217 | 80718 |
| Rac2 | 4490243 | 5.1879053 | 19354 |
| Rai3 | 2690221 | 2.113871 |  |
| Rap1gap | 4730039 | 2.0644743 | 110351 |
| Rapgefl1 | 1010524 | -2.7622998 | 268480 |
| Rasd2 | 4590112 | -3.0249367 | 75141 |
| Rasef | 4290408 | -2.147705 | 242505 |
| Rasgrp1 | 5860296 | 4.6409826 | 19419 |
| Rasl11b | 150372 | -2.3732429 | 68939 |
| Rasl11b | 290341 | -2.3865175 | 68939 |
| Rassf2 | 5960487 | 3.0649855 | 215653 |
| Rassf5 | 6590044 | 2.4837153 | 54354 |
| Rbbp7 | 670711 | -2.0007026 | 245688 |
| Rbbp7 | 6590445 | -2.0252645 | 245688 |
| Rcsd1 | 6590296 | 2.3815246 | 226594 |
| Reep6 | 10364 | -2.8356826 | 70335 |
| Reg3g | 830619 | 16.993635 | 19695 |
| Retnla | 1110709 | 14.9562845 | 57262 |
| Retnlg | 7160711 | 22.75684 | 245195 |
| Rfc2 | 4730491 | 2.1203427 |  |
| Rftn1 | 5560349 | 2.2792177 | 76438 |
| Rfx2 | 990100 | -2.0017648 | 19725 |
| Rgs1 | 6840594 | 2.0185223 | 50778 |
| Rgs11 | 3290725 | -2.223164 |  |
| Rgs16 | 3890528 | 2.6982317 | 19734 |
| Rhof | 3780192 | 3.407798 | 23912 |
| Rhog | 2630703 | 2.1539414 | 56212 |
| Rims1 | 2070402 | -2.5124245 | 116837 |
| Rin3 | 1990204 | 3.0226033 | 217835 |
| Ripk4 | 1570598 | -2.0558937 | 72388 |
| Ripk4 | 3850086 | -2.1134744 | 72388 |
| Rnase4 | 5550369 | -3.4830868 |  |
| Rnase6 | 430703 | 4.0481772 | 78416 |
| Rrbp1 | 6840600 | 2.0067494 | 81910 |
| Rshl3 | 540255 | -2.0119545 | 212892 |
| Rsph1 | 4760066 | 2.8309686 | 22092 |
| Rtn4rl1 | 670333 | -2.0759888 | 237847 |
| S100a1 | 940338 | -2.5148 | 20193 |
| S100a3 | 6270228 | 2.2840579 | 20197 |
| S100a8 | 1190546 | 13.789071 | 20201 |
| S100a9 | 1980603 | 15.948917 | 20202 |
| Saa3 | 6400719 | 5.660957 | 20210 |
| Samsn1 | 2030543 | 2.6201613 | 67742 |
| Samsn1 | 6020400 | 4.7958436 | 67742 |
| Sbno2 | 6220037 | 2.2960403 | 216161 |
| Scamp5 | 7160372 | -2.6087267 | 56807 |
| Scara5 | 160377 | -2.9033813 | 71145 |
| Scin | 3800671 | 4.5383153 | 20259 |
| Scnn1b | 7210600 | -2.4329212 | 20277 |
| Scnn1g | 7100255 | -4.6934547 | 20278 |
| Scube2 | 6590041 | -3.907136 | 56788 |
| Sdf2l1 | 3360010 | 3.144467 | 64136 |
| Sdpr | 3180543 | -2.0819404 | 20324 |
| Sec11c | 5130497 | 2.5665724 | 66286 |
| Sel1h | 5870286 | 5.6360774 |  |
| Sel1l | 5260019 | 2.4822674 | 20338 |
| Sell | 5360707 | 6.5692353 | 20343 |
| Selp | 4290465 | 3.5070822 | 20344 |
| Selpl | 3800070 | 2.331371 |  |
| Selplg | 1070307 | 3.6989315 | 20345 |
| Sema4g | 5810253 | -2.65401 | 26456 |
| Serpina3g | 1050092 | 12.7639475 | 20715 |
| Serpina3h | 1450491 | 10.48744 | 546546 |
| Serpina3m | 110435 | 2.0346272 | 20717 |
| Serpina3n | 2450347 | 7.1375723 | 20716 |
| Serpinb1a | 4780328 | 2.449271 | 66222 |
| Serpine2 | 870309 | 3.3714118 |  |
| Sfrp1 | 4060398 | 2.382296 |  |
| Sfrp1 | 1070670 | 2.3247933 | 20377 |
| Sgpl1 | 6510348 | -2.283051 | 20397 |
| Sh2d2a | 4760575 | 2.7212589 | 27371 |
| Sh3bgrl2 | 5270220 | -2.0005178 | 212531 |
| Sh3bgrl2 | 7000451 | -2.003695 |  |
| Sh3gl2 | 1400689 | -8.205109 | 20404 |
| Sh3gl2 | 3870390 | -5.5079427 | 20404 |
| Sh3gl2 | 3840136 | -4.1984663 | 20404 |
| Sh3tc2 | 3180307 | -2.8471746 | 225608 |
| Sh3yl1 | 830360 | -2.632228 | 24057 |
| Shh | 2260450 | -2.2214599 | 20423 |
| Shh | 4560113 | -2.3784485 | 20423 |
| Sidt1 | 870156 | -2.9104137 | 320007 |
| Siglec1 | 4570458 | 3.8687696 | 20612 |
| Siglecg | 2850553 | 5.6279564 | 243958 |
| Sirpa | 3400646 | 2.0716312 | 19261 |
| Sirpb1 | 5090491 | 2.0500147 | 320832 |
| Sla | 110064 | 4.791504 | 20491 |
| Slamf9 | 7510291 | 2.351158 | 98365 |
| Slc12a8 | 4390136 | -2.2828667 | 171286 |
| Slc15a3 | 3290427 | 3.9683068 | 65221 |
| Slc16a6 | 3120427 | 5.7347784 | 104681 |
| Slc16a6 | 5860475 | 3.947614 | 104681 |
| Slc18a2 | 4480192 | 2.9583948 | 214084 |
| Slc24a3 | 4280131 | -2.144241 | 94249 |
| Slc26a4 | 6330279 | 2.0145428 | 23985 |
| Slc2a3 | 460327 | 2.0770714 | 20527 |
| Slc2a6 | 7400286 | 2.2081175 | 227659 |
| Slc34a2 | 3420162 | 2.8181489 | 20531 |
| Slc44a3 | 6590110 | -2.5287175 | 213603 |
| Slc45a3 | 5270504 | 2.3816445 | 212980 |
| Slc46a1 | 540475 | -2.1721673 | 52466 |
| Slc47a1 | 1260129 | -2.0575595 | 67473 |
| Slc6a2 | 5910717 | 2.4921203 | 20538 |
| Slc7a8 | 6550687 | 2.6003592 | 50934 |
| Slc7a8 | 3870681 | 2.7680984 | 50934 |
| Slco4a1 | 5820270 | 4.8989015 | 108115 |
| Slfn1 | 3520746 | 2.1907818 | 20555 |
| Slfn2 | 4120307 | 2.3920984 | 20556 |
| Slmap | 6900228 | -2.033716 | 83997 |
| Slmap | 6580239 | -3.8117294 | 83997 |
| Slpi | 2810487 | 82.310394 | 20568 |
| Smap2 | 2470224 | 2.0243735 | 69780 |
| Smox | 5050008 | 2.7956316 | 228608 |
| Smox | 1660367 | 3.2180777 | 228608 |
| Smtn | 5360626 | -2.6793098 | 29856 |
| Smtn | 620202 | -2.2574906 | 29856 |
| Snhg11 | 4220288 | -3.4378006 | 319317 |
| Snhg11 | 2480730 | -2.7679434 | 319317 |
| Snurf | 620209 | -2.7640405 | 84704 |
| Snurf | 3130246 | -3.0559046 | 84704 |
| Snx20 | 2810424 | 2.4669335 | 71607 |
| Socs2 | 4210129 | 2.8895574 | 216233 |
| Socs3 | 1570594 | 6.1126595 | 12702 |
| Sod3 | 2680070 | 2.087127 | 20657 |
| Sort1 | 2810468 | -2.2600698 | 20661 |
| Sort1 | 1470091 | -2.4269278 | 20661 |
| Sox21 | 270736 | 2.1943612 | 223227 |
| Sp100 | 6280221 | 2.183489 |  |
| Spcs3 | 5700537 | 3.30852 | 76687 |
| Spcs3 | 3780717 | 2.0617275 | 76687 |
| Spink6 | 1580201 | 2.1374915 | 433180 |
| Spint1 | 1740114 | -2.218345 | 20732 |
| Spock3 | 2100521 | -2.7546504 | 72902 |
| Spp1 | 1170139 | 7.3959155 | 20750 |
| Sprr2f | 2760669 | 40.330235 | 20760 |
| Sprr2g | 4220037 | 18.199324 | 20761 |
| Sprr2j | 520291 | 2.5335867 | 20764 |
| Sptlc3 | 60072 | -2.3775742 | 228677 |
| Srd5a1 | 1090474 | -4.171 |  |
| Srgn | 4610433 | 4.5798235 | 19073 |
| Srgn | 5490187 | 5.8213534 | 19073 |
| St6gal1 | 1430368 | 2.6746902 | 20440 |
| St6galnac2 | 6620161 | -2.2050562 | 20446 |
| St8sia4 | 3780187 | 2.0812042 | 20452 |
| Stab1 | 2320414 | 2.2442887 | 192187 |
| Stac | 270546 | -2.6914518 | 20840 |
| Stac2 | 610047 | 3.1794212 | 217154 |
| Stat4 | 5560066 | 2.945366 | 20849 |
| Stfa1 | 940717 | 17.251469 | 20861 |
| Stfa1 | 3360056 | 13.874907 | 20861 |
| Stk17b | 6270091 | 2.13697 | 98267 |
| Syk | 5890671 | 2.1269512 | 20963 |
| Syngr1 | 4850196 | -2.0700495 | 20972 |
| Synm | 1740364 | -2.3045855 | 233335 |
| Synm | 1410215 | -2.904953 | 233335 |
| Synpo | 5670634 | 2.1934385 |  |
| Synpo2 | 4900278 | -2.300132 | 118449 |
| Syt16 | 1510097 | -2.3246267 | 238266 |
| Sytl2 | 3710563 | -3.0891495 | 83671 |
| Tacr1 | 6250736 | 2.1241572 |  |
| Tagln2 | 6370204 | 2.747097 | 21346 |
| Tbc1d10c | 380672 | 2.1115773 | 108995 |
| Tbc1d9 | 2690243 | -2.1813672 | 71310 |
| Tbx2 | 670367 | -2.2771025 | 21385 |
| Tbxas1 | 5690164 | 2.6344898 | 21391 |
| Tcirg1 | 2570301 | 2.2113042 | 27060 |
| Tcirg1 | 4760324 | 2.2856467 | 27060 |
| Tcrb-V8.2 | 2510138 | 3.4538162 |  |
| Tef | 5270082 | -2.5343184 | 21685 |
| Tgfb1 | 5130139 | 2.2158 | 21803 |
| Tgfb1i1 | 3400441 | -2.538131 | 21804 |
| Tgfb1i1 | 2970692 | -2.352525 | 21804 |
| Tgm1 | 6270040 | 3.7076056 | 21816 |
| Thbs2 | 6180168 | -3.1262481 | 21826 |
| Thsd4 | 630608 | -3.3545268 | 207596 |
| Thsd4 | 1770086 | -3.995615 | 207596 |
| Timd4 | 3840367 | 2.9174926 | 276891 |
| Timeless | 160703 | 2.1814127 | 21853 |
| Timp1 | 4640215 | 7.8957353 |  |
| Timp1 | 160332 | 7.5304694 | 21857 |
| Tk1 | 7400142 | 2.6456497 | 21877 |
| Tlr1 | 6940719 | 2.2348576 | 21897 |
| Tmc7 | 5910408 | -2.0444257 | 209760 |
| Tmem159 | 4230661 | -2.6060076 | 233806 |
| Tmem159 | 3060056 | -2.4719777 | 233806 |
| Tmem176a | 3370021 | 2.2566698 | 66058 |
| Tmem184a | 430504 | -3.588815 | 231832 |
| Tmem2 | 2570142 | 2.1408672 | 83921 |
| Tmem45b | 7570053 | -3.07942 | 235135 |
| Tmem56 | 6280368 | -3.0433786 | 99887 |
| Tmem79 | 3120719 | -2.353768 | 71913 |
| Tmprss13 | 2060364 | -2.388852 | 214531 |
| Tmprss13 | 1300220 | -2.4064384 | 214531 |
| Tmprss2 | 4070279 | -2.5647328 | 50528 |
| Tnfrsf13b | 4280296 | 6.0187173 |  |
| Tnfrsf13c | 2710520 | 6.2739167 | 72049 |
| Tnfrsf17 | 2940482 | 2.9029412 | 21935 |
| Tnfrsf18 | 3180156 | 2.5393705 | 21936 |
| Tnfrsf4 | 2000747 | 2.1407511 | 22163 |
| Tnfsf11 | 2480255 | 2.8690712 | 21943 |
| Tnfsf13b | 6840059 | 3.38169 | 24099 |
| Tnfsf13b | 610661 | 3.7001743 |  |
| Tnk1 | 5490301 | -2.0764787 | 83813 |
| Top2a | 4250403 | 3.3705645 | 21973 |
| Tph1 | 130066 | 5.8566146 | 21990 |
| Tpm1 | 4220056 | -2.3689802 | 22003 |
| Tpm2 | 6370241 | -2.0017395 | 22004 |
| Tpmt | 2710020 | -2.114637 |  |
| Tpmt | 2320377 | -2.1757407 | 22017 |
| Tpmt | 2810711 | -2.0139441 |  |
| Tppp | 3420377 | -2.4040778 | 72948 |
| Tppp | 2140431 | -2.0861297 | 72948 |
| Tpsab1 | 5130553 | 3.7496643 | 17230 |
| Traf1 | 2510164 | 2.656445 |  |
| Traf1 | 3850463 | 2.8595045 | 22029 |
| Trem2 | 6770114 | 2.4006233 | 83433 |
| Trib2 | 540753 | 2.2415402 |  |
| Trim15 | 7200154 | 4.091115 | 69097 |
| Trim68 | 6650692 | -2.6192334 | 101700 |
| Tsku | 2030386 | -3.910375 | 244152 |
| Tspan18 | 2320035 | -2.1127658 | 241556 |
| Tspan2 | 4120360 | -2.4456685 | 70747 |
| Tspan32 | 830377 | 2.3330235 | 27027 |
| Tspan6 | 2370021 | -2.3637812 | 56496 |
| Tspan6 | 6940577 | -2.6358533 | 56496 |
| Ttr | 2680113 | -12.377898 | 22139 |
| Tyrobp | 450739 | 2.5486042 | 22177 |
| Ubd | 7040170 | 3.7582023 | 24108 |
| Ugcg | 4210440 | 2.4113507 |  |
| Ugcg | 1230564 | 2.4847622 | 22234 |
| Ugt1a10 | 6100739 | -2.6732843 | 394430 |
| Ugt1a10 | 6330372 | -2.8945785 | 394430 |
| Ugt1a10 | 2350131 | -3.1428385 | 394430 |
| Ugt1a10 | 2350730 | -3.0570815 | 394430 |
| Ugt1a6a | 3370669 | -5.2517695 | 94284 |
| Ugt1a6a | 1170349 | -2.8783293 | 94284 |
| Ugt1a6a | 3800446 | -6.2104197 | 94284 |
| Ugt1a6b | 3310138 | -6.7278185 | 394435 |
| Ugt1a7c | 650554 | -2.4953554 | 394432 |
| Ugt1a7c | 4060044 | -2.398266 | 394432 |
| Ugt2b34 | 4230358 | -2.1260293 | 100727 |
| Uhrf1 | 4560397 | 3.6743112 | 18140 |
| Unc13c | 5820538 | -2.2532873 | 208898 |
| Unc5b | 2030497 | -2.291345 | 107449 |
| Unc93b1 | 2100689 | 2.8122404 | 54445 |
| Unc93b1 | 5090403 | 2.6253374 | 54445 |
| Ung | 3130750 | 2.5754926 | 22256 |
| Upk1a | 1110762 | -5.7580657 | 109637 |
| Upk1a | 2970164 | -7.100268 | 109637 |
| Upk1b | 4880433 | -3.8104174 | 22268 |
| Upk2 | 2650554 | -5.255422 | 22269 |
| Upk3a | 630097 | -6.258444 | 22270 |
| Upk3b | 6270670 | -3.4453752 | 100647 |
| Upk3b | 4040243 | -3.2987187 | 100647 |
| Upk3b | 520333 | -3.918947 | 100647 |
| Upp1 | 3520594 | 2.2133186 | 22271 |
| Upp1 | 4120243 | 2.533262 | 22271 |
| Upp1 | 1050095 | 3.4477012 | 22271 |
| Vav1 | 5270608 | 3.688933 | 22324 |
| Vldlr | 4480639 | -2.0562866 | 22359 |
| Vldlr | 5900577 | -2.3653233 | 22359 |
| Vldlr | 4070201 | -2.1988225 | 22359 |
| Vpreb3 | 4900500 | 2.8721592 | 22364 |
| Vrk1 | 3610731 | 2.8646889 |  |
| Vsig2 | 3990593 | -3.6839561 | 57276 |
| Vsig2 | 5270347 | -3.2528176 | 57276 |
| Vwf | 7510414 | 2.8435109 | 22371 |
| Was | 5340180 | 4.045438 | 22376 |
| Wbscr27 | 7510474 | -2.7850382 | 79565 |
| Wfdc1 | 4540598 | -2.71193 |  |
| Wfdc15b | 110292 | -6.9544525 | 192201 |
| Wfdc15b | 2600017 | -4.6195498 | 192201 |
| Wnk2 | 6400370 | -2.750825 | 75607 |
| Wnt10a | 6510053 | 2.0616567 | 22409 |
| Wnt5a | 3420092 | -2.293312 |  |
| Wnt5a | 1410300 | -2.8058574 | 22418 |
| Wnt7b | 3400202 | -2.006699 | 22422 |
| Wwc1 | 6580008 | -2.0650804 | 211652 |
| Xdh | 5340577 | 2.102485 | 22436 |
| Zap70 | 2970544 | 2.0777926 | 22637 |
| Zc3h12d | 6510142 | 3.2157855 | 237256 |
| Zfp339 | 270682 | -2.3116975 |  |
| Zfp36 | 6220026 | 2.3029895 | 22695 |
| Zfp641 | 6620768 | -2.3425832 | 239652 |
| Zfpn1a1 | 5810048 | 2.510128 |  |
| mt-Nd4l | 3060209 | -3.4637294 |  |
| mtDNA_ND4L | 2680520 | -2.5038915 |  |
| mtDNA_ND5 | 3120520 | -2.1100945 |  |
| scl0001493.1_2 | 6280142 | -2.2936058 |  |
| scl0001849.1_2273 | 6370020 | 8.254661 |  |
| scl0001883.1_112 | 1690343 | -3.0257156 |  |
| scl0002540.1_6 | 6280403 | 2.8728082 |  |
| scl0003073.1_164 | 4640541 | -2.294529 |  |
| scl0003522.1_58 | 5860164 | -2.0449321 |  |
| scl0003547.1_6 | 7330521 | -2.9071758 |  |

**WEEK 5**

| Gene Symbol | Illumina Probe ID | Fold Change | Entrez Gene  ID |
| --- | --- | --- | --- |
| 0610007P14Rik | 4150367 | -2.1608117 | 58520 |
| 1200013B08Rik | 4260400 | 4.483157 | 74131 |
| 1300007L22Rik | 7610161 | -2.135102 |  |
| 1300013J15Rik | 6520022 | -5.120208 | 67473 |
| 1500003O03Rik | 6560593 | -2.1564639 |  |
| 1700124P09Rik | 5690129 | -2.2903743 | 76998 |
| 1700124P09Rik | 7100367 | -2.6401722 | 76998 |
| 1810011H11Rik | 7550458 | 2.2399986 | 69069 |
| 1810019D21Rik | 7560400 | -2.6274216 |  |
| 2010001M09Rik | 7210672 | 46.95255 | 69816 |
| 2010007H06Rik | 7650239 | 2.1220024 |  |
| 2200001I15Rik | 460682 | -2.3322172 | 69134 |
| 2310002D06Rik | 110300 | -3.5211678 | 69522 |
| 2310011E23Rik | 1780477 | 3.5135565 |  |
| 2310043J07Rik | 2760333 | 3.0280504 | 69665 |
| 2310058A03Rik | 5340091 | -2.0104506 |  |
| 2700060E02Rik | 5960066 | 2.2299256 | 68045 |
| 2900011O08Rik | 5670246 | -2.2922914 | 67254 |
| 4632417K18Rik | 1780279 | 3.198709 | 107373 |
| 4632417N05Rik | 5420332 | -2.4167106 | 74032 |
| 4733401O04Rik | 3130091 | 3.9393647 |  |
| 4833412C05Rik | 2000768 | -2.0961704 |  |
| 4930426D05Rik | 3360674 | -3.009177 |  |
| 4930431B09Rik | 6840270 | 8.058322 | 74645 |
| 4930452B06Rik | 290379 | -2.4896488 | 74430 |
| 4930553M18Rik | 5690348 | -2.1566353 |  |
| 5031414D18Rik | 1110575 | 2.6192217 | 271221 |
| 5133400G04Rik | 1510592 | -2.5154967 | 71242 |
| 5830405N20Rik | 2900307 | 2.0400977 | 67596 |
| 5830411E10Rik | 2140441 | -2.4753404 |  |
| 5830496L11Rik | 3190722 | 4.223439 |  |
| 6330407A03Rik | 110670 | 3.175289 |  |
| 6330442E10Rik | 5270167 | 2.1566353 | 268567 |
| 6330500D04Rik | 4200202 | 2.7961347 | 193385 |
| 6330500D04Rik | 1450097 | 2.2459779 |  |
| 8430408G22Rik | 5550161 | -2.7712176 | 213393 |
| 9030625A11Rik | 4200678 | -2.455756 |  |
| 9130213B05Rik | 2230026 | -2.2854207 | 231440 |
| 9530027K23Rik | 4560093 | -2.2398055 |  |
| 9530027K23Rik | 580255 | -2.0864804 |  |
| 9530051K01Rik | 3840187 | -2.0674987 |  |
| 9626100_224 | 1710136 | 2.1862612 |  |
| 9630015D15Rik | 2630086 | -2.573575 |  |
| 9930032O22Rik | 730020 | 4.4703846 | 320454 |
| A130001G05Rik | 2490735 | 2.529688 |  |
| A130010J15Rik | 3390672 | 3.8470814 | 319266 |
| A130024J05Rik | 6650470 | 2.1382399 |  |
| A130052C08Rik | 4180095 | 3.1751251 |  |
| A130082M07Rik | 4260463 | 2.6986961 | 279882 |
| A130090K04Rik | 6650626 | 2.5414062 |  |
| A130092J06Rik | 770669 | 2.4906726 | 241303 |
| A430084P05Rik | 1190477 | 2.1115298 | 327957 |
| A430084P05Rik | 5390088 | 2.7344239 | 327957 |
| A430107D22Rik | 670050 | 4.4928493 | 320484 |
| A530050E01Rik | 2320301 | 2.2240522 |  |
| A630006E02Rik | 7330131 | 4.332197 |  |
| A830080H07Rik | 2470746 | 2.4257452 |  |
| AA467197 | 7160047 | 2.258454 | 433470 |
| AA986860 | 5490703 | -2.1672447 | 212439 |
| AI324046 | 3120113 | 101.08464 |  |
| AI324046 | 4120458 | 28.949745 |  |
| AI324046 | 1770594 | 79.6645 |  |
| AI324046 | 3130280 | 35.175346 |  |
| AI427122 | 4890577 | 2.2695117 |  |
| AI467606 | 380670 | 2.4619017 | 101602 |
| AU019823 | 4890328 | -2.1842964 | 270156 |
| AU020206 | 1740609 | 2.909421 |  |
| Abcb1b | 4920129 | 2.1383784 | 18669 |
| Abcd3 | 2750064 | -2.035054 |  |
| Abi3 | 6450687 | 3.4257758 |  |
| Acaa1b | 4780528 | -2.731479 | 235674 |
| Acaa2 | 3310424 | -2.2393 | 52538 |
| Acaa2 | 5090414 | -2.3766692 |  |
| Acadl | 4220240 | -2.7308793 | 11363 |
| Ace | 5360674 | 2.034007 | 11421 |
| Acot1 | 520630 | -3.158899 | 26897 |
| Acot1 | 450356 | -3.8933203 | 26897 |
| Acpp | 2570280 | -2.1102076 | 56318 |
| Acsl1 | 2810047 | -2.352579 | 14081 |
| Acss1 | 6200719 | 2.144795 | 68738 |
| Acta1 | 2030450 | -4.601038 | 11459 |
| Acta2 | 3130136 | -2.1487758 |  |
| Actc1 | 1260669 | -3.623017 | 11464 |
| Actc1 | 6180554 | -3.3627274 | 11464 |
| Adcy2 | 1740541 | 2.0524762 | 210044 |
| Adcy7 | 5420082 | 2.1032255 |  |
| Adh1 | 110458 | -2.26356 | 11522 |
| Adh1 | 4850521 | -3.2688131 | 11522 |
| Aif1 | 1400672 | 2.2284393 | 11629 |
| Akna | 1070703 | 2.0216377 | 100182 |
| Aldh1a3 | 6420681 | 2.9387224 | 56847 |
| Alox12 | 4180717 | -3.2558818 | 11684 |
| Alox15 | 6580601 | 2.7487776 | 11687 |
| Alox5 | 5820286 | 2.4958968 | 11689 |
| Alox5ap | 3800372 | 2.360093 |  |
| Alox5ap | 5270279 | 2.1138368 | 11690 |
| Als2cl | 6380112 | -2.15548 | 235633 |
| Als2cr4 | 4050059 | -2.1790702 | 381259 |
| Als2cr4 | 7100215 | -2.0693388 | 381259 |
| Anxa8 | 2690215 | 2.0513997 | 11752 |
| Anxa8 | 4180239 | 2.0670242 | 11752 |
| Aplnr | 7510653 | 2.1193714 | 23796 |
| Apob48r | 4220189 | 2.2079623 | 171504 |
| Apobec1 | 1110088 | 2.3081324 | 11810 |
| Apoc1 | 1710066 | -2.5464132 | 11812 |
| Apoc1 | 5820600 | -2.9406142 | 11812 |
| Apoe | 7210687 | 2.2685356 | 11816 |
| Aqp9 | 5810452 | 3.1540377 | 64008 |
| Arg1 | 1190445 | 7.4164925 | 11846 |
| Arhgap30 | 4250544 | 5.8035264 | 226652 |
| Arhgap4 | 7160474 | 5.354049 | 171207 |
| Arhgap9 | 5870470 | 2.502651 | 216445 |
| Arhgdib | 1090180 | 2.7081082 | 11857 |
| Arhgdig | 4570239 | -2.0215163 | 14570 |
| Arhgef1 | 6960608 | 2.2469125 |  |
| Arrb2 | 1110451 | 2.6866033 | 216869 |
| Asah1 | 130121 | -2.0263064 | 11886 |
| Asah3l | 1470386 | -2.7691293 | 230379 |
| Atp2a3 | 2570672 | 3.8213696 | 53313 |
| Atp2a3 | 60288 | 3.6937664 | 53313 |
| Atp6ap2 | 5570068 | -2.186623 | 70495 |
| Atrn | 3890184 | -2.4475543 | 11990 |
| B230114H05Rik | 2970440 | -2.3567495 |  |
| B4galnt1 | 270711 | 3.8874013 | 14421 |
| BC006779 | 450706 | 2.183887 | 229003 |
| BC006779 | 6420170 | 2.183459 |  |
| BC100530 | 7150100 | 4.2353554 | 100034684 |
| Bank1 | 4830082 | 4.232807 | 242248 |
| Bcas1 | 6560347 | -3.3216858 | 76960 |
| Bcl11a | 460692 | 2.6373103 | 14025 |
| Bcl11a | 1570022 | 3.6592915 |  |
| Bcl11b | 6110008 | 2.0456805 | 58208 |
| Bcl2a1b | 130097 | 3.6692867 | 12045 |
| Bcl2a1d | 7040202 | 4.891754 |  |
| Bcl2l14 | 60452 | -2.1391375 | 66813 |
| Bcl3 | 4070020 | 2.0453994 | 12051 |
| Bex4 | 2480523 | -2.271891 | 406217 |
| Bglap-rs1 | 460402 | -2.6424112 | 12095 |
| Bglap-rs1 | 3450521 | -2.2618577 | 12095 |
| Bglap1 | 4540537 | -2.04142 | 12096 |
| Bglap2 | 4670497 | -3.2537148 | 12097 |
| Bhlhb2 | 1230341 | 2.0570292 | 20893 |
| Bhlhb8 | 2750068 | 2.2688358 | 17341 |
| Bhlhb8 | 2490138 | 3.054284 | 17341 |
| Bhmt2 | 610576 | -2.914428 | 64918 |
| Birc3 | 870008 | 2.2845173 | 11796 |
| Blk | 3290279 | 8.113916 | 12143 |
| Blk | 3140491 | 3.7077887 | 12143 |
| Blnk | 6840424 | 2.0806332 | 17060 |
| Bmp3 | 940121 | -2.332957 | 110075 |
| Btk | 5050343 | 2.9406996 | 12229 |
| Btla | 6980672 | 2.0016315 | 208154 |
| C030014I23Rik | 5670750 | -2.421575 |  |
| C030025P15Rik | 6130095 | -2.388539 |  |
| C130078N17Rik | 5260020 | 2.4354975 |  |
| C130090K23Rik | 1710204 | 6.698992 | 231293 |
| C130090K23Rik | 5900307 | 2.0760489 | 231293 |
| C1qb | 580332 | 2.8818326 | 12260 |
| C1qc | 3710170 | 2.0028963 | 12262 |
| C330006A16Rik | 7200189 | -2.5414302 |  |
| C4a | 60368 | 4.650926 | 625018 |
| C4a | 130348 | 4.8714495 | 625018 |
| C4b | 7510243 | 5.514197 | 12268 |
| C6 | 6220553 | 3.0534286 | 12274 |
| C730013O11Rik | 520504 | 2.2477605 |  |
| C920004C08Rik | 4540477 | 2.48906 |  |
| Cald1 | 4210431 | -2.2031596 | 109624 |
| Calu | 1410091 | -2.2148643 | 12321 |
| Camkk1 | 7610424 | -2.1174133 | 55984 |
| Card11 | 7330731 | 2.554497 | 108723 |
| Casp1 | 1940722 | 2.230287 | 12362 |
| Casp4 | 4290180 | 2.0897517 | 12363 |
| Casp4 | 5290017 | 2.0575116 | 12363 |
| Cav2 | 4480576 | -2.475388 | 12390 |
| Ccbl1 | 2850746 | -2.1052747 | 70266 |
| Ccdc88b | 1470079 | 3.8914146 | 78317 |
| Ccl11 | 830273 | 2.3479474 | 20292 |
| Ccl11 | 6060520 | 3.8764584 | 20292 |
| Ccl19 | 4220717 | 2.3070464 | 24047 |
| Ccl21a | 1710612 | 4.0063767 | 18829 |
| Ccl21c | 4230315 | 4.4602447 | 65956 |
| Ccl21c | 6370475 | 3.8170295 |  |
| Ccl5 | 1690768 | 2.605253 | 20304 |
| Ccl6 | 7040717 | 3.072395 |  |
| Ccl9 | 7050538 | 2.8782942 | 20308 |
| Ccnb1 | 7550156 | 2.0568004 | 268697 |
| Ccr6 | 4590639 | 3.1465702 | 12458 |
| Ccr6 | 1010132 | 9.127765 | 12458 |
| Ccr7 | 2570291 | 2.1566484 | 12775 |
| Cd19 | 6280037 | 3.708735 | 12478 |
| Cd19 | 6480746 | 4.1166744 | 12478 |
| Cd2 | 60221 | 2.6205995 | 12481 |
| Cd209d | 6250497 | 2.6813672 | 170779 |
| Cd22 | 5490608 | 2.8372836 | 12483 |
| Cd22 | 940504 | 5.0562267 | 12483 |
| Cd27 | 4810204 | 2.7689888 | 21940 |
| Cd37 | 5050431 | 4.00045 | 12493 |
| Cd3d | 610324 | 3.1722794 |  |
| Cd3e | 6590239 | 3.0284846 | 12501 |
| Cd3g | 940747 | 3.652328 | 12502 |
| Cd40 | 4760241 | 3.1159534 | 21939 |
| Cd44 | 4880138 | 2.1032329 | 12505 |
| Cd52 | 1170181 | 6.9168053 |  |
| Cd52 | 6290768 | 11.382407 | 23833 |
| Cd53 | 6560301 | 4.5204883 | 12508 |
| Cd6 | 1440681 | 2.4561226 | 12511 |
| Cd6 | 1170246 | 2.173714 |  |
| Cd69 | 540647 | 3.8143902 | 12515 |
| Cd72 | 5700528 | 8.387469 | 12517 |
| Cd74 | 6770195 | 5.056793 | 16149 |
| Cd74 | 6130072 | 3.2003045 | 16149 |
| Cd74 | 3360338 | 3.7833517 | 16149 |
| Cd79b | 2060373 | 18.031347 | 15985 |
| Cd79b | 2060543 | 8.588804 | 15985 |
| Cd83 | 7150377 | 4.8720236 | 12522 |
| Cd84 | 2450753 | 2.9441776 | 12523 |
| Cd84 | 4150164 | 2.2027478 |  |
| Cd86 | 60681 | 2.241998 | 12524 |
| Cd86 | 2850010 | 2.2214339 | 12524 |
| Cd8b1 | 1690079 | 3.0935419 | 12526 |
| Cdc42ep3 | 4150739 | -2.1897 | 260409 |
| Cds1 | 6270528 | -2.2097552 | 74596 |
| Cdt1 | 1050706 | 2.03837 | 67177 |
| Cebpe | 1340634 | 3.8317614 | 110794 |
| Cebpe | 6330634 | 2.069274 | 110794 |
| Cebpe | 3400608 | 2.1295083 | 110794 |
| Cenpa | 7200519 | 2.7190092 | 12615 |
| Centd3 | 6020255 | 2.1347768 | 106952 |
| Chi3l3 | 2570240 | 7.8552184 | 12655 |
| Chi3l3 | 3870242 | 6.131641 |  |
| Chi3l4 | 2600180 | 5.642766 | 104183 |
| Chmp2a | 540156 | -2.8744445 | 68953 |
| Chst2 | 2060544 | -2.0156894 | 54371 |
| Chst3 | 2810315 | 2.4658968 | 53374 |
| Chst3 | 4050437 | 3.674039 | 53374 |
| Ciita | 2630463 | 3.872734 | 12265 |
| Clca3 | 2690349 | 2.4435844 |  |
| Cldn10 | 3870753 | -2.140554 | 58187 |
| Cldn23 | 2350398 | -2.0195158 | 71908 |
| Clec2d | 7570181 | 2.1419482 | 93694 |
| Clec4a1 | 2760259 | 2.3795125 | 269799 |
| Clec4a3 | 4810154 | 2.1123786 | 73149 |
| Clec4b1 | 870112 | 2.2008312 | 69810 |
| Clec4n | 5360056 | 2.0665135 | 56620 |
| Clec7a | 1450333 | 2.9698176 | 56644 |
| Cma1 | 2650056 | 5.0511346 | 17228 |
| Cma1 | 4210039 | 5.9588284 | 17228 |
| Cma2 | 6550086 | 4.3865013 | 545055 |
| Cma2 | 2940102 | 13.810948 | 545055 |
| Cmah | 3140441 | 2.0930216 |  |
| Cnp | 6940079 | 2.1416602 | 12799 |
| Cnr2 | 7610743 | 3.0912619 |  |
| Cnr2 | 6590196 | 2.9660802 | 12802 |
| Cntnap2 | 620703 | -2.3061976 | 66797 |
| Col17a1 | 2510064 | 3.4872892 | 12821 |
| Col17a1 | 2140148 | 4.101338 | 12821 |
| Col17a1 | 6770369 | 2.5895402 | 12821 |
| Col3a1 | 5340692 | -3.8617742 | 12825 |
| Col7a1 | 3290209 | 2.1673672 | 12836 |
| Coro1a | 4290661 | 8.491979 | 12721 |
| Coro1a | 2480725 | 4.127458 | 12721 |
| Coro1a | 1500463 | 8.380488 | 12721 |
| Coro1a | 270661 | 5.8226995 | 12721 |
| Cotl1 | 990438 | 2.2989345 | 72042 |
| Cotl1 | 3120703 | 2.530207 |  |
| Cox6a2 | 2470356 | 2.098785 | 12862 |
| Cpa3 | 3420672 | 3.6805038 | 12873 |
| Cpa3 | 1410739 | 9.266803 | 12873 |
| Cpa3 | 7650487 | 7.3125215 | 12873 |
| Cr2 | 3190288 | 3.0430915 | 12902 |
| Creld2 | 4860079 | 5.1121335 | 76737 |
| Creld2 | 290685 | 4.713917 | 76737 |
| Crlf1 | 3780450 | 2.3461654 | 12931 |
| Crlf3 | 1690017 | 2.0252972 | 54394 |
| Csf1r | 3400369 | 2.1535523 | 12978 |
| Csf2rb2 | 5820528 | 4.897833 | 12984 |
| Csgalnact1 | 5090619 | 2.2974274 | 234356 |
| Csprs | 6520392 | 3.7220602 | 114564 |
| Csrp1 | 4780066 | -2.5255227 | 13007 |
| Ctla4 | 5560288 | 2.2687898 | 12477 |
| Ctnnal1 | 2320168 | -2.3569524 | 54366 |
| Ctps2 | 4060291 | -2.125032 | 55936 |
| Ctss | 4200646 | 2.6021028 | 13040 |
| Cx3cl1 | 7650746 | 2.09847 | 20312 |
| Cx3cr1 | 6620376 | 2.5416899 |  |
| Cxcl12 | 1030025 | 2.10673 | 20315 |
| Cxcl13 | 3060040 | 12.757931 |  |
| Cxcl15 | 520403 | 3.3119714 | 20309 |
| Cxcr4 | 160392 | 4.2022967 |  |
| Cxcr5 | 1470408 | 3.1863582 | 12145 |
| Cxcr5 | 1050632 | 2.2799218 | 12145 |
| Cxcr5 | 50091 | 5.859259 | 12145 |
| Cyba | 830369 | 2.4826207 | 13057 |
| Cyfip2 | 7550059 | 2.0293183 | 76884 |
| Cyp3a57 | 7160017 | -3.1023488 | 622127 |
| Cyp4f15 | 6040392 | -4.069336 | 106648 |
| Cyp4f18 | 2120392 | 4.483878 | 72054 |
| Cyp7b1 | 360187 | 2.0963557 | 13123 |
| Cyth4 | 7160044 | 3.2085457 | 72318 |
| Cytip | 6650070 | 10.518927 | 227929 |
| D11Bwg0517e | 5700669 | -2.3085635 | 52897 |
| D11Bwg0517e | 5720601 | -2.193697 | 52897 |
| D130032J17Rik | 1850474 | 2.5517943 |  |
| D17H6S56E-5 | 3170494 | 5.125556 | 110956 |
| D230007K08Rik | 2940441 | 2.4515462 | 268857 |
| D4Bwg0951e | 3310025 | -2.1615021 | 52829 |
| D6Mit97 | 1850452 | 95.40816 |  |
| Dci | 5390128 | -2.1000535 | 13177 |
| Decr2 | 5910349 | -2.7675788 |  |
| Decr2 | 3310161 | -3.0373478 |  |
| Dennd1c | 6220044 | 5.5002356 | 70785 |
| Dennd3 | 6330575 | 2.0206451 | 105841 |
| Derl3 | 7400719 | 3.224694 | 70377 |
| Des | 1780411 | -2.4884298 | 13346 |
| Dlk2 | 6330672 | -2.4263988 | 106565 |
| Dm15 | 4290066 | -2.0254571 |  |
| Dnajc6 | 6940537 | -2.1470523 |  |
| Dnajc6 | 2070594 | -2.3885188 | 72685 |
| Dnase1l2 | 1010168 | -2.2466295 | 66705 |
| Dnase1l3 | 6520193 | 2.6693609 |  |
| Dnmt3l | 4280100 | -2.063068 | 54427 |
| Dnmt3l | 460746 | -2.1412807 | 54427 |
| Dock2 | 1580551 | 2.1753833 |  |
| Dok3 | 610291 | 6.016473 | 27261 |
| Dtna | 1260025 | -2.5139503 | 13527 |
| Dusp16 | 6220279 | -2.0459375 | 70686 |
| E130302P19Rik | 6650446 | 3.0856364 |  |
| E330020G21Rik | 5270687 | 2.2708817 |  |
| E430021P16Rik | 6040072 | 4.7070274 |  |
| EG433144 | 7040646 | -2.0850103 | 433144 |
| EG433229 | 240259 | -2.9093668 | 433229 |
| EG665033 | 6560711 | 4.1006527 | 665033 |
| ENSMUSG00000068790 | 1990021 | -2.2279737 | 545007 |
| Ear11 | 1980095 | 23.4283 | 93726 |
| Ear12 | 4540543 | 2.0592787 | 503845 |
| Ear2 | 3130681 | 5.9281263 | 13587 |
| Ear2 | 4070148 | 3.624157 | 13587 |
| Ear2 | 5870605 | 3.5995605 | 13587 |
| Ear3 | 6860674 | 2.5625043 | 53876 |
| Ear3 | 2140035 | 2.0656257 | 53876 |
| Ear4 | 6250546 | 6.992636 | 53877 |
| Ebi2 | 2600377 | 2.4236531 |  |
| Ebpl | 5810722 | -2.1063185 | 68177 |
| Ebpl | 110630 | -2.3127303 | 68177 |
| Edem1 | 4120689 | 2.4337182 | 192193 |
| Edem2 | 5220639 | 2.7383418 | 108687 |
| Edem2 | 2490333 | 2.4812558 | 108687 |
| Edem2 | 1070678 | 2.849452 | 108687 |
| Efs | 4610338 | -2.004809 | 13644 |
| Eif4a2 | 4850747 | -2.0672843 | 13682 |
| Ela1 | 5290438 | -2.4300923 | 109901 |
| Elf5 | 6040196 | -2.1803594 | 13711 |
| Elf5 | 3460424 | -3.27102 | 13711 |
| Ell3 | 6590368 | 2.1046371 | 269344 |
| Emb | 270341 | 3.1933362 | 13723 |
| Emid1 | 1430079 | -2.5234737 | 140703 |
| Emr1 | 1030411 | 2.9268067 | 13733 |
| Emr1 | 2710110 | 2.4277074 | 13733 |
| Eno3 | 3830601 | -3.0476484 | 13808 |
| Enpp5 | 4850082 | -2.0437248 | 83965 |
| Ero1lb | 1980470 | 2.7206645 | 67475 |
| Ets1 | 6020398 | 2.8951125 | 23871 |
| Evi2a | 6760255 | 2.447298 | 14017 |
| F5 | 4610138 | 3.8019462 | 14067 |
| Fa2h | 2810706 | -2.1073666 | 338521 |
| Faim3 | 1990546 | 21.425013 | 69169 |
| Fam169b | 3400097 | 2.3033154 | 434197 |
| Fbxl22 | 6860475 | -2.2319956 | 74165 |
| Fcer1a | 5290026 | 10.13366 | 14125 |
| Fcer1a | 3420082 | 7.0834746 |  |
| Fcer2a | 6520630 | 2.085319 | 14128 |
| Fcer2a | 2750706 | 4.3724456 | 14128 |
| Fcgr2b | 5700220 | 2.6674073 | 14130 |
| Fcgr2b | 1110326 | 3.734189 | 14130 |
| Fcgr3 | 3830678 | 2.0459528 | 14131 |
| Fcgr4 | 830632 | 5.4039955 | 246256 |
| Fcho1 | 4390239 | 2.84243 | 74015 |
| Fchsd2 | 5260079 | 2.0871704 | 207278 |
| Fcrla | 5220176 | 2.4764774 | 98752 |
| Fcrla | 1260477 | 8.592654 | 98752 |
| Fcrla | 4050093 | 8.619095 | 98752 |
| Fcrla | 670703 | 9.793349 | 98752 |
| Fen1 | 3460037 | 2.1182487 | 14156 |
| Fermt3 | 160259 | 2.754856 | 108101 |
| Fes | 5420739 | 2.4364908 | 14159 |
| Fgd2 | 5900440 | 2.4677374 | 26382 |
| Fgr | 5690041 | 2.597275 | 14191 |
| Fkbp2 | 1050082 | 2.0421932 | 14227 |
| Flrt3 | 4390470 | -2.1059318 | 71436 |
| Fmnl3 | 2970528 | 2.7099545 | 22379 |
| Fmo5 | 4560300 | -4.2766585 | 14263 |
| Fn1 | 730427 | -2.0201945 | 14268 |
| Fos | 510368 | -6.6537175 | 14281 |
| Foxa1 | 430670 | -2.111968 | 15375 |
| Foxq1 | 1850487 | -2.0549402 | 15220 |
| Fpr2 | 7650048 | 2.0849137 | 14289 |
| Galntl1 | 2690373 | -3.0363405 | 108760 |
| Gata3 | 5900070 | -2.314748 | 14462 |
| Gimap1 | 150433 | 3.6913602 | 16205 |
| Gimap6 | 6650477 | 2.9411712 | 231931 |
| Gimap7 | 670204 | 3.1245492 | 231932 |
| Gimap8 | 4010402 | 2.7546322 | 243374 |
| Gjb2 | 6280382 | 2.18416 | 14619 |
| Gldc | 6590097 | -2.2157533 | 104174 |
| Gldc | 5560468 | -2.9029763 | 104174 |
| Glipr1 | 2810685 | 2.3352795 | 73690 |
| Glycam1 | 5050059 | 13.106938 | 14663 |
| Gm459 | 5720037 | 36.163242 | 243451 |
| Gm566 | 3800746 | 3.2487748 | 229672 |
| Gmfg | 7050450 | 3.0277107 | 63986 |
| Gmfg | 1070091 | 3.2447703 | 63986 |
| Gmip | 3400114 | 2.19541 | 78816 |
| Gp49a | 5820739 | 2.6964636 |  |
| Gpam | 2470427 | -2.046791 | 14732 |
| Gpld1 | 6940064 | -2.414493 | 14756 |
| Gpld1 | 6420368 | -2.3535886 | 14756 |
| Gpr113 | 5720373 | 2.209652 | 381628 |
| Gpr114 | 4890092 | 2.719844 | 382045 |
| Gpr171 | 2370039 | 6.1039076 | 229323 |
| Gpr176 | 1770634 | 2.666917 | 381413 |
| Gpr18 | 2750543 | 4.9273615 | 110168 |
| Gpr65 | 5670687 | 3.3788013 | 14744 |
| Gpx2 | 2680291 | 2.3589876 | 14776 |
| Grhl3 | 2340400 | -2.0201554 | 230824 |
| Grhl3 | 6280020 | -2.293616 | 230824 |
| Gsdmc3 | 5870520 | 2.690528 | 270328 |
| Gsdmc3 | 4210475 | 2.619216 | 270328 |
| Gsn | 4260709 | -2.0474284 | 227753 |
| Gsta3 | 3780193 | -2.9065447 | 14859 |
| Gstm1 | 3850129 | -2.155638 | 14862 |
| Gstm6 | 2940541 | -4.536658 |  |
| Gstt3 | 2350324 | -2.3941836 | 103140 |
| Gtl2 | 6940750 | -3.471097 |  |
| Gvin1 | 450735 | 2.5600524 | 74558 |
| H2-Aa | 650707 | 2.6717372 | 14960 |
| H2-Ab1 | 5860435 | 3.9527905 | 14961 |
| H2-DMa | 870154 | 3.4492333 | 14998 |
| H2-DMb1 | 4900754 | 4.0271354 | 14999 |
| H2-DMb2 | 3450482 | 4.533304 | 15000 |
| H2-DMb2 | 7040731 | 6.354906 | 15000 |
| H2-Ea | 3450528 | 3.225776 | 14968 |
| H2-Eb1 | 520072 | 4.4162374 | 14969 |
| H2-M2 | 6560747 | 2.2943108 | 14990 |
| H2-Oa | 730075 | 3.3616753 | 15001 |
| H2-Oa | 5050739 | 5.302696 |  |
| H3f3b | 4900450 | 2.3317027 | 15081 |
| Haao | 6330164 | 2.3786108 | 107766 |
| Haao | 1740164 | 2.7377262 | 107766 |
| Hadhb | 5290500 | -2.0673134 | 231086 |
| Hcls1 | 7570348 | 2.3927224 | 15163 |
| Hcst | 1510497 | 2.0705087 | 23900 |
| Herc1 | 5420138 | -2.329014 | 235439 |
| Hes1 | 4230356 | -2.0333498 | 15205 |
| Hgf | 5570079 | 2.2967498 |  |
| Hist1h2ad | 7160253 | 3.1425686 | 319165 |
| Hist1h2ad | 3520717 | 3.0987847 | 319165 |
| Hist1h2af | 4250711 | 3.6417336 | 319173 |
| Hist1h2ah | 1470341 | 2.6310844 | 319168 |
| Hist1h2ah | 670739 | 3.010829 | 319168 |
| Hist1h2ai | 5490193 | 3.2006252 | 319191 |
| Hist1h2ak | 3130609 | 2.7155182 | 319169 |
| Hist1h2an | 4610129 | 3.3929663 | 319170 |
| Hist1h2ao | 6510253 | 3.7458744 | 319171 |
| Hist1h4f | 6380377 | 2.0083418 | 319157 |
| Hmha1 | 4850594 | 3.9785972 | 70719 |
| Hoxb2 | 360025 | -2.105576 |  |
| Hrmt1l2 | 6480630 | -2.011883 |  |
| Hsd17b2 | 7320753 | -2.094588 | 15486 |
| Hspa8 | 2030593 | -2.204082 | 15481 |
| Hspb1 | 5670722 | -2.2015796 | 15507 |
| Hspb6 | 4040386 | -2.470658 | 243912 |
| Hspd1 | 5670192 | -2.0611367 | 15510 |
| Hvcn1 | 7510008 | 3.8101482 | 74096 |
| Hvcn1 | 620646 | 4.2713475 | 74096 |
| IGHA_J00475$V00785_Ig_heavy_constant_alpha_135 | 430053 | 30.480402 |  |
| IGHG1_J00453$V00793_Ig_heavy_constant_gamma_1_792 | 6420458 | 157.94756 |  |
| IGHV10S3_AF064446_Ig_heavy_variable_10S3_9 | 670674 | 27.0149 |  |
| IGHV12S1_M22439_Ig_heavy_variable_12S1_339 | 6520711 | 24.068281 |  |
| IGHV1S113_L33954_Ig_heavy_variable_1S113_110 | 670706 | 32.27733 |  |
| IGHV1S114_L33955_Ig_heavy_variable_1S114_205 | 770377 | 3.042173 |  |
| IGHV1S119_L33961_Ig_heavy_variable_1S119_14 | 2000465 | 43.49343 |  |
| IGHV1S120_AF025443_Ig_heavy_variable_1S120_8 | 4150180 | 24.473963 |  |
| IGHV1S124_AF025449_Ig_heavy_variable_1S124_11 | 20397 | 8.9959345 |  |
| IGHV1S133_AF304553_Ig_heavy_variable_1S133_89 | 1770241 | 6.4453306 |  |
| IGHV1S135_AF304556_Ig_heavy_variable_1S135_43 | 5870296 | 10.407991 |  |
| IGHV1S14_K00707$X00161_Ig_heavy_variable_1S14_164 | 4260717 | 13.8386755 |  |
| IGHV1S28_X02460_Ig_heavy_variable_1S28_13 | 1440373 | 38.696438 |  |
| IGHV1S30_X02462_Ig_heavy_variable_1S30_12 | 1240202 | 25.46934 |  |
| IGHV1S31_X02463_Ig_heavy_variable_1S31_40 | 1660239 | 8.765559 |  |
| IGHV1S34_X02467_Ig_heavy_variable_1S34_71 | 3870139 | 28.371128 |  |
| IGHV1S35_M12376_Ig_heavy_variable_1S35_13 | 1470286 | 18.477814 |  |
| IGHV1S36_M13788_Ig_heavy_variable_1S36_40 | 1510768 | 11.010434 |  |
| IGHV1S41_X06868_Ig_heavy_variable_1S41_72 | 6900348 | 3.1052887 |  |
| IGHV1S52_M34982_Ig_heavy_variable_1S52_158 | 4920377 | 4.8006477 |  |
| IGHV1S59_L17134_Ig_heavy_variable_1S59_150 | 2230327 | 15.679157 |  |
| IGHV2S1_V00767$J00492_Ig_heavy_variable_2S1_5 | 6580504 | 67.309944 |  |
| IGHV5S18_AF290972_Ig_heavy_variable_5S18_125 | 3610576 | 84.78628 |  |
| IGHV8S6_U23021_Ig_heavy_variable_8S6_61 | 4890475 | 79.06712 |  |
| IGHV8S7_U23022_Ig_heavy_variable_8S7_163 | 3390743 | 66.2758 |  |
| IGHV9S5_L14364_Ig_heavy_variable_9S5_82 | 2100154 | 3.118718 |  |
| IGKV1-88_AJ231206_Ig_kappa_variable_1-88_289 | 5870008 | 26.683048 |  |
| IGKV1-99_AJ231207_Ig_kappa_variable_1-99_1 | 5910129 | 2.0418832 |  |
| IGKV2-137_AJ231263_Ig_kappa_variable_2-137_15 | 70619 | 47.890526 |  |
| IGKV3-2_X16954_Ig_kappa_variable_3-2_18 | 4040079 | 60.874268 |  |
| IGKV4-53_AJ231231_Ig_kappa_variable_4-53_12 | 5720750 | 11.659892 |  |
| IGKV4-71_AJ231218_Ig_kappa_variable_4-71_20 | 2070300 | 124.76978 |  |
| IGKV4-73_AJ231216_Ig_kappa_variable_4-73_18 | 3850528 | 204.13853 |  |
| IGKV4-80_AJ231213_Ig_kappa_variable_4-80_91 | 520524 | 12.554639 |  |
| IGKV4-91_AJ231229_Ig_kappa_variable_4-91_29 | 1170079 | 44.93452 |  |
| IGKV6-13_J00569_Ig_kappa_variable_6-13_23 | 5130025 | 9.61457 |  |
| IGKV8-31_AJ235957_Ig_kappa_variable_8-31_3 | 610592 | 40.93271 |  |
| IGKV9-120_V00804$J00566_Ig_kappa_variable_9-120_12 | 2680367 | 90.434586 |  |
| IGKV9-128_AJ231245_Ig_kappa_variable_9-128_15 | 5570168 | 31.104433 |  |
| IGLC2_J00595_Ig_lambda_constant_2_14 | 130162 | 39.374313 |  |
| Icam1 | 1450095 | 2.0261853 | 15894 |
| Id1 | 6200692 | -2.0630474 | 15901 |
| Ifi30 | 4290709 | 2.5644572 | 65972 |
| Ifi30 | 4730367 | 3.165506 | 65972 |
| Ifit3 | 7510020 | 2.252087 | 15959 |
| Ifitm1 | 4010019 | 2.7920477 | 68713 |
| Ifitm1 | 5820068 | 2.6417131 | 68713 |
| Igf1 | 2000647 | 2.4434085 | 16000 |
| Igfbp2 | 1230240 | -11.042671 | 16008 |
| Igfbp2 | 580364 | -9.738783 | 16008 |
| Igfbp4 | 5080435 | 3.5811205 |  |
| Igfbp5 | 5080292 | 2.0738626 | 16011 |
| Igh-4 | 5960402 | 158.36989 |  |
| Igh-6 | 5340278 | 30.479435 |  |
| Igh-6 | 1010138 | 37.544563 |  |
| Igh-V11 | 3800246 | 26.873346 |  |
| Igh-VJ558 | 6380762 | 90.69887 | 16061 |
| Igh-VJ558 | 240242 | 136.02325 |  |
| Igh-VS107 | 3120301 | 17.189884 |  |
| Ighg | 1110253 | 129.19917 | 380794 |
| Ighg | 6660156 | 66.86578 | 380794 |
| Igj | 4250162 | 8.097459 | 16069 |
| Igk-V1 | 5310347 | 3.7753217 |  |
| Igk-V33 | 7510066 | 34.49881 |  |
| Igk-V38 | 830576 | 15.811926 |  |
| Igk-V38 | 5340196 | 13.713097 |  |
| Igk-V5 | 2030372 | 67.839966 | 381777 |
| Igk-V5 | 4640113 | 120.09994 | 381777 |
| Igl-V1 | 5080091 | 116.70581 |  |
| Igl-V1 | 4730180 | 210.35495 |  |
| Ikzf1 | 2070070 | 2.4202867 | 22778 |
| Il10ra | 3990474 | 3.070781 | 16154 |
| Il13ra2 | 5820446 | 3.5579536 | 16165 |
| Il16 | 2760427 | 4.154318 | 16170 |
| Il17re | 50706 | -2.4805439 | 57890 |
| Il17re | 7100020 | -2.485747 | 57890 |
| Il1r2 | 3420139 | 2.3459072 | 16178 |
| Il1rl1 | 3870291 | 8.39383 | 17082 |
| Il1rn | 3390131 | 2.2088492 | 16181 |
| Il27ra | 6620446 | 2.5383174 | 50931 |
| Il2rg | 270176 | 2.966821 | 16186 |
| Il2rg | 1850402 | 2.0477679 |  |
| Il33 | 3440767 | 7.311128 | 77125 |
| Il4 | 1300445 | 3.0392501 | 16189 |
| Il4i1 | 6020224 | 9.3868885 | 14204 |
| Il7r | 1660504 | 2.1401904 | 16197 |
| Inhba | 3060458 | 2.4899845 | 16323 |
| Inpp5d | 4040523 | 2.8803837 | 16331 |
| Irf4 | 1010397 | 5.7705245 | 16364 |
| Irf5 | 3140646 | 2.3384018 | 27056 |
| Irf7 | 6590653 | 2.4529874 | 54123 |
| Irf8 | 4610373 | 2.5720568 | 15900 |
| Itgal | 1170091 | 3.2439673 | 16408 |
| Itgb7 | 830543 | 4.223733 | 16421 |
| Itk | 6180154 | 2.1177557 | 16428 |
| Jak1 | 6650039 | -2.2403462 | 16451 |
| Jak3 | 4850086 | 2.1327991 | 16453 |
| Jam4 | 2370246 | -2.2419949 | 72058 |
| Kcnf1 | 7380228 | -2.7405746 | 382571 |
| Kif21b | 4670100 | 2.6283336 | 16565 |
| Klhl6 | 5490358 | 4.381643 | 239743 |
| Klhl6 | 6860040 | 4.22929 | 239743 |
| Klk1 | 2760181 | 4.624776 | 16612 |
| Klk1b26 | 6100324 | 3.4381013 | 16618 |
| Klk1b27 | 1820731 | 2.2917519 | 16619 |
| Klk1b27 | 4670021 | 2.0307665 | 16619 |
| Klk1b4 | 6040626 | 2.238915 | 18048 |
| Klk1b5 | 2060136 | 2.347281 | 16622 |
| Klrd1 | 4890279 | 2.4650903 | 16643 |
| Kmo | 6560717 | 2.175395 | 98256 |
| Kndc1 | 6960376 | 2.5057592 | 76484 |
| Krt13 | 4610414 | 8.133385 | 16663 |
| Krt14 | 1470619 | 26.106651 | 16664 |
| Krt20 | 4610327 | -2.0958874 | 66809 |
| Krt4 | 4920438 | 6.0087066 | 16682 |
| Krt6b | 1090730 | 3.4939065 | 16688 |
| Krtdap | 830333 | -2.7113402 | 64661 |
| Krtdap | 610707 | -2.202891 |  |
| Kynu | 520138 | 2.5018218 | 70789 |
| LOC100039742 | 6220274 | 4.9400697 | 100039742 |
| LOC100040671 | 2940673 | -2.1752942 | 100040671 |
| LOC100041103 | 7400400 | -2.4220512 | 100041103 |
| LOC100041137 | 4180048 | 2.1304848 | 100041137 |
| LOC100041504 | 6420376 | 3.6479042 | 100041504 |
| LOC100041569 | 3800300 | -2.8348103 | 100041569 |
| LOC100042270 | 1770301 | 21.062273 | 100042270 |
| LOC100043918 | 1740196 | 2.5021896 | 100043918 |
| LOC100043991 | 4810392 | 84.01088 | 100043991 |
| LOC100044439 | 6020487 | 9.790565 | 100044439 |
| LOC100044538 | 4180537 | 4.4536347 | 100044538 |
| LOC100044683 | 1580520 | -2.1945608 | 100044683 |
| LOC100044702 | 6180202 | 4.4413104 | 100044702 |
| LOC100045280 | 2120377 | -2.0776925 | 100045280 |
| LOC100045341 | 6250315 | 3.504665 | 100045341 |
| LOC100045680 | 1010632 | 4.0346828 | 100045680 |
| LOC100045877 | 3120037 | 6.2609553 | 100045877 |
| LOC100046087 | 4860482 | 2.2715404 | 100046087 |
| LOC100046120 | 1300747 | 2.1842992 | 100046120 |
| LOC100046129 | 1770441 | -2.1277623 | 100046129 |
| LOC100046250 | 1690047 | 4.209942 | 100046250 |
| LOC100046275 | 4260735 | 51.286243 | 100046275 |
| LOC100046496 | 3780368 | 42.840168 | 100046496 |
| LOC100046546 | 1450538 | 51.3692 | 100046546 |
| LOC100046552 | 1770767 | 20.695353 | 100046552 |
| LOC100046690 | 6650424 | -2.1090162 | 100046690 |
| LOC100046793 | 6330239 | 230.11723 | 100046793 |
| LOC100046793 | 6960673 | 202.05986 | 100046793 |
| LOC100047053 | 5700347 | 77.08192 | 100047053 |
| LOC100047053 | 6180747 | 96.178925 | 100047053 |
| LOC100047132 | 2940167 | 3.7037735 | 100047132 |
| LOC100047132 | 520343 | 7.9849057 | 100047132 |
| LOC100047162 | 6840279 | 96.66905 | 100047162 |
| LOC100047316 | 6270072 | 221.40968 | 100047316 |
| LOC100047316 | 2690538 | 30.356077 | 100047316 |
| LOC100047579 | 4230368 | -2.7500591 | 100047579 |
| LOC100047583 | 1400053 | 3.4158905 | 100047583 |
| LOC100047619 | 4180437 | 2.0357673 | 100047619 |
| LOC100047628 | 1240333 | 38.094395 | 100047628 |
| LOC100047628 | 3400100 | 16.381723 | 100047628 |
| LOC100047788 | 2760243 | 61.365013 | 100047788 |
| LOC100047788 | 4810040 | 69.87257 | 100047788 |
| LOC100047815 | 1340553 | 13.875675 | 100047815 |
| LOC100048554 | 2480296 | 7.086727 | 100048554 |
| LOC100048556 | 2510333 | 2.9697864 | 100048556 |
| LOC100048556 | 1990221 | 3.77077 | 100048556 |
| LOC100048770 | 4670091 | 26.820602 | 100048770 |
| LOC207685 | 5860133 | 105.37839 | 207685 |
| LOC207685 | 6250753 | 16.654419 | 207685 |
| LOC207685 | 5420128 | 20.797846 | 207685 |
| LOC213684 | 3780639 | 189.92052 |  |
| LOC218617 | 6580047 | 2.6544528 |  |
| LOC226017 | 3370286 | -3.0401814 |  |
| LOC232060 | 1030373 | 143.18674 |  |
| LOC232065 | 1710446 | 31.239347 |  |
| LOC232067 | 2000014 | 16.026093 |  |
| LOC238447 | 6760307 | 130.69601 |  |
| LOC243431 | 4220100 | 52.671608 | 243431 |
| LOC243439 | 6550477 | 42.57416 | 243439 |
| LOC243453 | 4880520 | 2.6542137 |  |
| LOC268853 | 2650403 | -2.0068822 |  |
| LOC272683 | 5490156 | 63.93034 |  |
| LOC329575 | 2760519 | -2.0538335 | 329575 |
| LOC380799 | 2750113 | 99.283394 |  |
| LOC380801 | 2370386 | 39.73035 |  |
| LOC380805 | 5310040 | 10.086356 |  |
| LOC381140 | 5900370 | 2.4614081 |  |
| LOC381774 | 1580750 | 129.68712 |  |
| LOC381782 | 4890176 | 5.8782845 |  |
| LOC382646 | 2100564 | 4.189285 |  |
| LOC382646 | 6980681 | 4.4869742 |  |
| LOC382646 | 430139 | 2.6709886 |  |
| LOC383196 | 6110220 | 74.451195 | 383196 |
| LOC383196 | 5870358 | 66.88516 | 383196 |
| LOC384411 | 1660753 | 9.505838 |  |
| LOC384413 | 1050731 | 230.49716 |  |
| LOC384415 | 610632 | 88.2043 |  |
| LOC384419 | 6580309 | 59.01824 |  |
| LOC384422 | 2340022 | 52.415833 |  |
| LOC385109 | 7200039 | 164.31432 |  |
| LOC385277 | 7380064 | 45.500923 |  |
| LOC385291 | 3870307 | 204.39995 |  |
| LOC385644 | 60132 | -2.1666658 |  |
| LOC386520 | 6220471 | 36.473976 |  |
| LOC432709 | 2570743 | 17.248407 | 432709 |
| LOC432709 | 620719 | 42.840946 | 432709 |
| LOC433943 | 6860070 | -3.2836378 | 433943 |
| LOC434026 | 5860687 | 19.17907 | 434026 |
| LOC434031 | 3890685 | 111.049324 | 434031 |
| LOC434609 | 5690433 | 2.989848 | 434609 |
| LOC544904 | 780064 | 13.253572 | 544904 |
| LOC545013 | 6180379 | -2.2803278 | 545013 |
| LOC624610 | 6400347 | 4.815285 | 624610 |
| LOC626347 | 110523 | 16.023678 | 626347 |
| LOC626583 | 1260634 | 19.732426 | 626583 |
| LOC630242 | 7400692 | 14.820822 | 630242 |
| LOC630253 | 6330193 | 89.3197 | 630253 |
| LOC630284 | 1780370 | 190.97026 | 630284 |
| LOC630284 | 1300195 | 62.4506 | 630284 |
| LOC630302 | 460521 | 59.21408 | 630302 |
| LOC630302 | 2850228 | 109.58489 | 630302 |
| LOC630305 | 4640364 | 142.42451 | 630305 |
| LOC630305 | 3190154 | 66.31594 | 630305 |
| LOC630320 | 4730528 | 28.2751 | 630320 |
| LOC630337 | 3290139 | 130.67712 | 630337 |
| LOC630347 | 4890471 | 15.66585 | 630347 |
| LOC630837 | 2230129 | 98.99164 | 630837 |
| LOC633273 | 3870240 | 6.48771 | 633273 |
| LOC634749 | 7650215 | 3.5168161 | 634749 |
| LOC635601 | 450327 | 50.02307 | 635601 |
| LOC635601 | 5810575 | 57.776516 | 635601 |
| LOC635815 | 7200672 | 46.779415 | 635815 |
| LOC635860 | 5080382 | 2.5175958 | 635860 |
| LOC636017 | 620768 | 3.4678025 | 636017 |
| LOC636677 | 6480100 | 4.7809563 | 636677 |
| LOC636696 | 5700241 | 142.65546 | 636696 |
| LOC636752 | 20132 | 194.17235 | 636752 |
| LOC636818 | 3290543 | 9.004586 | 636818 |
| LOC636875 | 5050703 | 27.784693 | 636875 |
| LOC636944 | 7400497 | 265.0333 | 636944 |
| LOC636944 | 1990706 | 220.74016 | 636944 |
| LOC637155 | 5900010 | 9.033676 | 637155 |
| LOC637227 | 2190021 | 123.51264 | 637227 |
| LOC637260 | 2260504 | 26.530674 | 637260 |
| LOC637337 | 6520706 | 18.006287 | 637337 |
| LOC637785 | 3850241 | 24.099997 | 637785 |
| LOC638301 | 6450259 | 2.9937673 | 638301 |
| LOC640340 | 7650193 | 27.459301 | 640340 |
| LOC640696 | 5390553 | 15.240027 | 640696 |
| LOC641221 | 4060324 | 17.907585 | 641221 |
| LOC641366 | 3890376 | 2.4147635 | 641366 |
| LOC665506 | 6760735 | 5.9626856 | 665506 |
| LOC666053 | 6900504 | -2.1864278 | 666053 |
| LOC669053 | 5690300 | 110.74137 | 669053 |
| LOC672329 | 6520437 | 139.47583 | 672329 |
| LOC672339 | 4570408 | 144.10086 | 672339 |
| LOC672342 | 1070356 | 151.76463 | 672342 |
| LOC674072 | 3450392 | 2.5646243 | 674072 |
| LOC674087 | 1050142 | 64.65016 | 674087 |
| LOC674089 | 1500376 | 15.75284 | 674089 |
| LOC674094 | 6980168 | 18.321789 | 674094 |
| LOC674107 | 10367 | 10.156769 | 674107 |
| LOC674110 | 1240347 | 104.62242 | 674110 |
| LOC674114 | 3830435 | 45.635723 | 674114 |
| LOC674147 | 2230112 | 47.156612 | 674147 |
| LOC674147 | 1090376 | 56.330246 | 674147 |
| LOC675759 | 7570075 | 5.3899374 | 675759 |
| LOC675759 | 4780301 | 7.7801213 | 675759 |
| LOC676136 | 3190687 | 64.45773 | 676136 |
| LOC676136 | 7000202 | 85.62635 | 676136 |
| LOC676222 | 4780379 | 170.0123 | 676222 |
| LOC676420 | 1190377 | 2.3933334 | 676420 |
| LOC677643 | 50064 | 37.046936 | 677643 |
| LOC677648 | 610433 | 63.548878 | 677648 |
| Laptm5 | 940438 | 3.8637745 | 16792 |
| Lat | 6900044 | 2.5290854 | 16797 |
| Lat2 | 6370553 | 5.003636 | 56743 |
| Lax1 | 2810722 | 3.1629133 | 240754 |
| Lck | 1820541 | 3.5393395 | 16818 |
| Lcp2 | 2350241 | 2.2576632 |  |
| Lcp2 | 5290647 | 2.7545815 | 16822 |
| Lims2 | 3180681 | -2.0288298 | 225341 |
| Lmo2 | 7570537 | 2.1217515 | 16909 |
| Lpxn | 7050433 | 3.6358523 | 107321 |
| Lrmp | 5390064 | 2.9490094 | 16970 |
| Lrmp | 5340369 | 5.3749137 |  |
| Lrrc33 | 7050743 | 2.1948013 | 224109 |
| Lst1 | 5860154 | 3.9726198 |  |
| Lta | 2900474 | 2.3962936 | 16992 |
| Ltb | 160408 | 3.3602855 | 16994 |
| Ltf | 6940037 | 2.1906028 | 17002 |
| Ly6c1 | 5550671 | 3.715337 | 17067 |
| Ly78 | 2810220 | 2.5610237 |  |
| Ly86 | 430167 | 4.00857 | 17084 |
| Lyl1 | 360195 | 2.6885555 | 17095 |
| Lypd6 | 520692 | -2.0504832 | 320343 |
| Lypd6b | 110326 | -2.1021988 | 71897 |
| Lypla1 | 6560543 | -2.2379062 | 18777 |
| MALT-1 | 4070768 | 2.743515 |  |
| Map4k1 | 4010097 | 4.215558 | 26411 |
| Marveld3 | 4590110 | -2.0245082 | 73608 |
| Matk | 1940646 | 2.0376866 | 17179 |
| Mcpt1 | 2680215 | 51.921627 | 17224 |
| Mcpt4 | 1820367 | 7.075755 | 17227 |
| Mcpt6 | 5670441 | 6.0009694 | 17229 |
| Mef2c | 1230440 | 3.824304 | 17260 |
| Mef2c | 5820369 | 4.481771 | 17260 |
| Mef2c | 2570427 | 2.819627 | 17260 |
| Mela | 3940129 | 2.563296 |  |
| Mfap4 | 20075 | 2.165026 | 76293 |
| Mfap5 | 1740181 | -2.3280144 | 50530 |
| Mfge8 | 2120286 | 2.3641138 |  |
| Mgl2 | 1010047 | 2.076411 |  |
| Mgst1 | 3190438 | -2.4222076 | 56615 |
| Mist1 | 2030669 | 2.8494952 |  |
| Mki67 | 5360129 | 2.687908 | 17345 |
| Mll5 | 4200706 | 2.2098105 |  |
| Mmp10 | 3890600 | 11.537178 | 17384 |
| Mmp13 | 5690131 | 5.753279 | 17386 |
| Mmp3 | 6180544 | 2.657511 | 17392 |
| Mmp3 | 1510750 | 3.5116363 | 17392 |
| Mmrn1 | 1430747 | 2.251398 | 70945 |
| Mocs1 | 60600 | -2.433266 | 56738 |
| Mpeg1 | 4010221 | 2.0908875 | 17476 |
| Ms4a6b | 3610286 | 2.33458 | 69774 |
| Ms4a7 | 1240553 | 2.519802 | 109225 |
| Ms4a7 | 1660528 | 3.6405544 | 109225 |
| Msc | 1230746 | 2.1766648 | 17681 |
| Msn | 1780474 | 2.505686 |  |
| Myh10 | 7040608 | -2.2139354 | 77579 |
| Myh2 | 5490528 | -3.8029284 |  |
| Myo1f | 4860719 | 2.2987158 | 17916 |
| Myo1g | 7200356 | 2.587816 | 246177 |
| Myoc | 7400463 | -2.2635996 | 17926 |
| Myocd | 6760673 | -2.0069768 | 214384 |
| Napsa | 5690500 | 4.8771157 | 16541 |
| Napsa | 380369 | 4.689568 | 16541 |
| Napsa | 4830551 | 2.5541947 | 16541 |
| Ncf1 | 3520300 | 2.1275146 | 17969 |
| Ncf4 | 2940504 | 2.9476779 | 17972 |
| Nckap1l | 940202 | 2.9908113 | 105855 |
| Ndrg2 | 1450601 | -2.8728163 | 29811 |
| Nfatc1 | 450491 | 2.3567548 | 18018 |
| Nfe2 | 1170170 | 2.059409 | 18022 |
| Nfe2l2 | 1110201 | -2.115083 | 18024 |
| Nfe2l3 | 2480204 | -2.3078403 | 18025 |
| Nfkb1 | 5050541 | 2.2160957 |  |
| Nfkbie | 6420450 | 2.347387 | 18037 |
| Ng23 | 3460326 | 2.8737175 | 78376 |
| Nipsnap1 | 3990397 | -2.2227373 | 18082 |
| Nkg7 | 4040035 | 2.127126 | 72310 |
| Nope | 2230687 | -2.1119204 | 56741 |
| Npm3-ps1 | 7210709 | 2.285653 | 108176 |
| Nptx2 | 3120497 | -2.3204722 | 53324 |
| Npy6r | 520411 | -4.1854877 | 18169 |
| Nqo1 | 4010673 | -2.400622 | 18104 |
| Nrarp | 4810328 | -2.3105233 | 67122 |
| Nsdhl | 2650653 | -2.4967594 | 18194 |
| Nt5c2 | 870433 | -2.0083578 | 76952 |
| Nuak2 | 1770592 | 3.0870283 | 74137 |
| Nup210 | 1230703 | 4.5931506 | 54563 |
| Nusap1 | 1500491 | 2.2078037 | 108907 |
| ORF9 | 4810259 | 2.7982397 | 52793 |
| ORF9 | 1230653 | 2.7803075 | 52793 |
| OTTMUSG00000000971 | 5360370 | 2.5650847 | 100034251 |
| Oas1g | 3890328 | 2.372511 | 23960 |
| Obfc2a | 610273 | -2.7129664 | 109019 |
| Oosp1 | 1230524 | 7.3268886 | 170834 |
| P2ry14 | 1240538 | 2.4585917 | 140795 |
| P2ry14 | 6580379 | 3.2739115 | 140795 |
| Padi1 | 1690201 | 4.5185466 | 18599 |
| Parvg | 3310612 | 2.440548 | 64099 |
| Pcbp3 | 1300681 | -2.0784378 | 59093 |
| Pdk4 | 4120131 | -2.6068206 | 27273 |
| Pdlim3 | 2000482 | -2.017351 | 53318 |
| Peg3 | 2690435 | -2.726782 | 18616 |
| Pfkp | 2900482 | -2.1507797 | 56421 |
| Pfn2 | 290672 | -2.1090395 | 18645 |
| Pfn2 | 5720241 | -2.5821817 | 18645 |
| Pglyrp1 | 3140246 | 3.3537493 |  |
| Pgm5 | 3400719 | -2.0938897 | 226041 |
| Pgm5 | 7100504 | -2.5281303 | 226041 |
| Pi16 | 1230546 | -2.3676805 | 74116 |
| Pigr | 6510373 | 2.3918464 | 18703 |
| Pik3ap1 | 770189 | 2.5641282 | 83490 |
| Pik3cd | 6560017 | 2.7886386 | 18707 |
| Pik3cg | 3450075 | 3.225792 | 30955 |
| Pik3cg | 4850291 | 2.194051 |  |
| Pip4k2a | 3140678 | 2.4985888 | 18718 |
| Pira3 | 5870113 | 4.9927692 | 18726 |
| Pira4 | 7330114 | 2.2379797 | 18727 |
| Pkib | 2370026 | 2.047157 | 18768 |
| Pkp1 | 3940070 | 2.4375443 | 18772 |
| Pla1a | 4210372 | 2.6061091 | 85031 |
| Plac8 | 5860243 | 3.2663221 | 231507 |
| Plce1 | 940519 | -2.0711617 | 74055 |
| Plcg2 | 1940255 | 3.5533173 | 234779 |
| Pld4 | 5720255 | 3.2847867 | 104759 |
| Plek | 1820075 | 2.7911253 | 56193 |
| Plvap | 3290239 | 2.4462318 | 84094 |
| Pon3 | 610400 | 4.206362 | 269823 |
| Pon3 | 3460364 | 2.340263 | 269823 |
| Pou2af1 | 1690392 | 23.993803 | 18985 |
| Pou2f2 | 6370253 | 2.6739423 | 18987 |
| Pparg | 6380066 | -2.0714233 | 19016 |
| Ppfibp2 | 430403 | -2.1980503 | 19024 |
| Ppfibp2 | 4850097 | -2.2641013 | 19024 |
| Ppp3cc | 4590470 | 2.0361927 | 19057 |
| Prc1 | 780475 | 2.1566772 | 233406 |
| Prdx4 | 2000180 | 2.0606444 | 53381 |
| Prg2 | 4280180 | 4.886619 | 19074 |
| Prom2 | 6650670 | -2.3027694 | 192212 |
| Prom2 | 7160228 | -3.1443825 | 192212 |
| Prss8 | 2650601 | -3.1776772 | 76560 |
| Prss8 | 6350291 | -2.1124756 | 76560 |
| Pstpip1 | 2360554 | 2.4067028 | 19200 |
| Ptpn18 | 4050689 | 3.0730712 | 19253 |
| Ptpn22 | 5900593 | 3.5854144 | 19260 |
| Ptpn6 | 4060240 | 4.394998 | 15170 |
| Ptpn6 | 2810541 | 3.06307 | 15170 |
| Ptprc | 1230541 | 3.8437843 | 19264 |
| Ptprcap | 6840360 | 2.520442 | 19265 |
| Ptprz1 | 1690239 | -2.091204 | 19283 |
| Punc | 2450100 | -2.2146235 | 19289 |
| Pvrl3 | 5670465 | -2.2848663 | 58998 |
| Qpct | 990687 | -2.0337362 | 70536 |
| Rab11a | 1010110 | -2.0005693 | 53869 |
| Rab25 | 630451 | -2.074021 | 53868 |
| Rab27b | 1190706 | -2.8832724 | 80718 |
| Rab7 | 870167 | -2.068299 | 19349 |
| Rac2 | 4490243 | 4.8093 | 19354 |
| Rapgefl1 | 1010524 | -2.662328 | 268480 |
| Rasgrp1 | 5860296 | 4.0066986 | 19419 |
| Rasgrp3 | 510010 | 2.8075128 | 240168 |
| Rasl11b | 290341 | -2.0075853 | 68939 |
| Rassf2 | 5960487 | 2.08337 | 215653 |
| Rassf5 | 6590044 | 2.1167731 | 54354 |
| Rassf7 | 6040747 | -2.0972924 | 66985 |
| Rbbp7 | 2650598 | -2.4148865 | 245688 |
| Rbbp7 | 670711 | -2.0669658 | 245688 |
| Rbbp7 | 6590445 | -2.3522427 | 245688 |
| Rcsd1 | 6590296 | 2.620171 | 226594 |
| Reg3g | 830619 | 3.7141511 | 19695 |
| Reln | 7610484 | 2.8203447 | 19699 |
| Retnla | 1110709 | 6.8373227 | 57262 |
| Retnlg | 7160711 | 31.124002 | 245195 |
| Rftn1 | 5560349 | 2.29903 | 76438 |
| Rgs11 | 3290725 | -2.0939653 |  |
| Rhof | 3780192 | 3.2647386 | 23912 |
| Rims1 | 2070402 | -2.1004832 | 116837 |
| Rin3 | 1990204 | 2.4314442 | 217835 |
| Ripk4 | 3850086 | -2.3867536 | 72388 |
| Rnase6 | 430703 | 4.7149906 | 78416 |
| Rnf208 | 2570603 | -2.1643116 | 68846 |
| Rock2 | 2370575 | -2.0277414 | 19878 |
| Rsph1 | 4760066 | 3.7751875 | 22092 |
| Rtn4 | 7040554 | -2.2989576 | 68585 |
| S100a1 | 940338 | -2.5604267 | 20193 |
| S100a8 | 1190546 | 8.906053 | 20201 |
| S100a9 | 1980603 | 6.574192 | 20202 |
| Samsn1 | 2030543 | 3.0133367 | 67742 |
| Samsn1 | 6020400 | 4.682164 | 67742 |
| Scin | 3800671 | 2.2676797 | 20259 |
| Scnn1b | 7210600 | -2.2317193 | 20277 |
| Scnn1g | 7100255 | -3.937877 | 20278 |
| Sdcbp2 | 940010 | -2.0881739 | 228765 |
| Sdf2l1 | 3360010 | 2.3282473 | 64136 |
| Sec11c | 5130497 | 2.2686093 | 66286 |
| Sel1h | 5870286 | 9.019605 |  |
| Sel1l | 5260019 | 3.0181525 | 20338 |
| Sele | 4120333 | -2.4826193 | 20339 |
| Sell | 5360707 | 5.837712 | 20343 |
| Selpl | 3800070 | 2.1784036 |  |
| Selplg | 1070307 | 3.813331 | 20345 |
| Sema4g | 5810253 | -2.0551171 | 26456 |
| Serpina1b | 2650463 | 3.4965403 |  |
| Serpina1b | 1430253 | 3.3462446 | 20701 |
| Serpina1b | 2120152 | 4.0083504 | 20701 |
| Serpina1d | 2690364 | 3.2647462 | 20703 |
| Serpina3g | 1050092 | 8.882269 | 20715 |
| Serpina3h | 1450491 | 5.4538903 | 546546 |
| Serpina3n | 2450347 | 4.209728 | 20716 |
| Serpine2 | 870309 | 3.9900503 |  |
| Sfrp1 | 4060398 | 2.2170923 |  |
| Sh2d2a | 4760575 | 2.8113256 | 27371 |
| Sh3bgr | 460750 | -2.17636 |  |
| Sh3bgrl2 | 5270220 | -2.0685341 | 212531 |
| Sh3gl2 | 1400689 | -3.3861198 | 20404 |
| Sh3gl2 | 3870390 | -4.520686 | 20404 |
| Sh3gl2 | 3840136 | -3.8192735 | 20404 |
| Sh3tc2 | 3180307 | -2.1486444 | 225608 |
| Shh | 2260450 | -2.6901395 | 20423 |
| Shh | 4560113 | -2.4977267 | 20423 |
| Siglec1 | 4570458 | 2.3667793 | 20612 |
| Siglecg | 2850553 | 5.8518286 | 243958 |
| Sirpa | 3400646 | 2.270585 | 19261 |
| Sla | 110064 | 4.764401 | 20491 |
| Slamf9 | 7510291 | 4.1623726 | 98365 |
| Slc12a7 | 7550403 | -2.1586485 | 20499 |
| Slc15a3 | 3290427 | 4.4368744 | 65221 |
| Slc16a6 | 3120427 | 4.341128 | 104681 |
| Slc16a6 | 5860475 | 4.244989 | 104681 |
| Slc18a2 | 4480192 | 2.3838878 | 214084 |
| Slc2a3 | 460327 | 2.0066166 | 20527 |
| Slc44a3 | 6590110 | -2.3876984 | 213603 |
| Slc47a1 | 1260129 | -2.3478322 | 67473 |
| Slc7a7 | 7320349 | 2.2194092 | 20540 |
| Slco4a1 | 5820270 | 2.5019646 | 108115 |
| Slfn2 | 4120307 | 2.3067315 | 20556 |
| Slmap | 6900228 | -2.0892615 | 83997 |
| Slmap | 6580239 | -2.6935053 | 83997 |
| Slpi | 2810487 | 93.32103 | 20568 |
| Smox | 5050008 | 2.0747137 | 228608 |
| Smox | 1660367 | 2.1869757 | 228608 |
| Snhg11 | 4220288 | -3.5663953 | 319317 |
| Snhg11 | 2480730 | -2.8678567 | 319317 |
| Snurf | 620209 | -2.4322255 | 84704 |
| Snurf | 3130246 | -2.2079113 | 84704 |
| Socs2 | 4210129 | 2.5331542 | 216233 |
| Spcs3 | 5700537 | 4.798075 | 76687 |
| Spp1 | 1170139 | 4.097159 | 20750 |
| Sprr2f | 2760669 | 25.138237 | 20760 |
| Sprr2g | 4220037 | 6.98878 | 20761 |
| Sptlc3 | 60072 | -2.2928212 | 228677 |
| Sqle | 290168 | -2.4086418 | 20775 |
| Srd5a1 | 1090474 | -3.0257232 |  |
| Srgn | 4610433 | 3.4919949 | 19073 |
| Srgn | 5490187 | 4.027544 | 19073 |
| Srp54 | 1050273 | -2.2840972 | 24067 |
| Srpk3 | 20465 | 3.4134555 | 56504 |
| St6gal1 | 1430368 | 2.924735 | 20440 |
| St8sia4 | 3830553 | 2.1898575 | 20452 |
| Stac2 | 610047 | 2.7799048 | 217154 |
| Stard10 | 1940612 | -2.00754 | 56018 |
| Stat3 | 6660176 | 2.0744948 | 20848 |
| Stat4 | 5560066 | 2.920427 | 20849 |
| Stfa1 | 940717 | 8.158489 | 20861 |
| Stfa1 | 3360056 | 6.311148 | 20861 |
| Stk17b | 6270091 | 3.3946555 | 98267 |
| Swap70 | 4730392 | 2.228002 | 20947 |
| Swap70 | 3310132 | 2.013178 | 20947 |
| Swap70 | 1990041 | 2.1490474 | 20947 |
| Sytl2 | 3710563 | -2.3938339 | 83671 |
| Taf15 | 3830364 | 2.535453 | 70439 |
| Tagln | 6130411 | -2.624402 | 21345 |
| Tbc1d9 | 2690243 | -2.9418554 | 71310 |
| Tcirg1 | 2570301 | 2.4157026 | 27060 |
| Tcirg1 | 4760324 | 2.3510273 | 27060 |
| Tcrb-V8.2 | 2510138 | 3.9987266 |  |
| Tes | 7550121 | -2.0006788 | 21753 |
| Tes | 4210138 | -2.035034 | 21753 |
| Timp1 | 4640215 | 3.4133332 |  |
| Tlr1 | 6940719 | 2.5861034 | 21897 |
| Tmem159 | 3060056 | -2.0408847 | 233806 |
| Tmem184a | 430504 | -2.9693713 | 231832 |
| Tmem45b | 7570053 | -2.3356004 | 235135 |
| Tmem79 | 3120719 | -2.3672884 | 71913 |
| Tmem86a | 2750184 | 2.0380788 | 67893 |
| Tmprss13 | 1300220 | -2.553735 | 214531 |
| Tmprss2 | 4070279 | -2.2700033 | 50528 |
| Tnfrsf13b | 4280296 | 6.6017265 |  |
| Tnfrsf13c | 2710520 | 7.9045672 | 72049 |
| Tnfrsf17 | 2940482 | 4.008781 | 21935 |
| Tnfrsf18 | 3130639 | 2.1260664 |  |
| Tnfrsf18 | 3180156 | 2.0332522 | 21936 |
| Tnfsf12-tnfsf13 | 2340543 | 2.063902 | 619441 |
| Tnfsf13b | 6840059 | 2.991223 | 24099 |
| Tnfsf13b | 610661 | 3.2378552 |  |
| Tph1 | 130066 | 3.7949061 | 21990 |
| Tpm2 | 1070088 | 2.999871 | 22004 |
| Tpsab1 | 5130553 | 2.541806 | 17230 |
| Traf1 | 2510164 | 2.7842503 |  |
| Trf | 7040044 | -2.362811 | 22041 |
| Trp53inp1 | 5260201 | 2.3508663 | 60599 |
| Trp53inp1 | 60291 | 3.81245 | 60599 |
| Tsku | 2030386 | -2.0630853 | 244152 |
| Tspan32 | 830377 | 3.769913 | 27027 |
| Tssc8 | 6900609 | 2.0214083 |  |
| Ttr | 2680113 | -9.775843 | 22139 |
| Tyrobp | 450739 | 2.8313937 | 22177 |
| Ubd | 7040170 | 2.8997722 | 24108 |
| Ugcg | 4210440 | 2.2140641 |  |
| Ugcg | 1230564 | 2.1602325 | 22234 |
| Ugt1a10 | 6330372 | -2.2645817 | 394430 |
| Ugt1a10 | 2350131 | -2.0520222 | 394430 |
| Ugt1a10 | 2350730 | -2.543157 | 394430 |
| Ugt1a6a | 3370669 | -5.059047 | 94284 |
| Ugt1a6a | 1170349 | -2.266657 | 94284 |
| Ugt1a6a | 3800446 | -4.5601077 | 94284 |
| Ugt1a6b | 3310138 | -4.7718663 | 394435 |
| Ugt1a7c | 650554 | -2.0673783 | 394432 |
| Ugt1a7c | 4060044 | -2.4267306 | 394432 |
| Uhrf1 | 4560397 | 2.470656 | 18140 |
| Unc93b1 | 2100689 | 2.6618257 | 54445 |
| Unc93b1 | 5090403 | 2.137874 | 54445 |
| Upk1a | 1110762 | -2.760085 | 109637 |
| Upk1a | 2970164 | -3.658247 | 109637 |
| Upk2 | 2650554 | -3.294826 | 22269 |
| Upk3a | 630097 | -3.1179998 | 22270 |
| Upk3b | 6270670 | -2.7152345 | 100647 |
| Upk3b | 4040243 | -2.6621206 | 100647 |
| Upk3b | 520333 | -2.5813513 | 100647 |
| Vars | 1580044 | 2.017964 | 22321 |
| Vav1 | 5270608 | 3.5055354 | 22324 |
| Vldlr | 5900577 | -2.0935092 | 22359 |
| Vpreb3 | 4900500 | 3.701106 | 22364 |
| Vrk1 | 3610731 | 2.141697 |  |
| Vsig2 | 3990593 | -2.9869149 | 57276 |
| Vsig2 | 5270347 | -2.397952 | 57276 |
| Was | 5340180 | 3.928692 | 22376 |
| Wfdc15b | 110292 | -4.1148896 | 192201 |
| Wfdc15b | 2600017 | -3.5324407 | 192201 |
| Wnt5a | 7400706 | -2.035869 | 22418 |
| Wnt7b | 3400202 | -2.062323 | 22422 |
| Xbp1 | 3450100 | 2.422036 | 22433 |
| Xlr4a | 5870021 | 2.7365832 | 434794 |
| Zbtb8b | 3440386 | -2.0039837 | 215627 |
| Zc3h12d | 6510142 | 4.5241685 | 237256 |
| Zcchc18 | 3890750 | 2.0173209 | 66995 |
| Zdhhc13 | 5560079 | -2.3171997 | 243983 |
| Zdhhc9 | 3450427 | -2.3317862 | 208884 |
| Zfp339 | 270682 | -2.370232 |  |
| Zfpn1a1 | 5810048 | 2.840485 |  |
| mt-Nd4l | 3060209 | -2.8249917 |  |
| mtDNA_ND4L | 2680520 | -2.3311503 |  |
| scl0001163.1_8 | 5490730 | -2.108342 |  |
| scl0001849.1_2273 | 6370020 | 5.83469 |  |
| scl0001883.1_112 | 1690343 | -2.727567 |  |
